# Genome-Wide Characterization and Analysis of bHLH Transcription Factors Related to Anthocyanin Biosynthesis in Fig (*Ficus carica* L.)

**DOI:** 10.3389/fpls.2021.730692

**Published:** 2021-10-08

**Authors:** Miaoyu Song, Haomiao Wang, Zhe Wang, Hantang Huang, Shangwu Chen, Huiqin Ma

**Affiliations:** ^1^College of Horticulture, China Agricultural University, Beijing, China; ^2^College of Food Science and Nutritional Engineering, China Agricultural University, Beijing, China; ^3^State Key Laboratory of Agrobiotechnology, China Agricultural University, Beijing, China

**Keywords:** genome-wide, bHLH transcription factors, expression analysis, anthocyanin biosynthesis, *Ficus carica* L.

## Abstract

The basic helix–loop–helix (bHLH) transcription factor family is the second largest transcription factor family in plants, and participates in various plant growth and development processes. A total of 118 bHLH genes were identified from fig (*Ficus carica* L.) by whole-genome database search. Phylogenetic analysis with *Arabidopsis* homologs divided them into 25 subfamilies. Most of the *bHLH*s in each subfamily shared a similar gene structure and conserved motifs. Seventy-two *bHLH*s were found expressed at fragments per kilobase per million mapped (FPKM) > 10 in the fig fruit; among them, 15 *bHLH*s from eight subfamilies had FPKM > 100 in at least one sample. *bHLH* subfamilies had different expression patterns in the female flower tissue and peel during fig fruit development. Comparing green and purple peel mutants, 13 bHLH genes had a significantly different (≥ 2-fold) expression. Light deprivation resulted in 68 significantly upregulated and 22 downregulated bHLH genes in the peel of the fruit. Sixteen bHLH genes in subfamily III were selected by three sets of transcriptomic data as candidate genes related to anthocyanin synthesis. Interaction network prediction and yeast two-hybrid screening verified the interaction between *FcbHLH42* and anthocyanin synthesis-related genes. The transient expression of *FcbHLH42* in tobacco led to an apparent anthocyanin accumulation. Our results confirm the first fig bHLH gene involved in fruit color development, laying the foundation for an in-depth functional study on other FcbHLH genes in fig fruit quality formation, and contributing to our understanding of the evolution of bHLH genes in other horticulturally important *Ficus* species.

## Introduction

Transcription factors are key regulatory elements in life processes (Yamasaki et al., 2013; Guo and Wang, 2017). To date, more than 60 transcription factor families have been found in plants. According to the number of lysine and arginine residues in the DNA-binding domain, transcription factors are divided into four categories: zinc finger (ZF) type, helix–turn–helix (HLH), basic helix–loop–helix (bHLH), and basic leucine zipper (bZIP). The most commonly found transcription factors in higher plants are members of the WD40, MYB, WRKY, bHLH, and bZIP families (Kosugi and Ohashi, 2002).

The bHLH transcription factors, also called MYCs, form the second largest family of transcription factors in plants (Feller et al., 2011), with 162, 95, 167, and 152 bHLH genes identified in *Arabidopsis* (Bailey et al., 2003), grape (Wang et al., 2018), rice (Li et al., 2006), and tomato (Wang et al., 2015), respectively. The bHLH domain is approximately 60 amino acids long, containing a basic region and an HLH region. The basic region, located next to the N-terminus, contains the DNA *cis*-acting elements E-box (5'-CANNTG-3') and G-box (5'-CACGTG-3') that regulate gene expression, whereas the HLH region consists of two amphipathic α-helices linked by a loop that serve as the dimerization domain to promote protein interactions, producing homodimers or heterodimers (Massari and Murre, 2000). bHLHs can act as either repressors or activators of gene transcription and play important roles in various physiological processes, such as sexual maturation, metabolism, and development (Feller et al., 2011).

According to evolutionary relationships, the specificity of DNA binding and conservation of specific amino acids or domains (except the bHLH domain), members of the bHLH superfamily, have been assigned into subfamilies, or subgroups, by different researchers. The bHLH transcription factors in *Arabidopsis*, poplar, rice, moss, and algal genomes have been divided into 32 subfamilies (Carretero-Paulet et al., 2010). Members of subfamilies 9 and 27 are essential for the growth and development of terrestrial plants, and subfamilies 7, 18, 19, and 20 are unique to angiosperms. Members of subfamily 5 regulate flavonoid/anthocyanin metabolism, epidermal cell development, and trichome initiation. According to the classification of subgroups, 166 bHLH genes of the *Arabidopsis thaliana* genome are divided into 13 major subgroups (I–XIII). Genes in a particular major group contain a similar number of introns at conserved positions, the encoded proteins have similar predicted lengths, and the bHLH domain is in a similar position in the protein. Genes within each major group can be further divided into a total of 26 subgroups (Heim et al., 2003; Pires and Dolan, 2010). The 95 bHLH genes in grape (Wang et al., 2018), 167 bHLH genes in rice (Li et al., 2006), and 152 bHLH genes in tomato (Wang et al., 2015) are divided into second-level subgroups by this method.

Anthocyanin biosynthesis is achieved by structural genes in the anthocyanin-biosynthesis pathway (Allan et al., 2008). At the transcriptional level, it is mainly regulated by a series of transcription factors, especially members of the R2R3–MYB gene family. In *Arabidopsis*, R2R3–MYB PAP1 and PAP2 regulate anthocyanin biosynthesis (Zimmermann et al., 2004; Gonzalez et al., 2008). In flavonoid biosynthesis, bHLH proteins serve as cofactors of R2R3–MYB, together with WD40, making up the MYB-bHLH-WD40 (MBW) complex (Hichri et al., 2011a; Xie et al., 2012; Wang et al., 2020). Most bHLHs that are involved in anthocyanin biosynthesis belong to subgroup III, which is functionally conserved and has been shown to regulate plant defense and development (Bailey et al., 2003; Heim et al., 2003). In *Arabidopsis*, subgroup III fbHLHs are involved in both flavonoid biosynthesis and trichome formation. The members share a conserved amino acid, arginine, which is involved in protein interactions and normal functions (Ludwig et al., 1989; Zhao et al., 2012).

Since the identification of bHLH transcription factor *Lc* (leaf color) in corn (Ludwig et al., 1989), TT8, GL3, and EGL3 of subgroup IIIf have been found to interact with TTG1 (WD40 protein family) and MYB (Bailey et al., 2003; Heim et al., 2003) to form protein complexes that regulate flavonoid biosynthesis. Most plants have at least two bHLHs belonging to two distinct clades within subgroup IIIf (Heim et al., 2003; Feller et al., 2011). They are described as bHLH-1 (represented by *ZmR/ZmLc, AtGL3, AtEGL3, AtMYC1, PhJAF13*, and *AmDel*) and bHLH-2 (represented by *ZmIn, AtTT8, PhAN1*, and *VvMYC1*) (Albert et al., 2014). The bHLH-2 genes are essential for anthocyanin biosynthesis. The overexpression of R2R3–MYB PAP1 resulted in elevated transcript levels of *TT8* in *Arabidopsis* (Gonzalez et al., 2008). In petunia, *PhAN2* requires the bHLH cofactor *PhAN1* or *PhJAF13* to enhance the promoter activity of dihydroflavonol 4-reductase (*DFR*) (Spelt et al., 2000). The co-expression of *VvMYC1* and *VvMYBA1* in grape suspension cells led to anthocyanin accumulation (Hichri et al., 2011a). Previous transcriptome analysis of fig has suggested that *FcMYB114, FcCPC, FcMYB21*, and *FcMYB123* regulate anthocyanin biosynthesis (Wang et al., 2019; Li et al., 2020a), but that no anthocyanin-related fig bHLH has been identified. In addition, genes from bHLH subgroup III d + e have been shown to regulate the jasmonic acid (JA) signaling pathway, thereby enhancing plant defense capabilities and promoting anthocyanin biosynthesis (Xie et al., 2012). Low temperature promoted the expression of *MdbHLH3*, which increased anthocyanin accumulation and fruit coloring in apple (Xie et al., 2012; Yang et al., 2017).

The fig (*Ficus carica* L.), which originated from the Mediterranean coastal region, is one of the earliest cultivated fruit trees in the world. The fig fruit (syconia) demonstrates a typical double sigmoid growth curve with a rapid growth phase, a lag phase, and another rapid growth phase (Flaishman et al., 2008). Ripe figs with dark color and red flesh have a high anthocyanin content, are beneficial to health, have a great market potential. In a previous study, we confirmed that the coloring of cv. Purple-Peel was due to anthocyanin accumulation (Wang et al., 2017). However, the bHLH transcription factors involved in fig anthocyanin accumulation have not been revealed. In this study, a total of 118 FcbHLH genes were recruited by searching the whole-genome database of fig; physical and chemical properties, phylogeny, chromosome distribution, conserved motifs, and protein interactions were analyzed bioinformatically, and *bHLH* expression patterns in the fig fruit at different development stages and under different treatment conditions were revealed. *FcbHLH42* was selected for a functional study that proved its role in fig anthocyanin synthesis. The results lay the foundation for understanding the role of FcbHLHs further in fig anthocyanin and flavonoid biosynthesis.

## Materials and Methods

### Plant Materials

The common fig cv. Purple-Peel from a commercial orchard in Weihai city, Shandong province, China (37°25′ N, 122°17′ E) was used. The fig trees were 7 years old with 3 m × 3 m spacing and standard cultivation. “Purple-Peel” is a bud mutation of “Green Peel,” a main fig cultivar in China (Wang et al., 2017). Six stages of the main crop fruit were sampled for gene-expression analysis based on the characteristics of fruit development. The fruit samples were marked as stages 1–6: stage 1 represented phase I (the first rapid growth period), stages 2, 3, and 4 were the early, middle, and late stages of phase II (slow growth period), and stages 5 and 6 represented phase III (the second rapid growth period). In this study, following Wang et al. (2019), stages 4 and 5 fruits were termed young and mature, respectively. Sixty fruits were randomly selected at each stage, and 20 were used as the biological replicate. The peel and female flower tissue were separated onsite at the time of sampling. Fresh samples were quick-frozen with liquid nitrogen and stored at −80°C for subsequent experiments.

### Identification and Annotation of FcbHLH Transcription Factors

The fig genome sequences of cvs. Horaishi and Dottato were downloaded from the National Center for Biotechnology Information (NCBI) (https://www.ncbi.nlm.nih.gov/genome/? Term=Ficus+carica) (Mori et al., 2017; Usai et al., 2020). The sequences were blasted (Evalue-5) using the hidden Markov model HMMER (v3.0) of Pfam (http://pfam.xfam.org/). Candidate genes containing the bHLH signature domain (PF00010) and with the most conserved amino acids in the bHLH region (Toledo-Ortiz et al., 2003) were screened further in the databases of Pfam, NCBI conserved domains (http://www.ncbi.nlm.nih.gov/Structure/cdd/wrpsb.cgi), and SMART (http://smart.emblheidelberg.de). Redundancies were removed. The bHLH family genes of *Arabidopsis thaliana* were downloaded from the Arabidopsis database (TAIR; https://www.arabidopsis.org/). The fig bHLH homologs were compared with *Arabidopsis* bHLHs by BLASTP with default parameters to obtain the annotation and grouping information. The FcbHLH and AtbHLH sequences were analyzed bioinformatically, and the physicochemical parameters of the proteins were calculated using ExPASy (http://www.expasy.ch/tools/pi_tool.html) (Guo et al., 2014).

### Phylogeny and Multiple-Sequence Alignment of FcbHLH Genes

ClustalX version 2.0 with default parameters was used to perform multiple-sequence alignments of the predicted bHLHs of fig and *Arabidopsis* (Larkin et al., 2007; Guo and Wang, 2017). A phylogenetic tree of the bHLHs was constructed with MEGA6.0, using the neighbor-joining (NJ) method with parameters set as follows: mode “p-distance,” gap setting “Complete Deletion,” and calibration test parameter “Bootstrap = 1000” (Tamura et al., 2011).

### Gene Structure and Protein Sequence Motif Analyses

The intron/exon structure map of the fig *bHLHs* was generated online using the Gene Structure Display Server (GSDS: http://gsds.gao-lab.org/). The conserved motifs were analyzed online using MEME4.11.2 (https://meme-suite.org/meme/tools/meme), with parameters set to: number of repetitions “any,” highest motif number “20,” motif length “6–200,” and default values for the other parameters. The results were constructed with TBtools (Chen et al., 2020).

### Chromosomal Location and Collinearity of bHLH Genes

The positions of *FcbHLHs* on the 13 fig chromosomes were determined by mapping bHLH gene sequences to fig chromosome survey sequences using BLAST programs. The Mapchart v2.2 software was used to display the precise gene-location results. The genome data of *Ficus hispida* and *Ficus microcarpa* were downloaded from the database of National Genomics Data Center (https://bigd.big.ac.cn/search/?dbId=gwh&q=PRJCA002187&page=1) (Zhang et al., 2020). The grape genome (*Vitis vinifera*) was also downloaded (https://data.jgi.doe.gov/refine-download/phytozome?organism=Vvinifera). An interspecies collinearity analysis of bHLHs between fig and *F. hispida, F. microcarpa, Arabidopsis* and grape was performed using MCscanX and TBtools (Tang et al., 2008; Chen et al., 2020). The final map was generated with Circos version 0.63 (http://circos.ca/). The non-synonymous replacement rate (Ka) and synonymous replacement rate (Ks) of the replicated gene pairs were calculated using KaKs_Calculator 2.0 (Wang et al., 2010), and environmental selection pressure was analyzed by Ka/Ks ratio.

### Functional Verification of bHLH Proteins

The interaction network of 118 FcbHLH proteins was analyzed using the STRING protein interaction database (http://string-db.org/), with *Arabidopsis* selected for species parameters. E-value was set to 1e-4.

Yeast strain Y2HGold (Clontech, San Francisco, CA, United States) was used for the yeast two-hybrid (Y2H) assay. Competent cells were co-transformed with the bait vector pGBKT7-FaMYB10 without self-activation, and the pGADT7 plasmid with possible interaction genes. Diploids carrying both plasmids were created by mating on yeast peptone dextrose (YPD) (1% yeast extract, 2% peptone, 2% glucose, and 2% agar) followed by selection on SD/-Trp/-Leu, SD/-Trp/-Leu/-His, or SD/-Trp/-Leu/-Ade plates. Single colonies growing on the SD/-Trp/-Leu plates were picked and individually cultured in a 1-ml yeast peptone dextrose medium with adenine (YPDA) (1% yeast extract, 2% peptone, 2% glucose,0.04% adenine, and 2% agar) liquid medium at 30°C for 2 days. A 1-μl aliquot of the yeast solution was pipetted on X-α-gal-containing SD/-His/-Leu/-Trp + AbA^*^and SD/-Ade/-His/-Leu/-Trp + AbA^*^ auxotrophic plates, and incubated at 30°C for 3 days. A single blue yeast colony indicated a positive interaction result.

### Gene-Expression Analysis

Three fig fruit RNA-seq libraries established by our laboratory were re-mined. The first library contained data of the “Purple-Peel” fig fruit during development (NCBI Accession No. PRJNA723733). Briefly, syconia peel and the internal female flower tissue were collected at six stages of fruit development. The second library contained data of young and ripe “Purple-Peel” and the peel of its mutated mother cv. Green Peel fruit (NCBI Accession No. SRP114533) (Wang et al., 2017). The third library contained data of bagged and naturally grown “Purple-Peel” fruit (NCBI Accession No. PRJNA494945) (Wang et al., 2019). TBtools was used to analyze the expression patterns of FcbHLHs in each library, and significant differential expression was determined by *p* < 0.05 and |log2(fold change) | ≥ 1.

A weighted gene co-expression network analysis (WGCNA) was performed to identify the modules of co-expressed genes (Langfelder and Horvath, 2008). Correlations of the co-expression relationships between *FcbHLH42* and other transcription factors were calculated according to their FPKM changes over the six stages of “Purple-Peel” fig development. The co-expression modules of *FcbHLH42* were visualized with Cytoscape 3.8.2. The thresholds for co-expression were set as correlation coefficient > 0.5 and *p* < 0.001.

The relative expression levels of *FcbHLH42*, strawberry (*Fa*)*MYB10, Nicotiana benthamiana* (*Nb*)*F3H, NbDFR, NbANS*, and *NbUFGT* in control and transient transgenic tobacco (*Nicotiana tabacum*) leaves were determined by quantitative reverse transcription (RT-q) PCR. The primer sequences are detailed in Supplementary Table 8. RNA extraction, DNA elimination, RNA quality check, and reverse transcription were carried out using the standard protocols of our laboratory (Wang et al., 2017). The RT-qPCR was carried out with ABI QuantStudio 6 Flex Real-Time PCR System (ABI, Waltham, MA, United Stated) using SYBR-Green Master Mix (Vazyme, Nanjing, China). The reaction program was: pre-denaturation at 94°C for 1 min, denaturation at 94°C for 15 s, annealing at 60°C for 30 s, and extension at 72°C for 1 min, for a total of 40 cycles. A relative quantification analysis with three replicates for each sample was performed as described in Zhai et al. (2021). Significance was analyzed with the SPSS 26.0 software.

### Cloning of *FcbHLH42* and Transient Expression

Strawberry *MYB10* was shown to act synergistically with sweet cherry *bHLH* to promote anthocyanin synthesis, but neither was able to promote anthocyanin synthesis alone (Wang et al., 2019). We took *FaMYB10* as bait to verify the function of FcbHLHs. *FaMYB10* was obtained from the strawberry cDNA library, and *FcbHLH42, FcbHLH3, FcMYC2*, and *FcbHLH14* from the “Purple-Peel” fig cDNA library. The primers are shown in Supplementary Table 8. *FcbHLH42* was transiently expressed using the HyperTrans vector system (Albert et al., 2021). The constructs were transformed into a *Agrobacterium tumefaciens* strain GV3101, and a fresh single colony was picked and cultured overnight at 28°C, and then centrifuged at 4,200 × g for 15 min. The bacteria were resuspended in a 15-ml agroinfiltration solution (10 mM MgCl_2_, 10 mM MES, pH 5.6) + 200 μM acetosyringone. *N. benthamiana* plants were grown in the greenhouse. The positive and negative controls, *FaMYB10*-expressing solution and *FcbHLH42*-expressing solution, respectively, were infiltrated into the back side of the leaves of 5-week-old tobacco (*N. tabacum*) plants (Sparkes et al., 2006). The leaves were photographed, and total anthocyanin content and gene expression were determined 7 days after the infiltration. Leaf tissue color was measured following Wang et al. (2019). Three biological replicates were used for the tests.

### Color Measurement

For treatment and controls, 1 g tobacco leaf tissue was collected and added to a 10-ml color extraction solution (methanol:water 1:1, pH 2). After ultrasonic extraction with oscillation at 200 rev/min at 25°C for 10 min, the sample was centrifuged at 10,000 × g at 4°C for 10 min, and the supernatant was collected. The leaf tissue was washed twice, and the supernatants were combined. After filtration through a 0.45-μm membrane, anthocyanin content was determined by differential pH method. Three biological replicates were used. Significance was analyzed with the SPSS 26.0 software.

## Results

### Genome-Wide Identification and Phylogenetic Analysis

**Figure 1:**
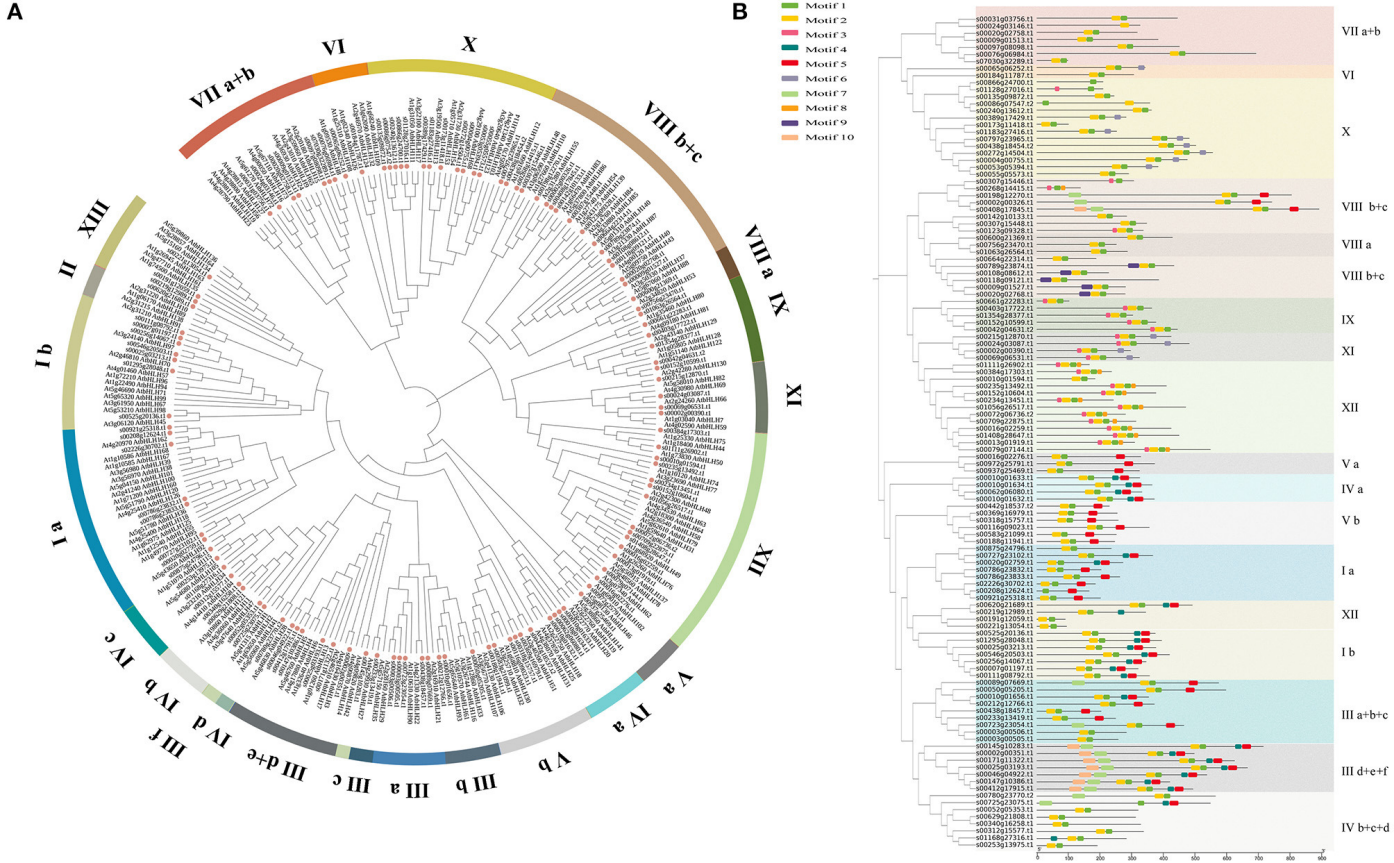
Phylogenetic tree and conserved motif analysis of FcbHLHs. **(A)** The 25 subgroups are marked in different colors on the periphery of the circle. Pink dots indicate fig bHLH proteins. The phylogenetic tree was constructed using MEGA6, with bootstrap values based on 1,000 iterations. **(B)** Rootless neighbor-joining (NJ) phylogenetic tree of 118 full-length amino acid sequences of FcbHLH proteins. The 25 subgroups are marked with different background colors. Conserved motifs are represented by different colored boxes.

A total of 118 bHLH genes were obtained from the published fig genome, and named *FcbHLH1* to *FcbHLH118* according to the equivalent classification for *Arabidopsis thaliana* (Figure 1A, and Supplementary Table 1). The predicted number of amino acids encoded by *FcbHLH*s ranged from 91 (*FcbHLH5*) to 892 (*FcbHLH56*), with an average of 356 amino acids per gene. The molecular masses of these proteins ranged from 10.25 (*FcbHLH5*) to 97.2 kD (*FcbHLH56*), and the isoelectric points were from 4.59 (*FcbHLH28*) to 11.53 (*FcbHLH111*), with 62.71% of them lower than 7, as predicted by ExPasy. This was similar to the isoelectric point pattern reported for the bHLH families of *Arabidopsis* (Bailey et al., 2003) and rice (Li et al., 2006). The hydrophilicity of the proteins ranged from −1.014 (*FcbHLH017*) to −0.014 (*FcbHLH053*), indicating that all FcbHLHs are hydrophilic. The instability index (II) ranged from 24.6 to 76.72, with only three indicated stable proteins (II < 40). The aliphatic index was between 50.08 and 102.86. Nuclear localization was predicted for most of the FcbHLHs, while cytoplasmic, chloroplastic, and mitochondrial matrix localization was predicted for a few of them. No signal peptide was found for any of the FcbHLHs by SignalP, demonstrating that they are non-secretory proteins.

To understand the evolutionary relationship of FcbHLH genes, a phylogenetic tree was constructed (Figure 1A). FcbHLHs were present in 25 of the 26 *Arabidopsis* bHLH subgroups; they were absent in subgroup II. Subgroups IX and X, both with 14 members, were the largest subgroups of FcbHLHs, while subgroups IIIf and IVd were the smallest, each with only a member. Fig and *Arabidopsis* had the same number of members in 12 subgroups, namely IIIa, IIIb, IIId + e, IVa, IVb, IVc, IVd, Va, Vb, VIIIb + c, IX, and XI. The biggest numerical difference was found in subgroup Ia, with FcbHLH members being less than half of their *Arabidopsis* counterparts. FcbHLH had more members than *Arabidopsis* in subgroups IIIa, IVb, VIIIa, and X.

### Sequence and Structure Analyses

Conserved motifs of FcbHLHs are shown in Figure 1B. Although the length of the FcbHLHs of different subfamilies varied greatly, the length and position of the conserved motifs were very similar. Motifs 1–10 are shown in different colors (Figure 1B), and the details of the 10 conserved motifs are shown in Table 1 and Supplementary Table 2. The number of introns in the *FcbHLH*s ranged from 0 to 19 (Supplementary Figure 1). In some subfamilies, the structural pattern of all members was similar. For example, members of subgroup VIIIb had no introns, whereas members of subgroup Ia had two introns, and the corresponding positions of the introns were conserved.

**Table 1:** Sequences of 10 predicted motifs of FcbHLH proteins.

**Motif**	**Best possible match**	***E*-value**	**Width**	**Sites**
1	K[TM]D[KT]AS[MI]L[DG][ED]A[IV]XY[VI]K[FE]LQRQ	2.0e-971	21	117
2	XSHSXAERRRRE[KR][IL][NS][ED]R[LFM]KAL [QR]SLVP[NG]XX	1.6e-1109	29	112
3	[KP]S[DV][YP][IC][HR]VRA[RK]RGQAT	1.6e-144	15	22
4	L[PA][DE][IV][ED]V[KRT][IV][VI][GDE]T[DEH][AV][LM][IL][RK][IV]Q[CS][PE][RK]	1.4e-72	21	23
5	[PQ]GLLLK[LI][MI]XAL[EQ]XL[GH]L[DE][VI][LV]HA[SN][IV][ST]T[FV][NG][GD]R	1.6e-126	29	39
6	C[DE][RS][AV][FK][EFL]A[RHQ][MS][HA]GIQT[LVI]VC[IV]P[MT][LP][ND]GV[VL]ELG[ST][TS]D[LS][IV]TE[DS][WL][SG]L[VL]Q	3e-60	41	8
7	[AP][KM]QDL[RQ]S[KR]GLCL[VM]P[IV]S[CL][TA]SA[VI]	7.4e-60	21	12
8	VEFLSMKL[AE][ATS][VA]N[PS]R[ML]	7.4e-54	15	13
9	[MIL][AG][AQ]M[KR]EM[IM][YF][RGK][IA]A[AV][MF][QR]P[VI][DHN][IL][DG][PL][EA][STI][ITV][KEPR][KPR]P[KR]R[RK]NV[RK]IS[DKT]	1.1e-48	36	5
10	[DE][DI][LV]TD[IT][ED]WF[YF][LT][MV]S[VM]T[RF][ST]F[PS]A[GE]SG[AL]PG[KR][AS]YSSGA[HY]VW[LV][TS]G[AN]	1.4e-46	40	6

The promoter region was obtained by searching the 2-kb sequence upstream of the translation initiation site of *FcbHLH*s from the fig genome (Mori et al., 2017; Usai et al., 2020). At least 16 *cis*-regulatory elements were predicted (Supplementary Figure 1). The elements participated in responses to abiotic stresses (light deprivation, drought, low temperatures, anaerobic conditions, defense, and stress), hormone responses (salicylic acid, gibberellin, methyl jasmonate, abscisic acid, and auxin), circadian rhythm regulation, and nutrition and development (meristem expression and endosperm expression) in all the FcbHLH genes. Light response and anaerobic-induced abiotic stress response regulatory elements, and MYB-binding sites were found in five *bHLH* (*FcbHLH8, FcbHLH24, FcbHLH83, FcbHLH46*, and *FcbHLH21*) promoters.

### Expansion Patterns and Collinear Correlation

**Figure 2:**
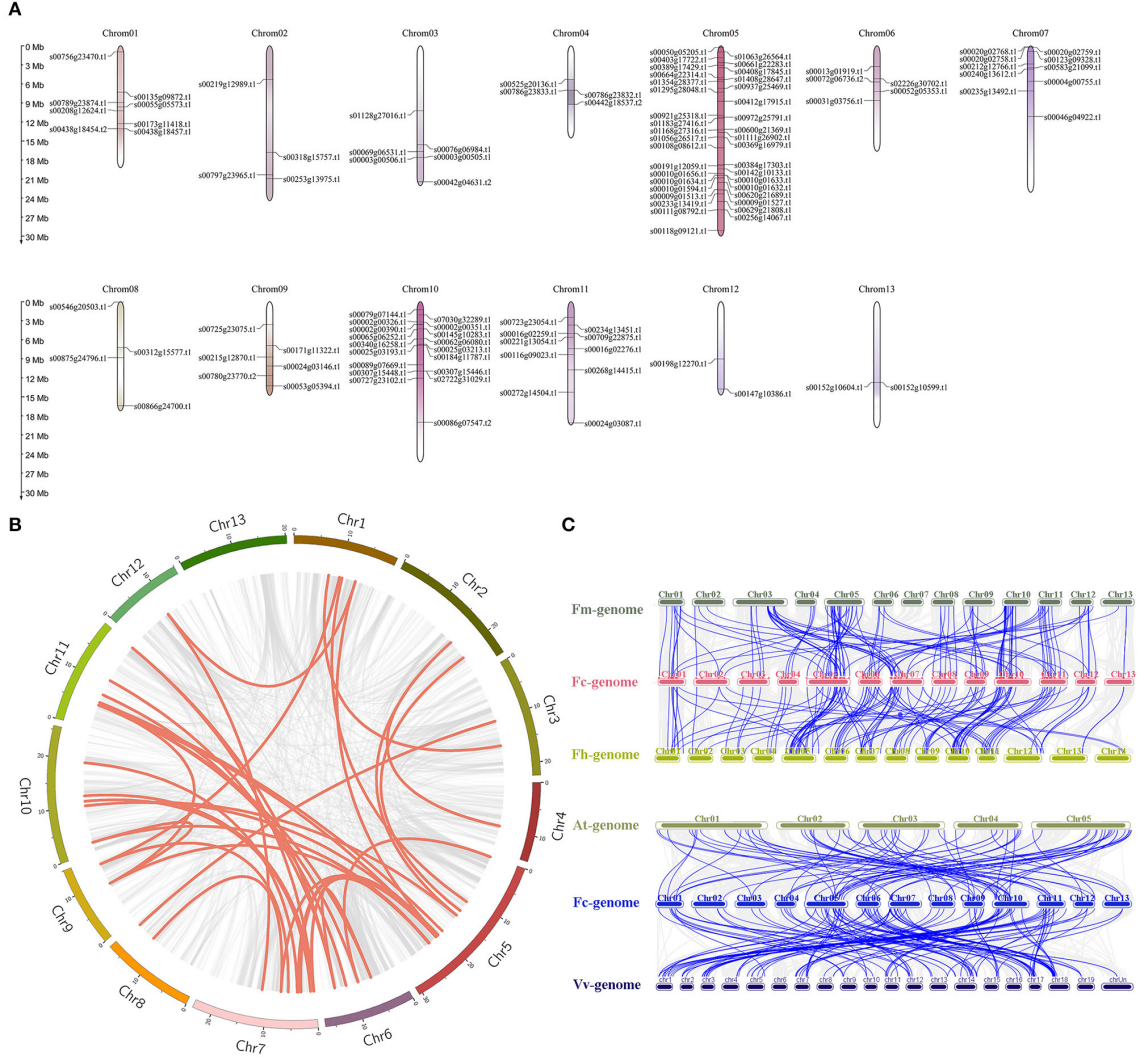
Chromosomal locations and collinearity analysis of the FcbHLH gene family. **(A)** FcbHLH genes are marked on chromosomes; scale bar on the left indicates length of fig chromosome (Mb). **(B)** Collinearity analysis of FcbHLH genes; circle plot was created with the MCScanX tool. Identified collinear genes are linked by colored lines. **(C)** Collinearity relationship of bHLH genes among *Ficus carica* (Fc), *Ficus hispida* (Fh), *Ficus microcarpa* (Fm), *Arabidopsis thaliana* (At), and *Vitis vinifera* (Vv). Identified collinear genes are linked by blue lines.

Large-fragment chromosome replication and tandem repeat are key means of gene family expansion. In our study, the 118 *FcbHLH*s were unevenly distributed among the chromosomes, with a maximum of 23 on chromosome 5 and a minimum of 3 on chromosome 13 (Figure 2A). It is generally believed that tandem replication occurs when the distance between genes is <100 kb, and 15 pairs of *FcbHLH*s were in that range (Supplementary Table 3). An intraspecific collinearity analysis showed that 11 pairs of *FcbHLH*s originated from fragment replication (Figure 2B and Supplementary Table 4). The results demonstrated that tandem replication and fragment replication were important events in the expansion of the FcbHLH gene family. The Ka/Ks values of all homologous FcbHLH gene pairs were <1 (Supplementary Table 5), suggesting negative selection.

The collinearity analysis identified 121 orthologs between *F. carica* and *F. hispida*, and 119 orthologs between *F. carica* and *F. microcarpa*, suggesting similar evolutionary distances between edible fig and the two evergreen *Ficus* species. There were two and seven isozymes between fig and *F. hispida* or *F. microcarpa*, respectively, indicating that evolution between fig and *F. hispida* tended to involve gene replication. Seventeen FcbHLH genes were not found to have a collinear relationship with either of the two other *Ficus* species, indicating possible unique bHLHs in the evolution of fig. A total 73 and 126 orthologous gene pairs were identified between fig and *Arabidopsis*, and between fig and grape, respectively (Figure 2C), indicating a closer homologous evolutionary relationship of the fig bHLH gene family with grape than with *Arabidopsis*. *FcbHLH40* (s00118g09121.t1) and *FcbHLH54* (s00307g15448.t1) only showed a collinear relationship with the two other *Ficus* species; both belonged to the VIIIb + c subgroup, indicating that this subgroup was relatively conserved in the evolution of *Ficus* plants. Detailed results of the analysis are shown in Supplementary Table 6.

### Expression Pattern of *FcbHLH*s in Fruit

**Figure 3:**
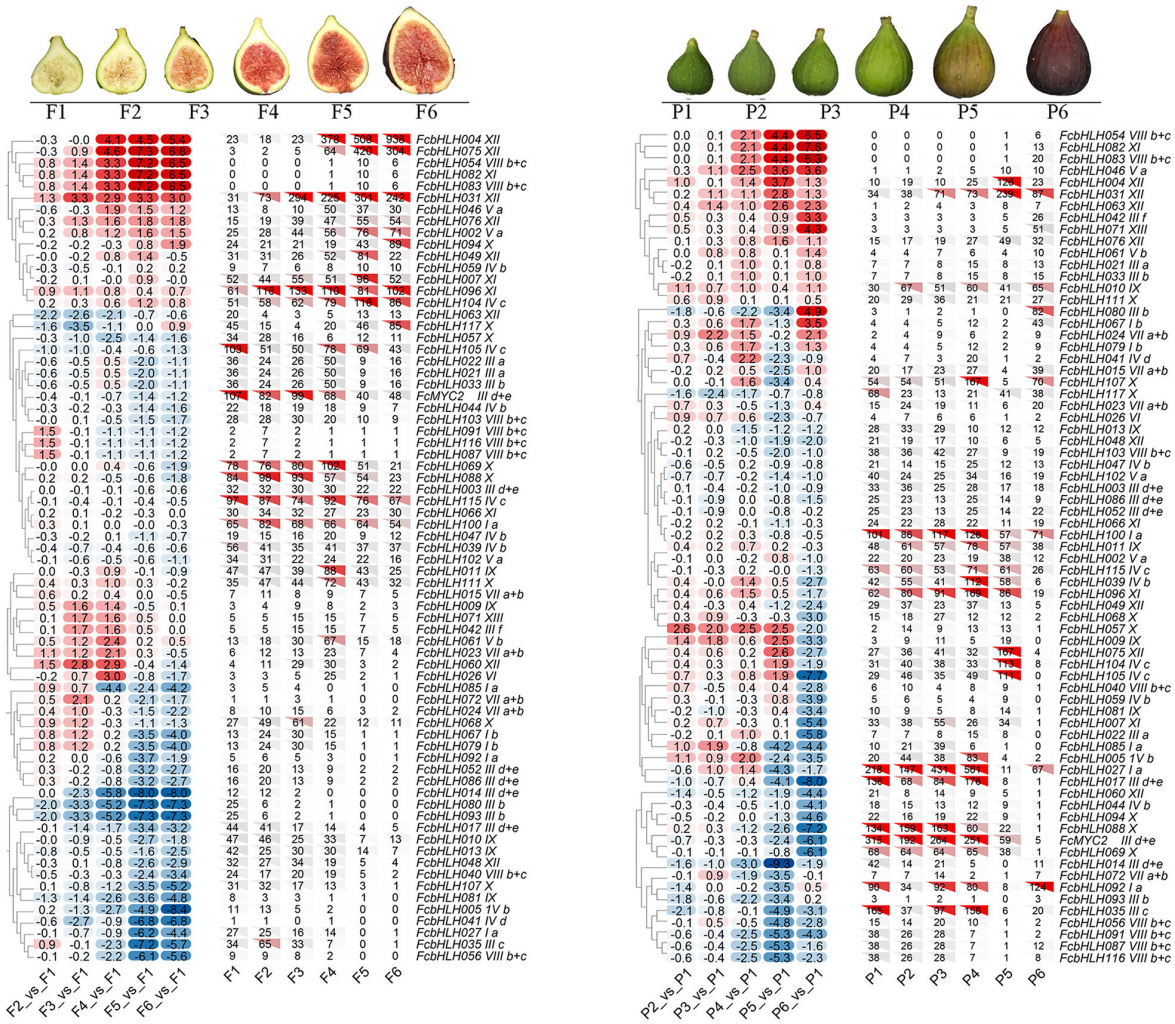
Expression profile of FcbHLH genes in the female flower tissue and peel of fig fruit. Expression of FcbHLH genes is expressed by FPKM value and log_2_FC. Hierarchical clustering method and average linkage method were used to construct the clustering tree. Log_2_FC > 0 indicates upregulation, and log_2_FC < 0 indicates downregulation. F1–F6 and P1–P6 represent the six stages of “Purple-Peel” female flower tissue and peel, respectively, during fig fruit development.

Among the 118 FcbHLH genes, 72 were at FPKM > 10 in at least a sample of the peel and female flower tissue at different stages of fig fruit development (Figure 3). Fifteen *FcbHLH*s from eight subfamilies demonstrated FPKM > 100, with subgroup XII leading the list with three members (*FcbHLH4, FcbHLH31*, and *FcbHLH75*). All three of these genes were upregulated along female flower development; in the peel they continued to increase until the fruit started ripening, at which point their expression decreased. Members of the same subgroup could have different expression patterns: in subgroup VIIIb + c; for example, *FcbHLH54* and *FcbHLH83* were expressed in the late stage of female flower and peel development, whereas *FcbHLH56, FcbHLH91, FcbHLH81*, and *FcbHLH116* were specifically expressed in the early stage of peel development, and *FcbHLH40* was highly expressed in the early and middle stages of female flower development. Members of subfamily IIId + e, such as *FcMYC2, FcbHLH96, FcbHLH31*, and *FcbHLH4*, which are closely related to the JA signaling pathway, were clearly repressed during female flower and peel development.

**Figure 4:**
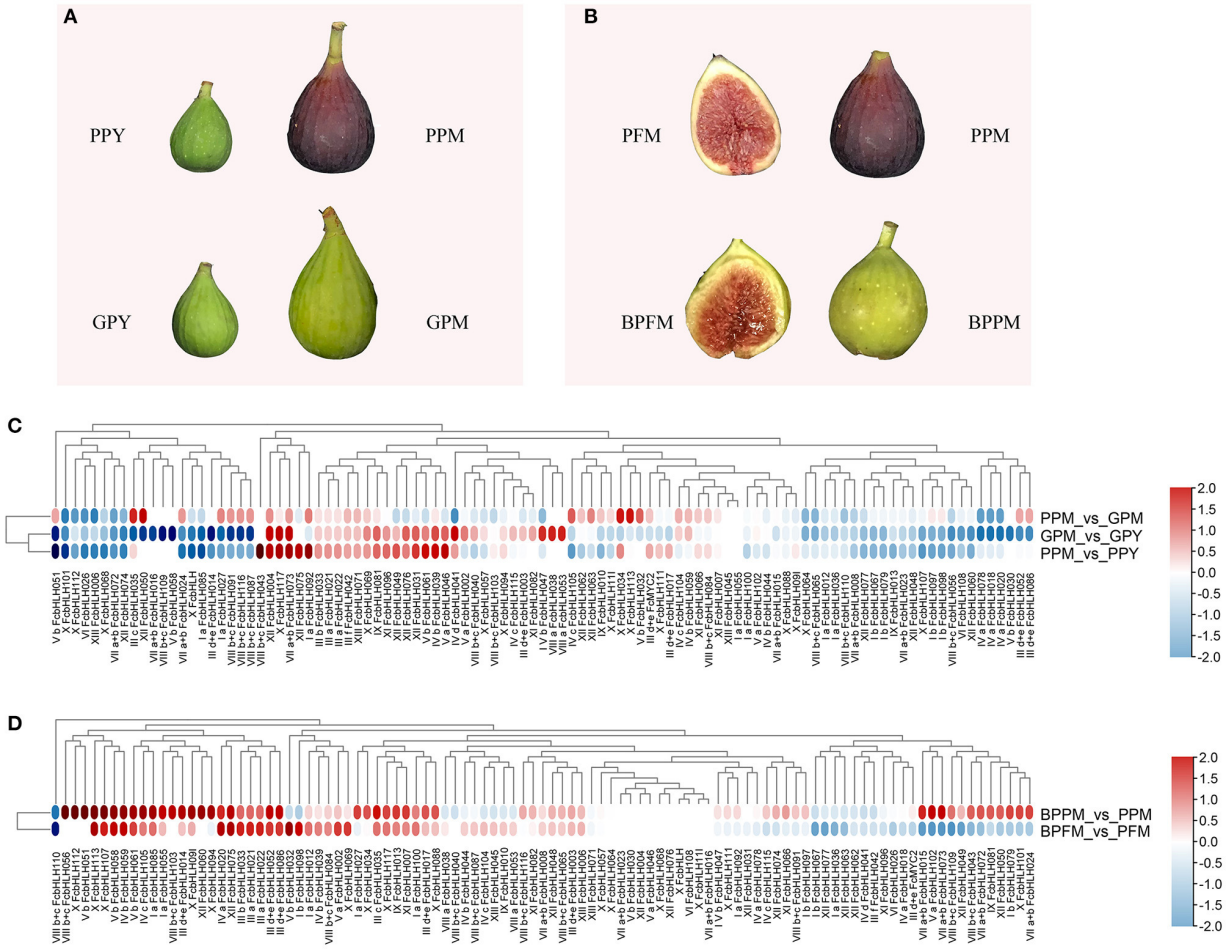
Expression profiles of FcbHLH genes in different fig cultivars and under fruit-bagging treatment conditions. Log_2_FC > 0 indicates upregulation, and log_2_FC < 0 indicates downregulation. PPY and GPY represent the young-stage fruit peel of “Purple-Peel” and “Green Peel” cultivars, respectively; PPM and GPM represent the ripening-stage fruit peel of “Purple-Peel” and “Green Peel,” respectively; PFM represents the ripening-stage female flower of “Purple-Peel”; BPFM and BPPM represent the ripening female flower and peel of “Purple-Peel” fruit, respectively, after bagging. **(A)** peel color of two fig cultivars. **(B)** the effect of fruit bagging on female flower and peel color. **(C)** expression comparison of FcbHLH genes. **(D)**, the effect of fruit bagging on the expression of FcbHLH genes.

“Purple-Peel” and “Green Peel” are a pair of bud mutant cultivars with a different peel color at fruit ripening (Figure 4A). The expression pattern of *FcbHLH*s during fruit development was consistent in the two cultivars: 64 members showed the same expression trend, with 20 upregulated and 44 downregulated *FcbHLH*s during fruit development (Figure 4B). In the peel of the ripe fruit, 51 *FcbHLH*s were highly expressed in “Green Peel,” but only 35 members were highly expressed in “Purple-Peel.” *FcbHLH17* of subgroup IIId + e and *FcbHLH35* of subgroup IIIc were upregulated in “Purple-Peel” and downregulated in “Green Peel” during fruit ripening, with the expression level of *FcbHLH35* in ripening-stage “Purple-Peel” fruit peel being significantly higher than that in its “Green Peel” counterpart (2.11-fold). The differential expression of *FcbHLH* family members in this pair of cultivars supported their different secondary metabolite contents (Wang et al., 2017).

Anthocyanin synthesis in fig peel is light-dependent, whereas in the female flower tissue it is not (Figure 4C). For the bagged fruit, 68 and 22 *FcbHLH*s demonstrated upregulation and downregulation in the peel of the mature fruit, respectively, of which *FcbHLH98* of subgroup Ib was downregulated by 1.24-fold and showed an increment in the female flower. *FcbHLH42* of subgroup IIIf was downregulated by 0.82-fold after bagging and might be involved in the light-dependent anthocyanin-synthesis pathway in the peel (Figure 4D).

### Interaction Network Prediction of bHLHs Involved in Anthocyanin Biosynthesis

**Figure 5:**
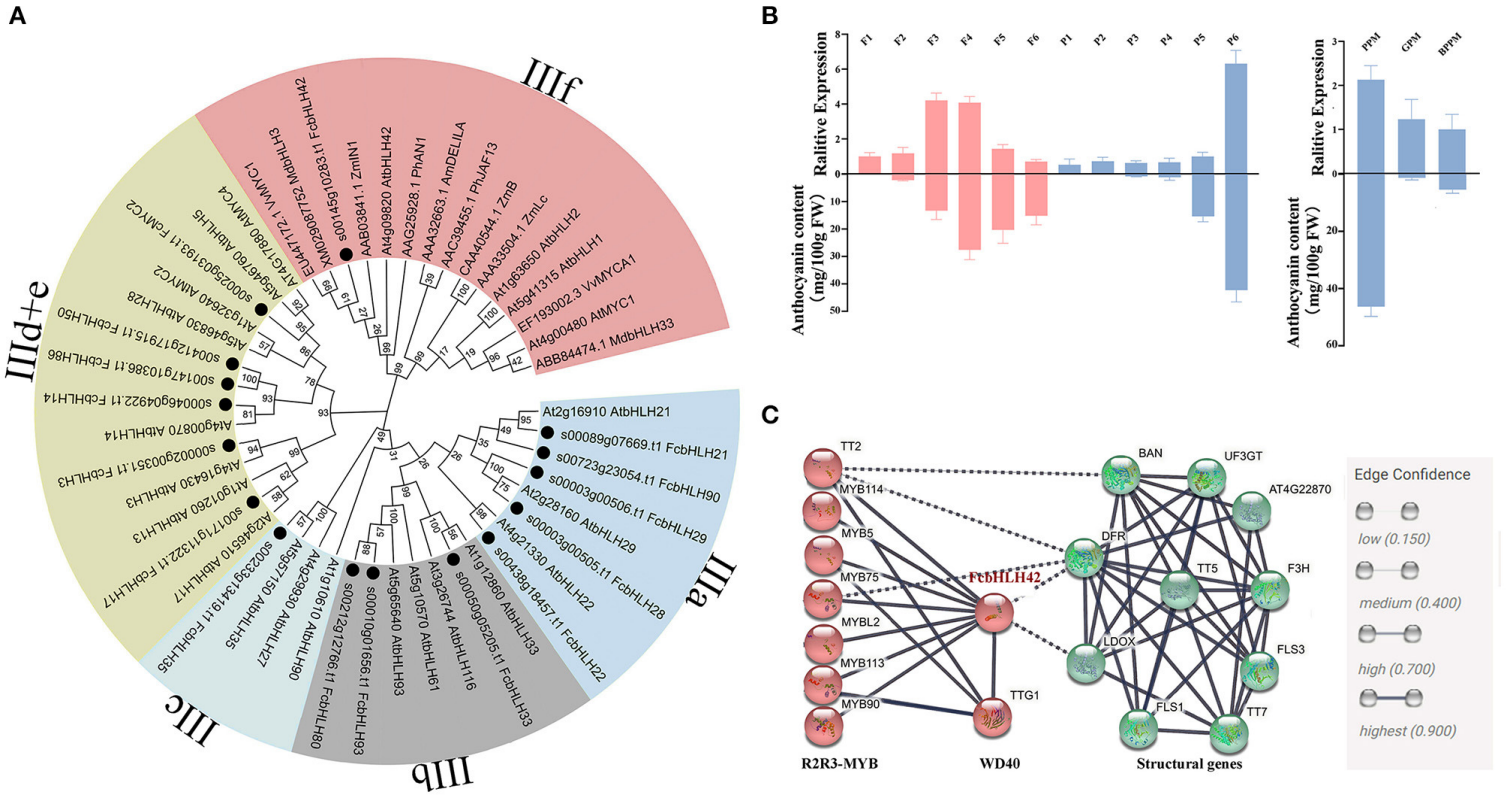
Subgroup III FcbHLHs: phylogenetic tree analysis, expression profile, and interaction network of FcbHLH42. **(A)** NJ phylogenetic analysis of subgroup III FcbHLHs, AtbHLHs and bHLHs of other species. Different colors indicate different subgroups, and black dots indicate FcbHLHs. **(B)** Expression heat maps of 16 FcbHLH genes of subgroup III. After log_2_ conversion, log_2_FC > 0 indicates upregulation, and log_2_FC < 0 indicates downregulation. F1–F6 and P1–P6 represent the six stages of female flower and fruit peel development, respectively. GPY and GPM, peel of young and mature “Green-Peel” fruits, respectively. PPY and PPM, peel of young and mature “Purple-Peel” fruits, respectively. PFM, female flower of mature “Purple-Peel” fruit. BPPM and BPFM, peel and female flower of mature “Purple-Peel” fruits that were bagged at the young stage. **(C)** Interaction network of FcbHLH42 from the perspective of *Arabidopsis thaliana* homologous genes. Red represents transcription factors, and green represents key synthase genes.

Among the 118 *FcbHLH*s, 16 were assigned to subfamily III and their phylogenetic distance is shown in Figure 5A. Among the 16 genes, only *FcbHLH42*, encoding a bHLH-2 protein, was clustered in the IIIf subgroup, which also included *ZmLC1, AtbHLH42* (TT8), *AtMYC1*, and other bHLHs that have been confirmed to be related to anthocyanin synthesis. *FcbHLH42* was most closely related to apple (*Md*)*bHLH3* and *VvMYC1*.

*FcbHLH42* expression was upregulated by 1.68- and 1.62-fold at stages F3 and F4, when female flower color is developing, and upregulated by 1.99-fold at stage P6 of peel coloring (Figure 3). “Purple-Peel” fruit bagging led to the 1.06-fold repression of *FcbHLH42* compared with the control fruit. A relative expression analysis showed high synchronicity between *FcbHLH42* expression and the corresponding anthocyanin content in the female flower and peel of fig fruit. Moreover, in the peel of the ripening fruit, *FcbHLH42* was repressed in both “Purple-Peel” and “Green Peel” after bagging (Figure 5B).

The co-expression analysis is shown in Supplementary Figure 4. During the development of “Purple-Peel,” MYBs (*FcCPC, FcMYB114, FcMYB5-1, FcMYB5-2*, etc.), WD40, and anthocyanin synthesis structural genes (*FcCHS, FcCHI, FcANS, FcUFGT1*, etc.), were strongly positively correlated with the expression of *FcbHLH42*. Specific co-expression combinations are listed in Supplementary Table 9.

Proteins with predicted interaction scores higher than 0.7 with FcbHLH42 are shown in Figure 5C. The red circles are R2R3–MYB genes, i.e., *TT1* (*MYB75*), *TT2, MYB5, MYB113, MYB114, MYB90, MYBL2*, and *TTG1* (*WD40*). The green circles represent key enzymes in the anthocyanin biosynthesis pathway, predicting that FcbHLH42 may be involved in the regulation and expression of DFR and LDOX (ANS).

*FcbHLH3, FcMYC2*, and *FcbHLH14* are clustered in subgroup IIId + e (Figure 5A). MYC2 is the core element of the COI1–JAZ–MYC2 complex that serves an important role in the JA signaling pathway in plants. The bHLHs in the IIId + e subgroup conservatively participate in the regulation of genes related to stress response and JA signaling. In our transcriptome data, *FcMYC2* was upregulated by 1.8-fold in the late-stage peel of the naturally grown fruit, whereas it was downregulated in the bagged fruit.

### *FcbHLH42* Promotes Anthocyanin Accumulation in Transgenic Tobacco by Interaction With MYB

**Figure 6:**
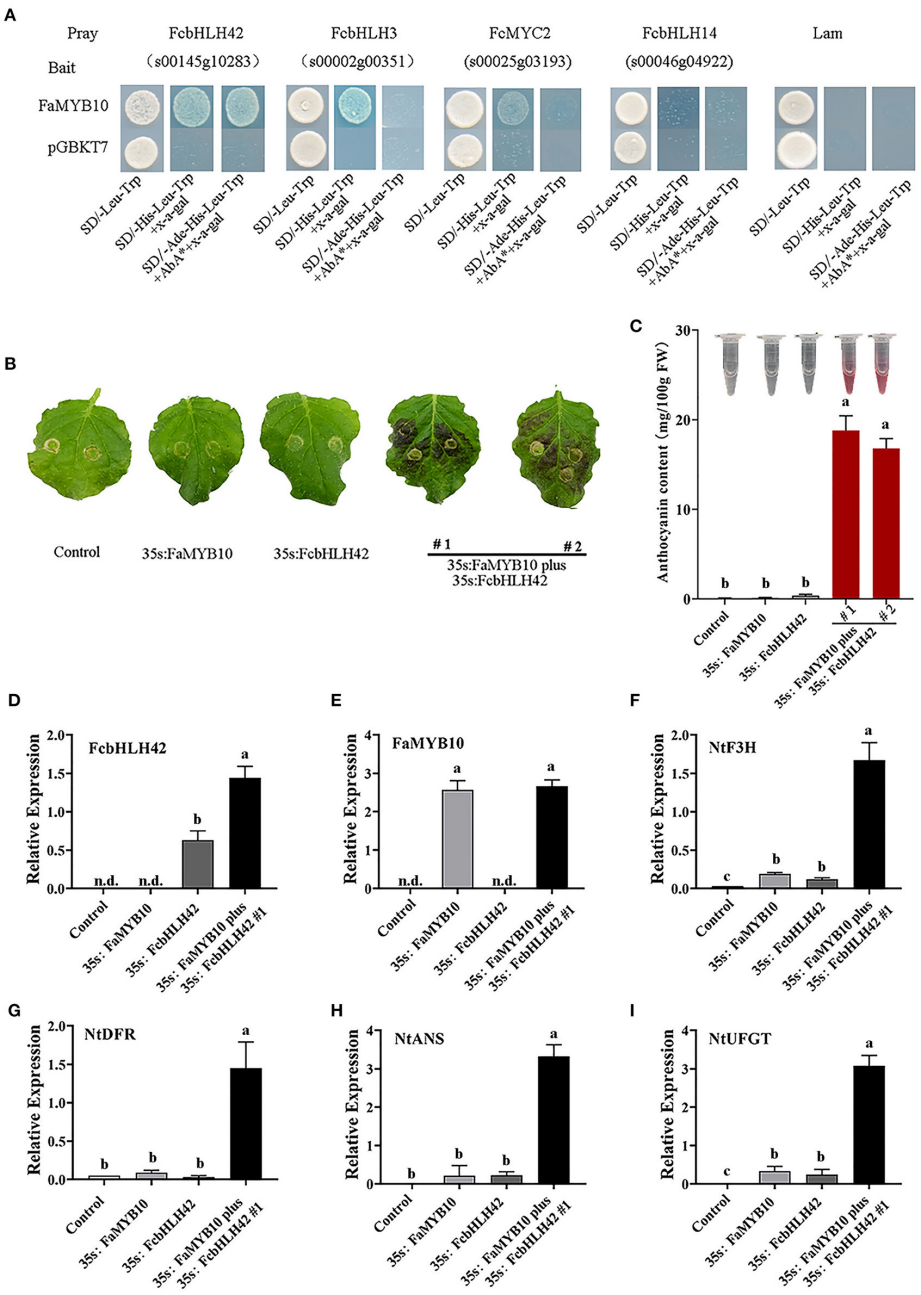
Analysis of FcbHLH interactions with MYB by Y2H and transient overexpression of *FcbHLH42*. **(A)** Y2H tests for four FcbHLHs and FaMYB10 (R2R3–MYB). Interactions of FcbHLH42 (s00145g10283), FcbHLH3 (s00002g00351), FcMYC2 (s00023g03193), FcbHLH14 (s00046g04922), and FaMYB10. **(B)** Phenotype of tobacco leaves after infiltration. Tobacco (*N. benthamiana*) leaves were infiltrated with cDNA constructs corresponding to *FcbHLH42, FaMYB10*, and agroinfiltration solution (control). 35S: *FaMYB10* plus 35S: *FcbHLH42* #1 and #2 are two individual plants. **(C)** Color display after total anthocyanin extraction from the infiltrated tobacco leaves and the anthocyanin content in tobacco leaves after infiltration. **(D–I)** Expression of *FcbHLH, FaMYB10, NbF3H, NbDFR, NbANS*, and *NbUFGT* in the control, 35S:*FaMYB10*, 35S:*FcbHLH42*, and 35S:*FaMYB10* plus 35S: *FcbHLH42* #1 tobacco leaves after infiltration; n.d., not detected.

A positive interaction between FaMYB10 andFcbHLH42 is shown by Y2H, along with weak interactions of FaMYB10 with FcbHLH3 and FcMYC2 (Figure 6A).The role of *FcbHLH42* in anthocyanin biosynthesis was analyzed further with transient transgenic technology using tobacco leaves. Leaves with combined overexpression of *FcbHLH42* and *FaMYB10* were purple, whereas those with the control *Agrobacterium* line-containing vector, or a single injection of *FaMYB10* or *FcbHLH42*, were not (Figure 6B). The anthocyanin content following *FcbHLH42* + *FaMYB10* overexpression was also significantly higher than that of the control and the single transcription factor injections (Figure 6C). The transcription levels of four genes related to anthocyanin synthesis, i.e., *NbF3H, NbDFR, NbANS*, and *NbUFGT*, were all significantly increased in the *FcbHLH42* + *FaMYB10* combination. In the control group without color change, the expression levels of the four genes are low to undetectable (Figures 6D–I).

## Discussion

### Gene Structure Provides Information on *FcbHLH* Evolutionary Relationships

The bHLH transcription factor family is the second largest family in plants and participates in various regulatory metabolic activities. Following the taxonomy of the *Arabidopsis* bHLH family, 118 FcbHLH genes were divided into 25 subgroups in this study, with most members in a particular subgroup bearing the same intron pattern and conserved motifs, suggesting the regulation of similar biofunctions (Figure 1 and Supplementary Table 1).

The phylogenetic topology diagram revealed 10 highly conserved amino acid motifs in the 118 FcbHLHs. Signature Motifs 1 and 2 were found in almost all FcbHLH proteins and were always adjacent to each other, constituting the bHLH domain (Figure 1B). Most of the conserved motifs in a particular subgroup were similar, supporting the evolutionary classification of the FcbHLH gene family. The uniqueness and conservation of motifs in each subgroup indicate that the functions of the encoded bHLHs in that subgroup are stable, and that the specific motifs are pivotal in the implementation of the corresponding regulatory function.

The expansion of a gene family is mainly driven by gene duplication and subsequent diversification; tandem repeats and large-fragment replication are two major means of gene expansion (Vision et al., 2000). Tandem repeats refer to two adjacent genes on the same chromosome, and large-fragment replication events involve different chromosomes (McGowan et al., 2020). Chromosome localization indicates that FcbHLH genes are unevenly distributed (Figure 2A). It was speculated that 29 of the 118 FcbHLH genes had tandem repeat events, similar to the ratio reported for the potato (20 out of 124) (Wang et al., 2020) and tomato (14 out of 159) (Sun et al., 2015) bHLH families. The sequence of tandem replications was very similar in the conserved region, and their genetic relationship in the evolutionary tree was also very close. As a result, similar functions are expected.

### bHLHs Expressed in Fig Fruit

The bHLH family plays a number of important regulatory roles in fruit-related growth and development, such as carpel, anther, and epidermal cell development, phytochrome signaling, flavonoid biosynthesis, and hormone signaling (Feller et al., 2011; Vanstraelen and Benkova, 2012). Our study revealed a universal expression of a large number of FcbHLHs in the fig fruit. Seventy-one *FcbHLH*s were transcribed in both female flower and peel, while 9 and 7 *FcbHLH*s demonstrated a peel- and female flower tissue-specific expression, respectively, suggesting important roles for bHLH family members in fig fruit development.

Among the 159 tomato bHLH genes, 11 displayed a tendency toward fruit-specific expression defined by >2-fold expression in fruit compared with other tissues. The bHLHs further showed a divergent expression during fruit development and ripening, and ethylene-responsive elements were found with the promoter of 7 bHLH genes (Sun et al., 2015). Three highly expressed *bHLH*s in the fig fruit, *FcbHLH4, FcbHLH31*, and *FcbHLH75*, all belonging to subgroup XII, were upregulated at fruit ripening. Subgroup XII has been shown to regulate brassinosteroid signaling, flower initiation, and cell elongation in other plants (Niu et al., 2017). The role of the highly expressed *FcbHLH*s needs to be further elucidated.

Basic helix–loop–helixes that have been suggested to regulate anthocyanin and proanthocyanin biosynthesis are often nominated according to their expression pattern and phylogenetic clustering. In the positively correlated co-expression network of *FcbHLH42* in subgroup IIIf, there were key structural genes for anthocyanin synthesis (*FcANS, FcUFGT*, etc.), and fig MYBs (*FcMYB114, FcMYB5*, etc.). The expression of grape *VvMYC1* has been reported to be correlated with the synthesis of anthocyanins and proanthocyanins in skin and seeds during berry development, suggesting that *VvMYC1* is involved in the regulation of anthocyanin and proanthocyanin synthesis in grapes. Similarly, the transient expression of *VvMYC1* and *VvMYBA1* induced anthocyanin synthesis in grapevine suspension cells (Hichri et al., 2010, 2011b). In blueberry, seven bHLH genes had differential expression patterns during fruit development (Zhao et al., 2019). Three jujube candidate bHLH genes, *ZjGL3a, ZjGL3b*, and *ZjTT8*, were suggested to be involved in anthocyanin biosynthesis and classified into subgroup III (Shi et al., 2019). Functional validation is required to confirm the specific role of these bHLHs.

### *FcbHLH* Involvement in Fig Fruit Anthocyanin Biosynthesis

Only a few bHLH transcription factor genes, such as *VvMYC1, FvbHLH9, MdbHLH3*, and *MdbHLH33*, have been identified as being associated with anthocyanin biosynthesis in fleshy fruit (Espley et al., 2007; Hichri et al., 2011a; Li et al., 2020b). Our study revealed the first *bHLH* involved in fig fruit anthocyanin biosynthesis.

Previous studies have shown that bHLH genes of subgroup IIIf interact directly with anthocyanin biosynthesis. Previous reports have elucidated two functionally redundant *bHLH*s, *AmInc I* and *AmDel*, which directly regulate anthocyanin biosynthesis in *Antirrhinum majus* (Albert et al., 2021). In apple, *MdbHLH3* and *MdbHLH33* have been characterized in relation to anthocyanin biosynthesis (Xie et al., 2012). In figs, only *FcbHLH42* was assigned to subgroup IIIf. The selection of FcbHLH42 for a further study on its involvement in anthocyanin synthesis was also supported by its homologous clustering with confirmed anthocyanin biosynthesis-regulating *VvMYC1* and *MdbHLH3*, and the positive results from protein interaction and co-expression analyses. Although the function of *FcbHLH42* was confirmed by a series of experiments, including transient expression, in this study, further investigation could reveal other FcbHLHs that regulate anthocyanin biosynthesis in the fig fruit.

The FPKM values of *FcbHLH42* were 15 and 26 in the female flower tissue and peel during the stage of rapid anthocyanin content increase, but were not very high compared with those of the highly expressed FcbHLHs, or the FPKM values of the color development-regulating FcMYBs. *FcbHLH42* is a bHLH-2 gene. In addition to bHLH-2, bHLH-1 proteins could act in controlling anthocyanin biosynthesis (Zhang and Hulskamp, 2019). bHLH-1 and bHLH-2 transcription factors are suggested to function *via* distinct mechanisms (Pesch et al., 2015; Zhang et al., 2019). A previous transcriptome study has revealed that *FcMYB114, FcCPC, FcMYB21*, and *FcMYB123* might regulate anthocyanin biosynthesis in the fig fruit (Wang et al., 2019; Li et al., 2020a). WD40 and other MYB transcription factors are shown to be positively correlated with *FcbHLH42* in this study, which provides the basis for better analyses of various regulatory models of anthocyanin synthesis in figs. Moreover, our experiments showed that *FcbHLH3, FcMYC2*, and *FcbHLH14* are closely related to JAZ family members and have a predicted interaction with TT2/TTG1/MYB75 (Supplementary Figure 3 and Supplementary Table 7). Their role in anthocyanin synthesis warrants a further study.

## Data Availability Statement

The datasets presented in this study can be found in online repositories. The names of the repository/repositories and accession number(s) can be found in the article/Supplementary Material.

## Author Contributions

MS and HM designed the experiments. MS and HW conducted the experiments. MS, HW, ZW, and HH analyzed the results and prepared the manuscript. HM and SC revised the manuscript. All authors read and approved the final manuscript.

## Funding

This study was supported by the National Natural Science Foundation of China [31372007].

## Conflict of Interest

The authors declare that the research was conducted in the absence of any commercial or financial relationships that could be construed as a potential conflict of interest.

## Publisher's Note

All claims expressed in this article are solely those of the authors and do not necessarily represent those of their affiliated organizations, or those of the publisher, the editors and the reviewers. Any product that may be evaluated in this article, or claim that may be made by its manufacturer, is not guaranteed or endorsed by the publisher.
