# Supplementary figures and images for: Genome-Wide Characterization and Analysis of bHLH Transcription Factors Related to Anthocyanin Biosynthesis in Fig (Ficus carica L.)
Source: Front Plant Sci. 2021 Oct 8;12:730692. doi: 10.3389/fpls.2021.730692 (PMC8531510; doi:10.3389/fpls.2021.730692)

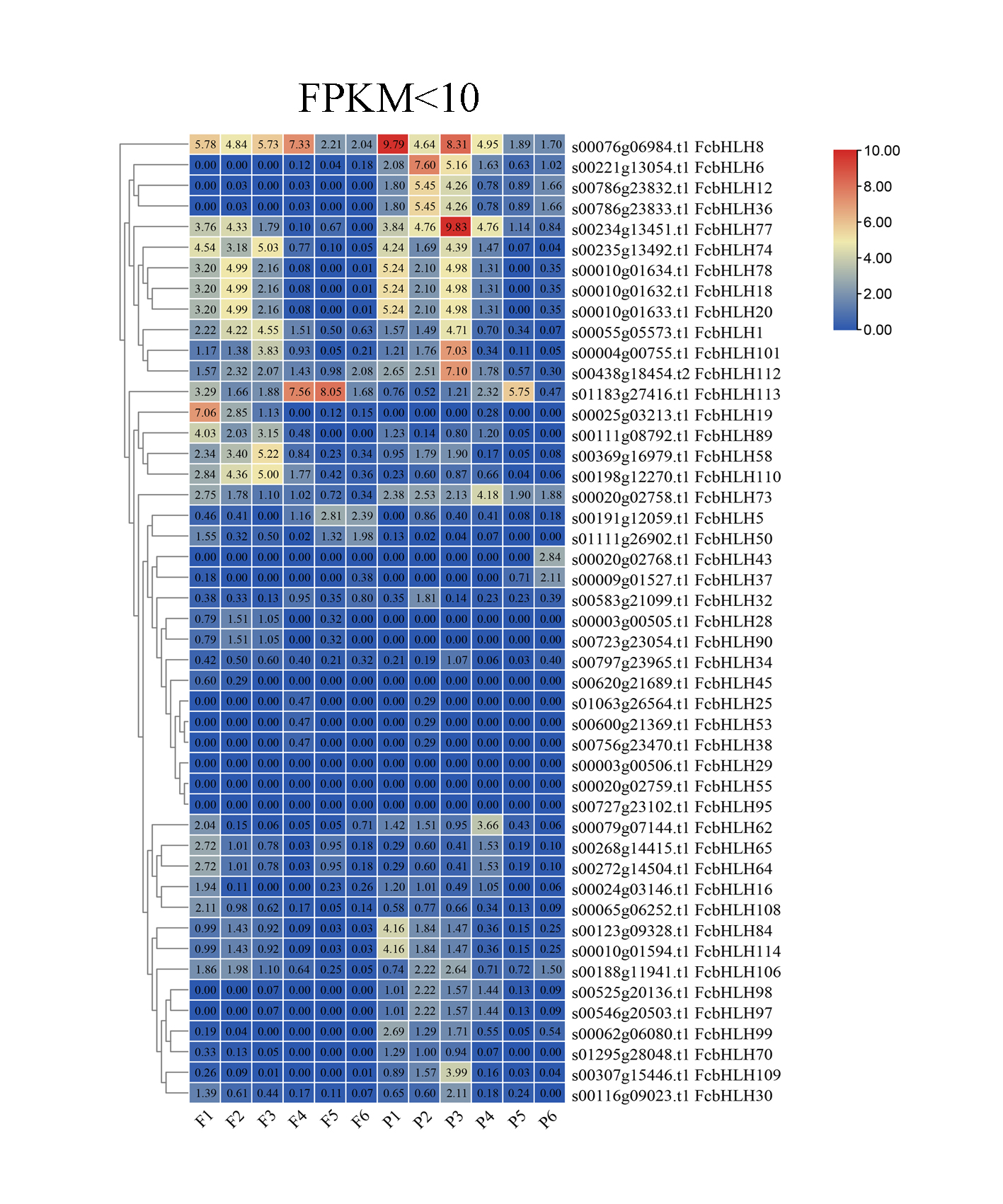

Supplement: Supplementary file 1 [file Image_1.JPEG]

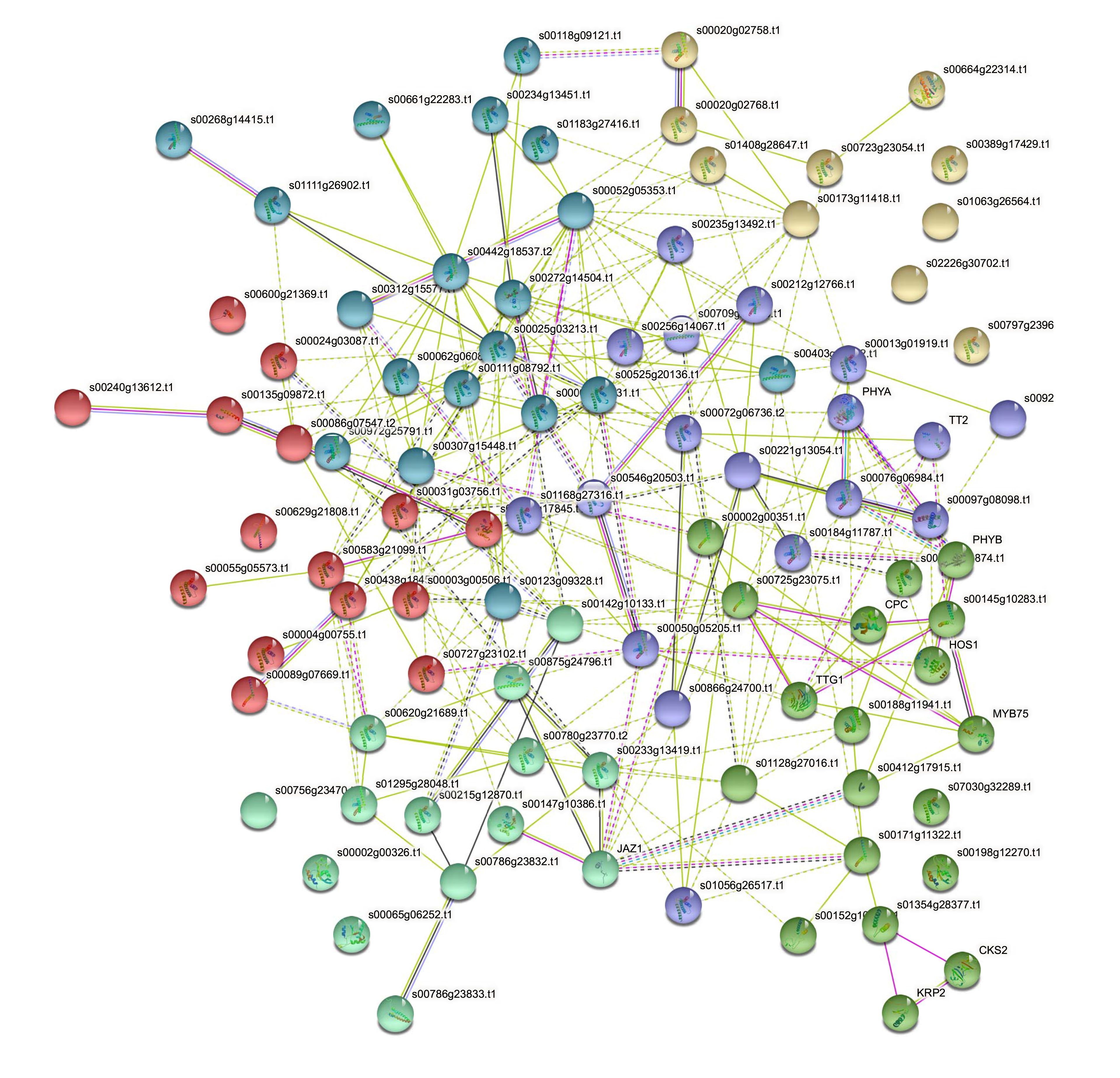

Supplement: Supplementary file 2 [file Image_2.JPEG]

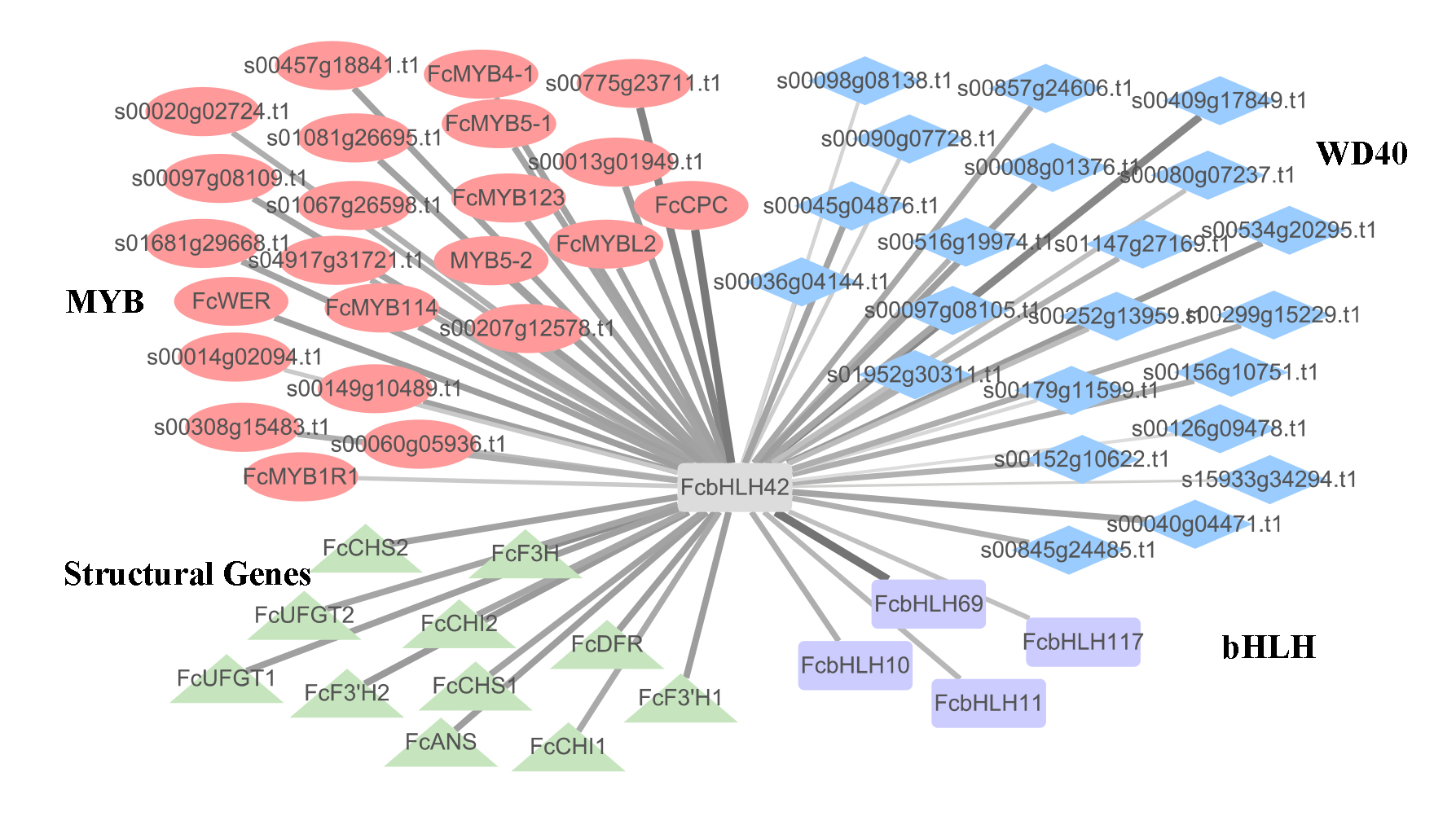

Supplement: Supplementary file 3 [file Image_3.JPEG]

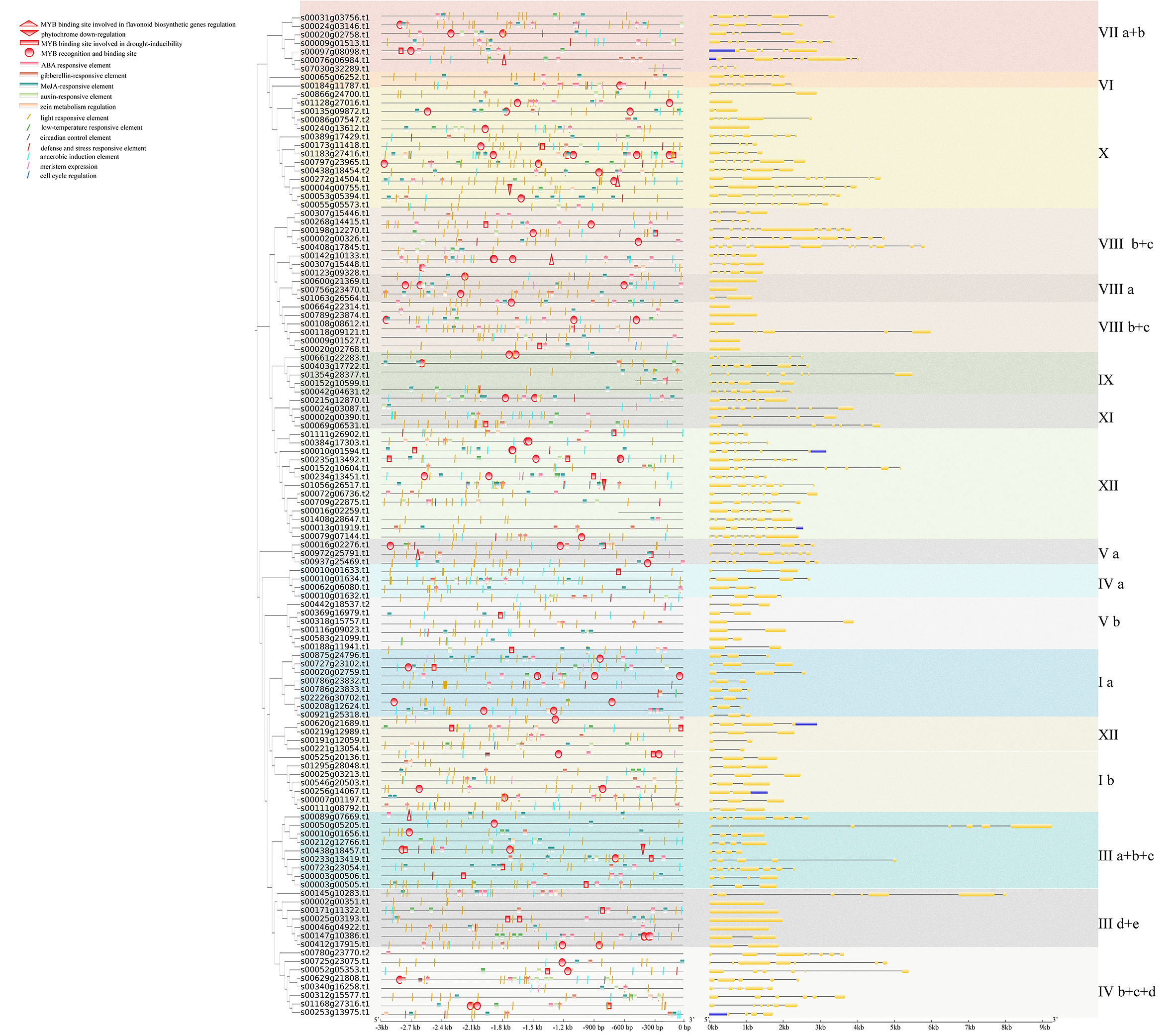

Supplement: Supplementary file 4 [file Image_4.JPEG]
