# Supplementary material for: Genome-Wide Characterization and Analysis of bHLH Transcription Factors Related to Anthocyanin Biosynthesis in Fig (Ficus carica L.)
Source: Front Plant Sci. 2021 Oct 8;12:730692. doi: 10.3389/fpls.2021.730692 (PMC8531510; doi:10.3389/fpls.2021.730692)
Supplement: Supplementary file 5 [file Data_Sheet_1.docx]

Supplementary Material

# Supplementary Figures and Tables

## Supplementary Figures

**
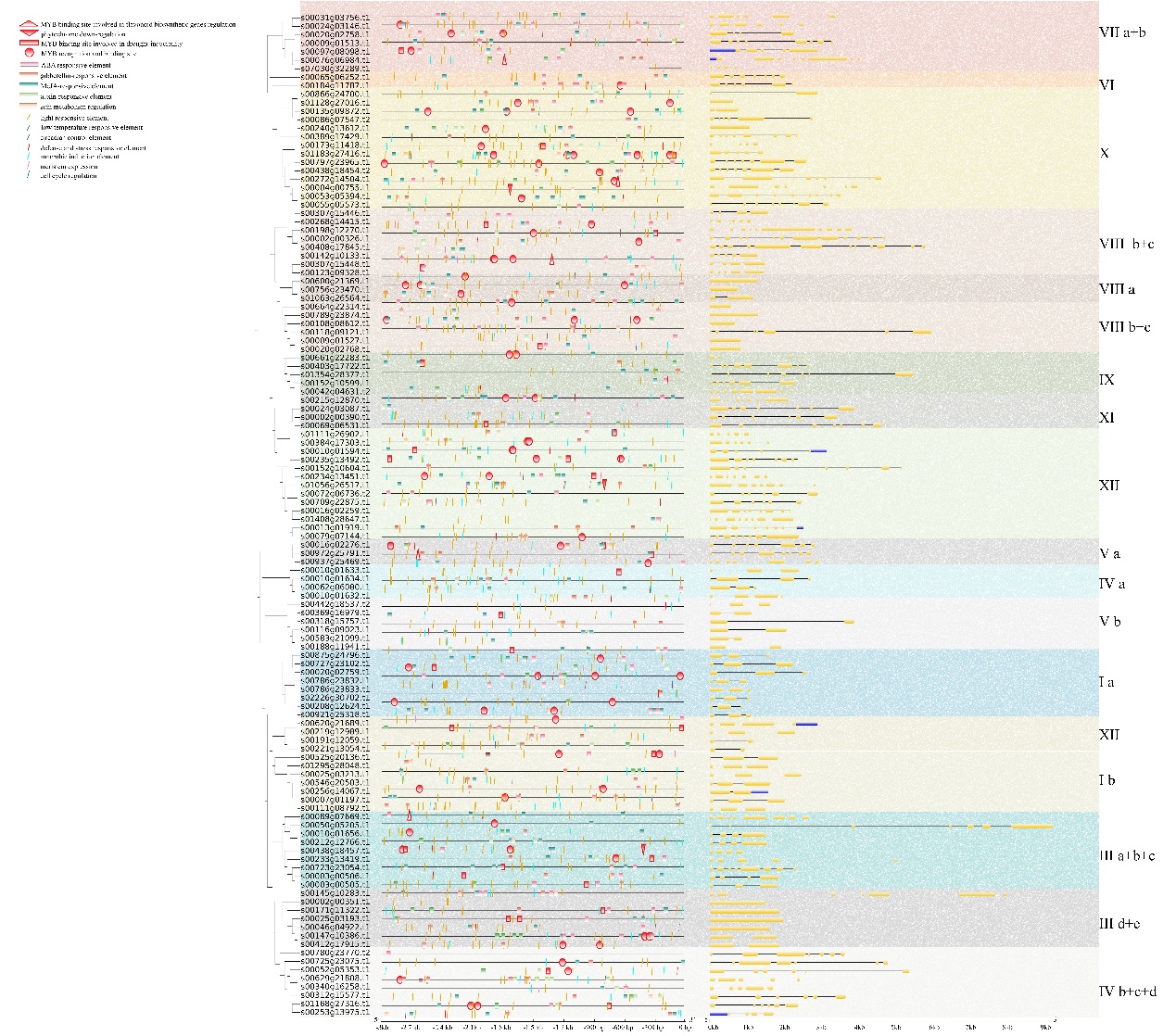
**

**Supplementary Figure 1.** **Gene structure of FcbHLHs and phylogenetic relationship.**The unrooted Neighbor-joining phylogenetic tree was constructed with MEGA6 using the full-length amino acid sequences of 118 FcbHLH proteins. The distribution of predicted cis-elements in the 2 kb upstream promoter regions of FcbHLH genes are depicted. Different cis-elements are shown by marks of different shapes and colors. Exon–intron organization of FctbHLH genes is shown by yellow boxes for exons and black lines of the same length for introns. The upstream and downstream regions of FcbHLH genes are indicated by blue boxes. The size of exons can be estimated by the scale at the bottom.


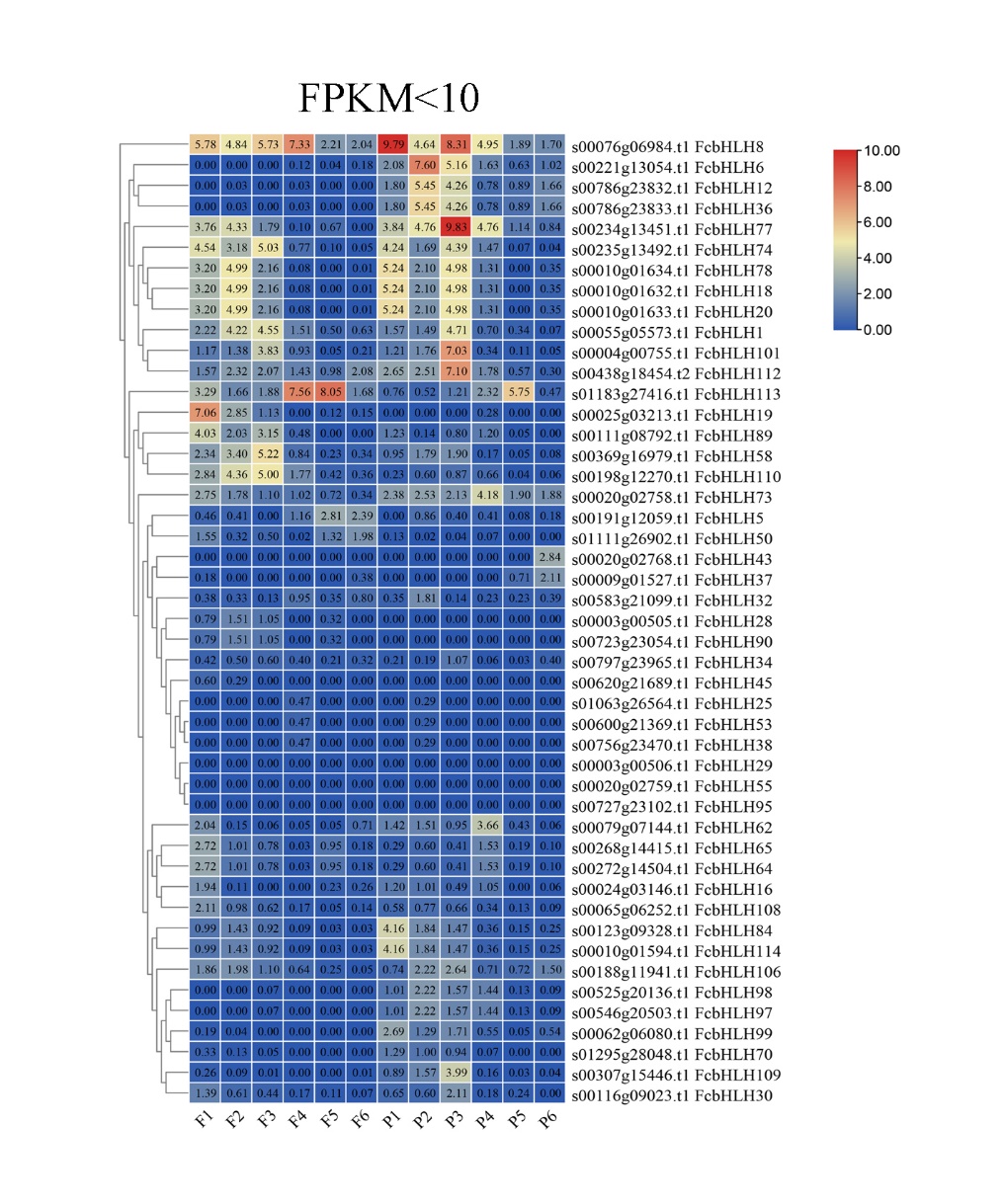


**Supplementary Figure 2.** **Expression profile of FcbHLH genes (FPKM < 10) in the female flower tissue and peel of fig fruit.**The expression of FcbHLH genes is expressed by FPKM value. The hierarchical clustering method and the average linkage method are used to construct the clustering tree. F1-F6 and P1-P6 represent the six stages of Purple Peel female flower tissue and peel respectively during fig fruit development.


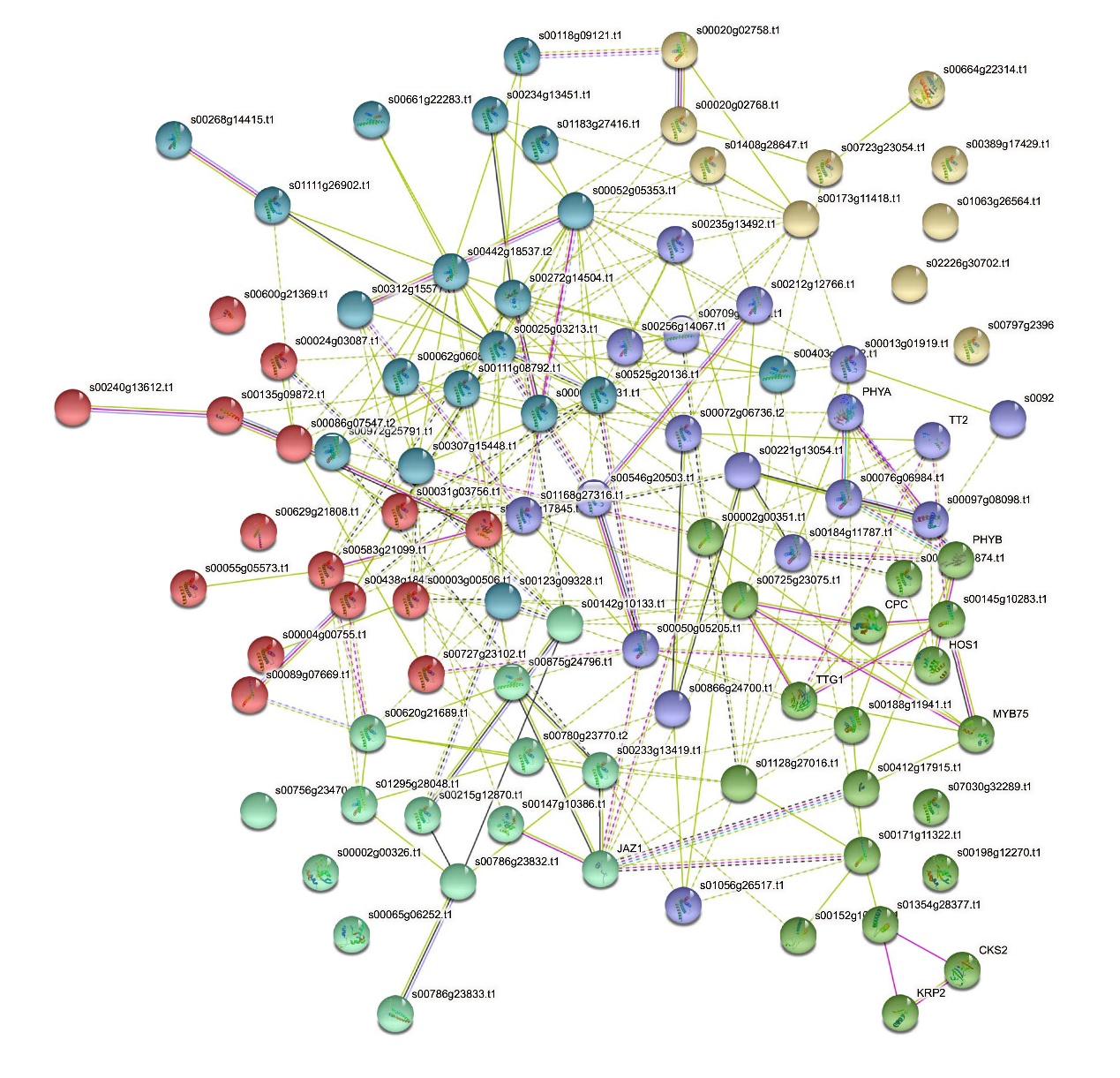


**Supplementary Figure 3.** **The interaction network of FcbHLHs according to the orthologues in Arabidopsis.** The network is predicted by the online software STRING. FcbHLH protein is shown by gene ID. The clusters have been generated using Kmean clustering algorithm from STRING database. Different colors indicate different clusters.


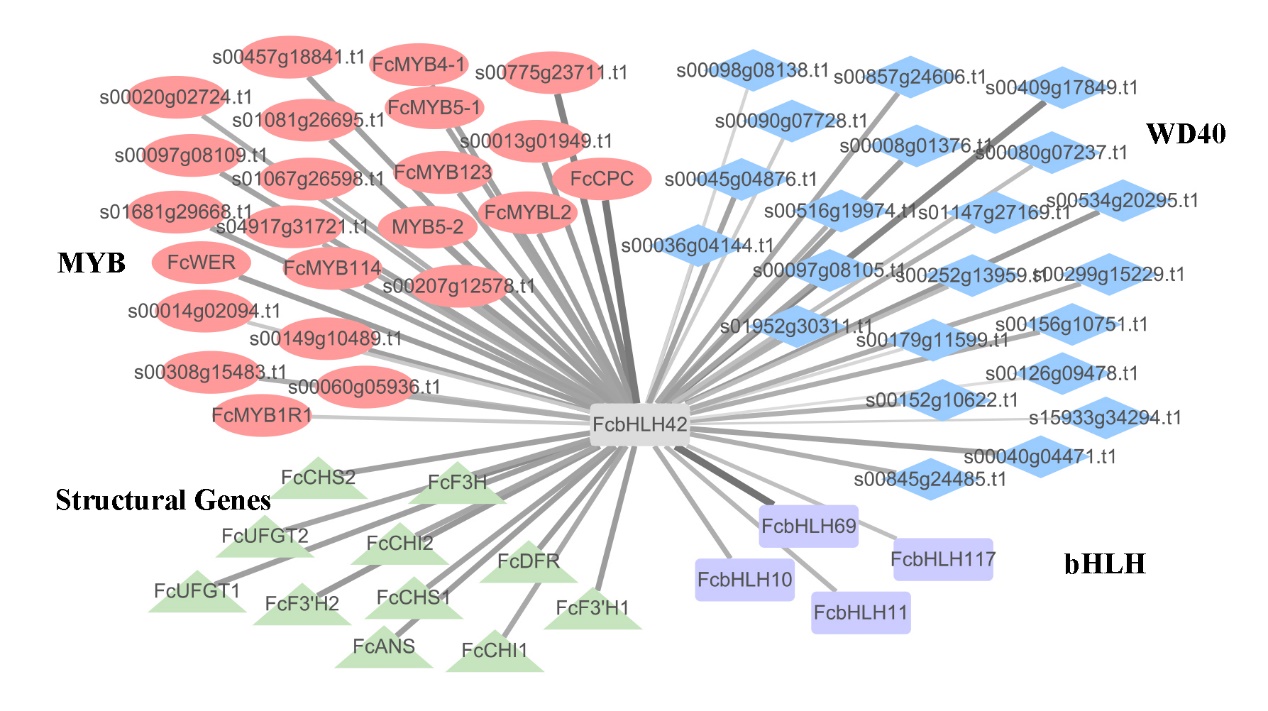


**Supplementary Figure 4. Positive co-expression co-expression network of the FcbHLH42 and anthocyanin-related genes.** The straight line represents the co-expression relationship, and the thicker the line represents the stronger ralationship. The red node of the co-expression ellipse represents the MYB transcription factor. The blue diamond represents the WD40 transcription factor. The green triangle represents the structural gene for anthocyanin synthesis. Correlation coefficient> 0.5 and p <0.001.

## Supplementary Tables

**Table S1.** **The FcbHLH gene family and their protein characteristics**

| Gene | Gene locus ID | Subject_ID | CDS Length (bp) | | Chr | | Protein (aa) | Molecu-lar  weight (Da) | PI | Instability Index | Aliphatic index | | GRAVY |
| --- | --- | --- | --- | --- | --- | --- | --- | --- | --- | --- | --- | --- | --- |
| *FcMYC2* | s00025g03193.t1 | c5167_gl | 2001 | 10 | | 666 | | 72.56 | 5.63 | 47.69 | 64.34 | -0.674 | |
| *FcbHLH1* | s00055g05573.t1 | c42734_gl | 876 | 1 | | 291 | | 32.12 | 6.42 | 58.28 | 60.03 | -0.734 | |
| *FcbHLH2* | s00937g25469.t1 | c44499_g1 | 978 | 5 | | 325 | | 36.09 | 5.69 | 58.29 | 67.45 | -0.806 | |
| *FcbHLH3* | s00002g00351.t1 | c8986_g1 | 1497 | 10 | | 498 | | 55.06 | 5.78 | 46.23 | 80.00 | -0.362 | |
| *FcbHLH4* | s00013g01919.t1 | c5179_g1 | 933 | 6 | | 310 | | 34.90 | 7.30 | 48.92 | 69.16 | -0.859 | |
| *FcbHLH5* | s00191g12059.t1 | c15764_gl | 276 | 5 | | 91 | | 10.25 | 9.09 | 64.03 | 102.86 | -0.488 | |
| *FcbHLH6* | s00221g13054.t1 | c35867_gl | 288 | 11 | | 95 | | 10.82 | 9.09 | 76.72 | 94.42 | -0.633 | |
| *FcbHLH7* | s00002g00390.t1 | c46738_g2 | 894 | 10 | | 297 | | 31.34 | 5.80 | 49.26 | 76.57 | -0.332 | |
| *FcbHLH8* | s00076g06984.t1 | c6130_gl | 2082 | 3 | | 693 | | 73.80 | 5.46 | 48.54 | 59.80 | -0.511 | |
| *FcbHLH9* | s00152g10599.t1 | c40176_g2 | 1134 | 13 | | 377 | | 40.98 | 8.93 | 47.38 | 65.20 | -0.669 | |
| *FcbHLH10* | s00403g17722.t1 | c33434_g2 | 1095 | 2 | | 364 | | 41.32 | 9.22 | 53.00 | 56.76 | -0.934 | |
| *FcbHLH11* | s01354g28377.t1 | c44069_g1 | 915 | 8 | | 304 | | 32.91 | 9.00 | 59.87 | 60.69 | -0.696 | |
| *FcbHLH12* | s00786g23832.t1 | c39257_g1 | 615 | 4 | | 204 | | 22.89 | 5.75 | 62.39 | 82.65 | -0.571 | |
| *FcbHLH13* | s00042g04631.t2 | C41429_gl | 1338 | 3 | | 445 | | 49.93 | 6.77 | 63.93 | 52.63 | -0.937 | |
| *FcbHLH14* | s00046g04922.t1 | c43836_g7 | 1617 | 7 | | 538 | | 59.81 | 5.36 | 40.84 | 83.88 | -0.532 | |
| *FcbHLH15* | s00097g08098.t1 | c43324_g2 | 1356 | FCD_594 | | 451 | | 49.94 | 7.62 | 65.72 | 56.21 | -0.658 | |
| *FcbHLH16* | s00024g03146.t1 | c35383_g2 | 984 | 9 | | 327 | | 35.88 | 7.70 | 58.03 | 57.68 | -0.690 | |
| *FcbHLH17* | s00171g11322.t1 | C43117_gl | 1878 | 9 | | 625 | | 69.02 | 5.84 | 41.51 | 80.94 | -0.473 | |
| *FcbHLH18* | s00010g01632.t1 | c43454_g1 | 1119 | 5 | | 372 | | 41.68 | 6.28 | 54.61 | 75.27 | -0.585 | |
| *FcbHLH19* | s00025g03213.t1 | c68077_gl | 1134 | 10 | | 377 | | 38.87 | 7.04 | 51.29 | 77.64 | -0.451 | |
| *FcbHLH20* | s00010g01633.t1 | c43454_g1 | 981 | 5 | | 326 | | 36.43 | 5.39 | 57.33 | 88.16 | -0.305 | |
| *FcbHLH21* | s00089g07669.t1 | c33397_gl | 1728 | 10 | | 575 | | 64.75 | 5.24 | 46.85 | 72.57 | -0.656 | |
| *FcbHLH22* | s00438g18457.t1 | c33397_gl | 615 | 1 | | 204 | | 22.94 | 6.13 | 46.41 | 86.08 | -0.385 | |
| *FcbHLH23* | s07030g32289.t1 | c41537_g2 | 300 | 10 | | 99 | | 11.53 | 9.18 | 73.11 | 68.99 | -0.888 | |
| *FcbHLH24* | s00009g01513.t1 | c39304_g2 | 1155 | 5 | | 384 | | 41.85 | 4.85 | 67.52 | 78.07 | -0.464 | |
| *FcbHLH25* | s01063g26564.t1 | c35145_g1 | 864 | 1 | | 287 | | 32.48 | 6.50 | 63.90 | 70.31 | -0.570 | |
| *FcbHLH26* | s00184g11787.t1 | c38854_g2 | 924 | 10 | | 307 | | 34.83 | 8.93 | 73.59 | 63.84 | -0.812 | |
| *FcbHLH27* | s00921g25318.t1 | c78231_g1 | 609 | 5 | | 202 | | 23.12 | 7.03 | 54.01 | 89.26 | -0.488 | |
| *FcbHLH28* | s00003g00505.t1 | c2930_g1 | 777 | 3 | | 258 | | 28.84 | 4.59 | 41.62 | 80.16 | -0.507 | |
| *FcbHLH29* | s00003g00506.t1 | c65636_g1 | 855 | 3 | | 284 | | 31.34 | 4.59 | 42.54 | 77.92 | -0.457 | |
| *FcbHLH30* | s00116g09023.t1 | c73055_gl | 1071 | 11 | | 356 | | 39.84 | 6.30 | 63.50 | 77.33 | -0.699 | |
| *FcbHLH31* | s00072g06736.t2 | c33809_gl | 846 | 6 | | 281 | | 30.25 | 5.73 | 60.33 | 66.05 | -0.634 | |
| *FcbHLH32* | s00583g21099.t1 | c49645_g1 | 759 | 7 | | 252 | | 27.33 | 7.67 | 53.99 | 74.64 | -0.495 | |
| *FcbHLH33* | s00050g05205.t1 | c33397_gl | 1797 | 5 | | 598 | | 64.58 | 5.71 | 61.08 | 76.99 | -0.364 | |
| *FcbHLH34* | s00797g23965.t1 | c28748_g1 | 1449 | 2 | | 482 | | 52.64 | 6.02 | 62.01 | 53.88 | -0.682 | |
| *FcbHLH35* | s00233g13419.t1 | c36074_gl | 753 | 5 | | 250 | | 28.45 | 5.64 | 62.39 | 86.64 | -0.508 | |
| *FcbHLH36* | s00786g23833.t1 | c39257_g1 | 792 | 4 | | 263 | | 29.14 | 8.58 | 58.00 | 80.80 | -0.595 | |
| *FcbHLH37* | s00009g01527.t1 | c74440_g1 | 846 | 5 | | 281 | | 30.96 | 9.54 | 56.17 | 69.82 | -0.415 | |
| *FcbHLH38* | s00756g23470.t1 | c35145_g1 | 759 | 1 | | 252 | | 28.42 | 8.59 | 57.31 | 70.87 | -0.518 | |
| *FcbHLH39* | s00725g23075.t1 | c43678_g1 | 1650 | 9 | | 549 | | 61.47 | 5.47 | 43.12 | 72.84 | -0.603 | |
| *FcbHLH40* | s00118g09121.t1 | C47035_gl | 1161 | 5 | | 386 | | 42.71 | 9.42 | 58.35 | 83.42 | -0.277 | |
| *FcbHLH41* | s00780g23770.t2 | c45894_g1 | 1698 | 9 | | 565 | | 63.68 | 6.59 | 64.36 | 85.42 | -0.390 | |
| *FcbHLH42* | s00145g10283.t1 | c46354_gl | 2151 | 10 | | 716 | | 78.91 | 5.08 | 63.77 | 72.84 | -0.550 | |
| *FcbHLH43* | s00020g02768.t1 | c55722_g1 | 843 | 7 | | 280 | | 30.68 | 8.40 | 53.93 | 72.50 | -0.375 | |
| *FcbHLH44* | s00629g21808.t1 | c26867_g1 | 942 | 5 | | 313 | | 34.66 | 5.92 | 59.09 | 50.80 | -1.005 | |
| *FcbHLH45* | s00620g21689.t1 | c83412_g1 | 1479 | 7 | | 492 | | 55.21 | 5.99 | 56.29 | 77.70 | -0.589 | |
| *FcbHLH46* | s00972g25791.t1 | c39032_g3 | 1122 | 6 | | 373 | | 41.03 | 7.49 | 56.19 | 66.94 | -0.764 | |
| *FcbHLH47* | s00052g05353.t1 | c29441_gl | 966 | 6 | | 321 | | 35.93 | 6.41 | 44.66 | 81.43 | -0.517 | |
| *FcbHLH48* | s00152g10604.t1 | c35607_gl | 1134 | 13 | | 377 | | 41.47 | 5.65 | 56.45 | 69.97 | -0.667 | |
| *FcbHLH49* | s01408g28647.t1 | c43347_g2 | 1353 | 8 | | 450 | | 48.58 | 9.06 | 50.50 | 56.40 | -0.763 | |
| *FcbHLH50* | s01111g26902.t1 | c39854_g1 | 504 | 6 | | 167 | | 18.87 | 9.10 | 53.05 | 65.33 | -0.726 | |
| *FcbHLH51* | s00442g18537.t2 | c21697_gl | 693 | 4 | | 230 | | 25.70 | 6.55 | 41.66 | 89.87 | -0.445 | |
| *FcbHLH52* | s00412g17915.t1 | c32587_gl | 1485 | 12 | | 494 | | 55.18 | 6.29 | 38.58 | 79.03 | -0.421 | |
| *FcbHLH53* | s00600g21369.t1 | c35145_g1 | 1290 | 5 | | 429 | | 46.75 | 8.76 | 68.66 | 74.66 | -0.318 | |
| *FcbHLH54* | s00307g15448.t1 | c13701_gl | 1047 | 10 | | 348 | | 37.78 | 6.05 | 53.63 | 70.14 | -0.563 | |
| *FcbHLH55* | s00020g02759.t1 | c27653_g2 | 822 | 7 | | 273 | | 30.69 | 7.73 | 51.60 | 86.56 | -0.421 | |
| *FcbHLH56* | s00408g17845.t1 | c46710_gl | 2679 | 3 | | 892 | | 97.20 | 6.61 | 41.98 | 82.94 | -0.279 | |
| *FcbHLH57* | s00389g17429.t1 | c41678_gl | 852 | 4 | | 283 | | 32.08 | 7.76 | 64.47 | 78.20 | -0.586 | |
| *FcbHLH58* | s00369g16979.t1 | c39506_gl | 768 | 5 | | 255 | | 28.73 | 7.71 | 47.08 | 85.69 | -0.431 | |
| *FcbHLH59* | s00340g16258.t1 | c45096_gl | 990 | 10 | | 329 | | 36.55 | 6.47 | 71.22 | 59.85 | -0.988 | |
| *FcbHLH60* | s00709g22875.t1 | c37030_g2 | 945 | 11 | | 314 | | 34.34 | 7.07 | 53.44 | 60.92 | -0.818 | |
| *FcbHLH61* | s00318g15757.t1 | cl2197_gl | 777 | 2 | | 258 | | 27.63 | 9.16 | 45.23 | 85.19 | -0.198 | |
| *FcbHLH62* | s00079g07144.t1 | C72970_gl | 1650 | 10 | | 549 | | 59.77 | 6.83 | 48.72 | 63.32 | -0.715 | |
| *FcbHLH63* | s01056g26517.t1 | c44198_g1 | 1416 | 10 | | 471 | | 50.96 | 5.92 | 55.06 | 67.35 | -0.474 | |
| *FcbHLH64* | s00272g14504.t1 | c43008_32 | 1671 | 11 | | 556 | | 60.52 | 6.34 | 46.78 | 63.02 | -0.701 | |
| *FcbHLH65* | s00268g14415.t1 | c43008_g2 | 420 | 11 | | 139 | | 15.48 | 9.16 | 45.16 | 66.62 | -0.647 | |
| *FcbHLH66* | s00024g03087.t1 | c4782_g2 | 1449 | 11 | | 482 | | 48.43 | 6.20 | 55.08 | 55.58 | -0.612 | |
| *FcbHLH67* | s00256g14067.t1 | c31437_gl | 1041 | 5 | | 346 | | 39.05 | 6.18 | 72.04 | 70.78 | -0.661 | |
| *FcbHLH68* | s00053g05394.t1 | c39396_gl | 1155 | 9 | | 384 | | 41.86 | 6.97 | 61.74 | 57.94 | -0.694 | |
| *FcbHLH69* | s00240g13612.t1 | c40876_g3 | 1092 | 7 | | 363 | | 40.06 | 4.80 | 50.21 | 66.72 | -0.686 | |
| *FcbHLH70* | s01295g28048.t1 | c50669_g1 | 1188 | 9 | | 395 | | 44.44 | 6.65 | 64.20 | 78.51 | -0.674 | |
| *FcbHLH71* | s00219g12989.t1 | c46354_gl | 1038 | 2 | | 345 | | 38.55 | 6.27 | 46.42 | 79.42 | -0.564 | |
| *FcbHLH72* | s00031g03756.t1 | c45594_gl | 1338 | 6 | | 445 | | 48.63 | 8.42 | 65.68 | 55.73 | -0.700 | |
| *FcbHLH73* | s00020g02758.t1 | c41379_g1 | 957 | 7 | | 318 | | 34.46 | 5.40 | 60.56 | 62.61 | -0.520 | |
| *FcbHLH74* | s00235g13492.t1 | c46293_g2 | 1233 | 7 | | 410 | | 44.16 | 5.50 | 51.40 | 65.85 | -0.594 | |
| *FcbHLH75* | s00384g17303.t1 | c47311_gl | 714 | 5 | | 237 | | 27.13 | 6.56 | 53.11 | 70.76 | -0.868 | |
| *FcbHLH76* | s00016g02259.t1 | c45350_g1 | 1278 | 11 | | 425 | | 45.63 | 5.73 | 53.59 | 67.74 | -0.618 | |
| *FcbHLH77* | s00234g13451.t1 | c38658_gl | 732 | 11 | | 243 | | 27.41 | 6.61 | 50.99 | 69.84 | -0.642 | |
| *FcbHLH78* | s00010g01634.t1 | c43454_g1 | 1098 | 5 | | 365 | | 40.73 | 7.03 | 55.21 | 72.90 | -0.508 | |
| *FcbHLH79* | s00007g01197.t1 | c31437_g1 | 966 | 5 | | 321 | | 36.35 | 6.32 | 54.86 | 84.61 | -0.365 | |
| *FcbHLH80* | s00212g12766.t1 | c25231_gl | 1119 | 7 | | 372 | | 42.05 | 5.05 | 62.79 | 76.29 | -0.519 | |
| *FcbHLH81* | s00661g22283.t1 | c42681_g1 | 312 | 13 | | 103 | | 12.10 | 8.42 | 49.99 | 73.79 | -0.937 | |
| *FcbHLH82* | s00215g12870.t1 | c13701_gl | 1278 | 9 | | 425 | | 46.00 | 4.70 | 49.59 | 71.15 | -0.507 | |
| *FcbHLH83* | s00142g10133.t1 | c13701_gl | 858 | 5 | | 285 | | 31.70 | 5.70 | 59.79 | 59.23 | -0.720 | |
| *FcbHLH84* | s00123g09328.t1 | c4782_g2 | 1014 | 7 | | 337 | | 37.38 | 4.80 | 49.72 | 66.82 | -0.654 | |
| *FcbHLH85* | s00208g12624.t1 | c39193_g2 | 501 | 1 | | 166 | | 18.46 | 5.60 | 24.60 | 90.96 | -0.299 | |
| *FcbHLH86* | s00147g10386.t1 | C32587_gl | 1266 | 12 | | 421 | | 46.93 | 6.22 | 41.05 | 83.49 | -0.235 | |
| *FcbHLH87* | s00789g23874.t1 | c27878_g2 | 1305 | 1 | | 434 | | 47.03 | 6.08 | 54.02 | 69.70 | -0.538 | |
| *FcbHLH88* | s00135g09872.t1 | C41048_g2 | 738 | 1 | | 245 | | 27.20 | 5.93 | 58.27 | 58.94 | -0.769 | |
| *FcbHLH89* | s00111g08792.t1 | C41970_gl | 1074 | 5 | | 357 | | 39.69 | 5.29 | 57.62 | 69.66 | -0.536 | |
| *FcbHLH90* | s00723g23054.t1 | c2930_g1 | 1398 | 11 | | 465 | | 52.12 | 7.47 | 57.91 | 78.22 | -0.505 | |
| *FcbHLH91* | s00108g08612.t1 | c27878_2 | 687 | 5 | | 228 | | 25.83 | 6.18 | 63.59 | 71.10 | -0.682 | |
| *FcbHLH92* | s00875g24796.t1 | c25473_g1 | 711 | 8 | | 236 | | 27.11 | 9.07 | 52.62 | 78.09 | -0.592 | |
| *FcbHLH93* | s00010g01656.t1 | c25231_g1 | 1065 | 5 | | 354 | | 39.75 | 4.84 | 59.74 | 75.20 | -0.515 | |
| *FcbHLH94* | s00086g07547.t2 | c408762_gl | 1077 | 10 | | 358 | | 39.89 | 4.83 | 42.79 | 85.22 | -0.442 | |
| *FcbHLH95* | s00727g23102.t1 | c27653_g2 | 1104 | 10 | | 367 | | 39.73 | 5.81 | 51.18 | 63.87 | -0.620 | |
| *FcbHLH96* | s00069g06531.t1 | c37082_gl | 978 | 3 | | 325 | | 34.37 | 5.92 | 58.57 | 73.88 | -0.347 | |
| *FcbHLH97* | s00546g20503.t1 | c40191_gl | 1263 | 8 | | 420 | | 48.23 | 5.53 | 70.23 | 71.26 | -0.738 | |
| *FcbHLH98* | s00525g20136.t1 | c40191_gl | 1128 | 4 | | 375 | | 40.65 | 5.45 | 53.77 | 82.69 | -0.357 | |
| *FcbHLH99* | s00062g06080.t1 | c10087_gl | 1002 | 10 | | 333 | | 37.26 | 8.51 | 41.29 | 87.81 | -0.370 | |
| *FcbHLH100* | s02226g30702.t1 | c45644_g1 | 561 | 6 | | 186 | | 21.10 | 7.80 | 49.75 | 93.23 | -0.355 | |
| *FcbHLH101* | s00004g00755.t1 | c40531_g1 | 1431 | 7 | | 476 | | 51.86 | 8.08 | 51.31 | 61.03 | -0.605 | |
| *FcbHLH102* | s00016g02276.t1 | c24772_g1 | 990 | 11 | | 329 | | 36.31 | 5.80 | 51.93 | 60.49 | -1.014 | |
| *FcbHLH103* | s00002g00326.t1 | c580_g1 | 2232 | 10 | | 743 | | 82.61 | 5.85 | 46.21 | 80.38 | -0.396 | |
| *FcbHLH104* | s00312g15577.t1 | c46352_gl | 1017 | 8 | | 338 | | 38.07 | 5.11 | 49.65 | 67.34 | -0.643 | |
| *FcbHLH105* | s01168g27316.t1 | c43844_g1 | 855 | 5 | | 284 | | 32.09 | 5.74 | 58.41 | 71.76 | -0.700 | |
| *FcbHLH106* | s00188g11941.t1 | c39646_gl | 744 | FCD_498 | | 247 | | 27.69 | 8.53 | 52.28 | 88.83 | -0.533 | |
| *FcbHLH107* | s00173g11418.t1 | C42522_g3 | 306 | 1 | | 101 | | 11.46 | 9.15 | 34.27 | 90.79 | -0.014 | |
| *FcbHLH108* | s00065g06252.t1 | c39726_gl | 1032 | 10 | | 343 | | 38.69 | 5.15 | 57.01 | 75.13 | -0.527 | |
| *FcbHLH109* | s00307g15446.t1 | c31149_gl | 924 | 10 | | 307 | | 33.11 | 4.94 | 48.01 | 65.83 | -0.649 | |
| *FcbHLH110* | s00198g12270.t1 | C42599_gl | 2418 | 12 | | 805 | | 88.41 | 5.09 | 45.38 | 78.61 | -0.381 | |
| *FcbHLH111* | s00866g24700.t1 | c40571_g1 | 633 | 8 | | 210 | | 23.49 | 11.53 | 46.12 | 82.33 | -0.562 | |
| *FcbHLH112* | s00438g18454.t2 | c43099_gl | 1512 | 1 | | 503 | | 54.82 | 6.18 | 54.69 | 61.23 | -0.594 | |
| *FcbHLH113* | s01183g27416.t1 | c40266_g1 | 762 | 10 | | 253 | | 27.35 | 7.51 | 51.22 | 63.64 | -0.484 | |
| *FcbHLH114* | s00010g01594.t1 | c4898_g1 | 558 | 5 | | 185 | | 21.13 | 7.76 | 43.07 | 67.51 | -0.906 | |
| *FcbHLH115* | s00253g13975.t1 | c40580_gl | 579 | 2 | | 192 | | 20.99 | 9.52 | 53.01 | 76.30 | -0.723 | |
| *FcbHLH116* | s00664g22314.t1 | c27878_g2 | 567 | 12 | | 188 | | 21.40 | 7.26 | 67.34 | 60.11 | -0.815 | |
| *FcbHLH117* | s01128g27016.t1 | c78643_g1 | 633 | 3 | | 210 | | 23.32 | 11.35 | 64.33 | 80.33 | -0.579 | |

**Table S2. Sequences of 10 predicted motifs of FcbHLH proteins**

| Motif | Best possible match | Mumbers of *FcbHLH* protein conserved motifs |
| --- | --- | --- |
| 1 | K[TM]D[KT]AS[MI]L[DG][ED]A[IV]XY[VI]K[FE]LQRQ | 40,80,91,66,37,73,24,43,15,64,78,72,8,53,99,101,34,23,112,116,87,29,68,1,96,18,7,76,49,35,21,62,114,42,30,26,119,107,74,4,3,84,54,81,17,63,46,33,MYC2,90,109,20,28,2,60,31,50,39,45,102,13,9,32,83,86,52,38,58,57,93,89,80,14,48,106,71,61,108,25,77,75,79,65,51,98,70,67,95,55,104,88,97,19,115,22,10,44,11,69,59,110,105,47,6,5,41,36,92,27,111,103,56,85,117,12,100,94 |
| 2 | XSHSXAERRRRE[KR][IL][NS][ED]R[LFM]KAL [QR]SLVP[NG]XX | 114,66,49,62,60,31,63,96,4,74,48,76,50,75,8,7,77,84,82,43,40,37,17,3,23,9,42,MYC2,83,21,86,14,52,13,81,15,26,54,87,116,102,24,97,30,35,16,33,63,81,72,61,46,98,29,28,93,80,11,19,99,67,78,58,41,18,2,89,22,10,51,70,20,106,90,79,25,45,55,38,73,34,108,113,71,115,112,104,101,68,53,95,105,103,1,69,27,56,59,92,36,44,110,107,12,88,57,5,94,100,64,47,6 |
| 3 | [KP]S[DV][YP][IC][HR]VRA[RK]RGQAT | 62,49,60,31,77,4,63,74,76,75,48,50,81,65,9,7,96,13,11,82,66,10 |
| 4 | L[PA][DE][IV][ED]V[KRT][IV][VI][GDE]T[DEH][AV][LM][IL][RK][IV]Q[CS][PE][RK] | MYC2,86,70,52,18,78,99,20,19,67,89,97,14,71,45,79,17,55,98,39,3,42,93 |
| 5 | [PQ]GLLLK[LI][MI]XAL[EQ]XL[GH]L[DE][VI][LV]HA[SN][IV][ST]T[FV][NG][GD]R | 33,93,98,97,90,80,21,58,14,19,MYC2,67,70,86,52,18,79,61,51,102,100,99,30,35,55,95,89,106,86,46,78,27,42,20,2,39,22,12,17 |
| 6 | C[DE][RS][AV][FK][EFL]A[RHQ][MS][HA]GIQT[LVI]VC[IV]P[MT][LP][ND]GV[VL]ELG[ST][TS]D[LS][IV]TE[DS][WL][SG]L[VL]Q | 52,86,14,MYC2,17,39,42,3 |
| 7 | [AP][KM]QDL[RQ]S[KR]GLCL[VM]P[IV]S[CL][TA]SA[VI] | 112,68,34,64,66,101,57,113,7,96,82,108 |
| 8 | VEFLSMKL[AE][ATS][VA]N[PS]R[ML] | 49,63,76,31,74,62,48,4,50,60,65,77,75 |
| 9 | [MIL][AG][AQ]M[KR]EM[IM][YF][RGK][IA]A[AV][MF][QR]P[VI][DHN][IL][DG][PL][EA][STI][ITV][KEPR][KPR]P[KR]R[RK]NV[RK]IS[DKT] | 37,40,91,43,87 |
| 10 | [DE][DI][LV]TD[IT][ED]WF[YF][LT][MV]S[VM]T[RF][ST]F[PS]A[GE]SG[AL]PG[KR][AS]YSSGA[HY]VW[LV][TS]G[AN] | 86,52,14,42,17,MYC2 |

**Table S3. The gene tandem replication on *Ficus carica* L. chromosome**

| **Gene ID** | | **Gene name** | | **Chr.** |
| --- | --- | --- | --- | --- |
| **gene1** | **gene2** | **gene1** | **gene2** |  |
| s00756g23470.t1 | s00002g00351.t1 | *FcbHLH38* | *FcbHLH3* | **1** |
| s00438g18454.t2 | s00438g18457.t1 | *FcbHLH112* | *FcbHLH22* | **1** |
| s00003g00505.t1 | s00003g00506.t1 | *FcbHLH28* | *FcbHLH29* | **3** |
| s00786g23832.t1 | s00786g23833.t1 | *FcbHLH12* | *FcbHLH36* | **4** |
| s01168g27316.t1 | s00525g20136.t1 | *FcbHLH105* | *FcbHLH98* | **5** |
| s00010g01632.t1 | s00010g01634.t1 | *FcbHLH18* | *FcbHLH78* | **5** |
| s00010g01633.t1 | s00010g01634.t1 | *FcbHLH20* | *FcbHLH78* | **5** |
| s00009g01513.t1 | s00009g01527.t1 | *FcbHLH24* | *FcbHLH37* | **5** |
| s00111g08792.t1 | s00256g14067.t1 | *FcbHLH89* | *FcbHLH67* | **5** |
| s00065g06252.t1 | s01354g28377.t1 | *FcbHLH108* | *FcbHLH11* | **10** |
| s00025g03193.t1 | s00184g11787.t1 | *FcMYC2* | *FcbHLH26* | **10** |
| s00307g15446.t1 | s00307g15448.t1 | *FcbHLH109* | *FcbHLH54* | **10** |
| s00016g02259.t1 | s00709g22875.t1 | *FcbHLH76* | *FcbHLH60* | **11** |
| s00937g25469.t1 | s00972g25791.t1 | *FcbHLH2* | *FcbHLH46* | **13** |
| s00152g10599.t1 | s00152g10604.t1 | *FcbHLH9* | *FcbHLH48* | **13** |

**Table S4. Synteny blocks of bHLH genes within *Ficus carica* L. genomes**

| **gene in the synteny region** | | **gene name** | |
| --- | --- | --- | --- |
| **gene1** | **gene2** | **gene1** | **gene2** |
| FCD_00012578 | FCD_00019987 | *FcbHLH85* | *FcbHLH27* |
| FCD_00005674 | FCD_00017083 | *FcbHLH1* | *FcbHLH68* |
| FCD_00007228 | FCD_00023157 | *FcbHLH61* | *FcbHLH58* |
| FCD_00006043 | FCD_00009057 | *FcbHLH93* | *FcbHLH80* |
| FCD_00006322 | FCD_00003893 | *FcbHLH24* | *FcbHLH73* |
| FCD_00006339 | FCD_00003878 | *FcbHLH37* | *FcbHLH43* |
| FCD_00024689 | FCD_00018799 | *FcbHLH50* | *FcbHLH65* |
| FCD_00009145 | FCD_00022600 | *FcbHLH69* | *FcbHLH94* |
| FCD_00004019 | FCD_00009482 | *FcbHLH84* | *FcbHLH109* |
| FCD_00003890 | FCD_00014303 | *FcbHLH55* | *FcbHLH55* |
| FCD_00021979 | FCD_00004696 | *FcbHLH14* | *FcbHLH52* |

**Table S5. Ka/Ks analysis for the FcbHLH duplicated genes**

| Gene name | Gene name | Ka | Ks | Ka/Ks |
| --- | --- | --- | --- | --- |
| *FcbHLH73* | *FcbHLH24* | 0.5686 | 1.6345 | 0.3479 |
| *FcbHLH43* | *FcbHLH37* | 0.4654 | 1.4651 | 0.3176 |
| *FcbHLH26* | *FcbHLH37* | 0.9186 | 6.0562 | 0.1517 |
| *FcbHLH93* | *FcbHLH80* | 0.3625 | 2.8230 | 0.1284 |
| *FcbHLH102* | *FcbHLH46* | 0.4255 | 2.6585 | 0.1601 |
| *FcbHLH55* | *FcbHLH95* | 0.8151 | 2.8013 | 0.2910 |
| *FcbHLH50* | *FcbHLH13* | 1.1335 | 3.9874 | 0.2843 |
| *FcbHLH52* | *FcbHLH45* | 0.2293 | 1.9395 | 0.1182 |
| *FcbHLH59* | *FcbHLH86* | 0.6506 | 2.5546 | 0.2547 |
| *FcbHLH65* | *FcbHLH66* | 0.6088 | 2.1593 | 0.2819 |
| *FcbHLH85* | *FcbHLH73* | 0.9065 | 3.2934 | 0.2753 |
| *FcbHLH102* | *FcbHLH90* | 0.1440 | 1.8598 | 0.0774 |
| *FcbHLH97* | *FcbHLH84* | 0.3199 | 1.6781 | 0.1906 |
| *FcbHLH81* | *FcbHLH105* | 0.2457 | 1.5244 | 0.1612 |
| *FcbHLH75* | *FcbHLH113* | 0.3945 | 1.4007 | 0.2817 |
| *FcbHLH7* | *FcbHLH66* | 0.7164 | 3.3826 | 0.2118 |

**Table S6. The synteny regions of bHLH genes in diverse species**

|  | Gene in the synteny region | | Gene in the synteny region | | Gene in the synteny region | | Gene in the synteny region | |
| --- | --- | --- | --- | --- | --- | --- | --- | --- |
| No. | *Ficus carica* | *Ficus hispida* | *Ficus carica* | *Ficus microcarpa* | *Ficus carica* | Arabidopsis thaliana | *Ficus carica* | *Vitis vinifera* |
| 1 | s00079g07144.t1 | Fh.10G0007690 | s00079g07144.t1 | Fm.10G0008200 | s00079g07144.t1 | NM_111613.5 | s00184g11787.t1 | VIT_207s0005g05100.3 |
| 2 | s00184g11787.t1 | Fh.10G0006120 | s00184g11787.t1 | Fm.10G0007120 | s00079g07144.t1 | NM_124232.4 | s00002g00326.t1 | VIT_207s0005g03070.1 |
| 3 | s00002g00326.t1 | Fh.10G0004820 | s00002g00326.t1 | Fm.10G0005260 | s00184g11787.t1 | NM_001335663.1 | s00002g00351.t1 | VIT_207s0141g01060.1 |
| 4 | s00002g00351.t1 | Fh.10G0004600 | s00002g00351.t1 | Fm.10G0004970 | s07030g32289.t1 | NM_001341933.1 | s00002g00390.t1 | VIT_214s0030g02230.2 |
| 5 | s00002g00390.t1 | Fh.10G0004280 | s00002g00390.t1 | Fm.10G0004590 | s00002g00326.t1 | NM_001036377.1 | s00069g06531.t1 | VIT_205s0020g04620.1 |
| 6 | s00069g06531.t1 | Fh.03G0018660 | s00069g06531.t1 | Fm.10G0004610 | s00002g00390.t1 | NM_001331380.1 | s00069g06531.t1 | VIT_207s0141g00220.1 |
| 7 | s00145g10283.t1 | Fh.10G0003390 | s00145g10283.t1 | Fm.10G0003820 | s00438g18454.t2 | NM_104847.4 | s00145g10283.t1 | VIT_207s0104g00090.1 |
| 8 | s00065g06252.t1 | Fh.10G0003240 | s00065g06252.t1 | Fm.10G0003390 | s00438g18454.t2 | NM_001340535.1 | s00065g06252.t1 | VIT_207s0104g00250.1 |
| 9 | s00723g23054.t1 | Fh.11G0003670 | s00003g00505.t1 | Fm.03G0018060 | s00438g18454.t2 | NM_118254.4 | s00723g23054.t1 | VIT_201s0127g00650.1 |
| 10 | s00438g18454.t2 | Fh.14G0005110 | s00723g23054.t1 | Fm.11G0003460 | s00438g18457.t1 | NM_118253.1 | s00438g18454.t2 | VIT_219s0014g04670.2 |
| 11 | s00438g18457.t1 | Fh.01G0011120 | s00438g18454.t2 | Fm.01G0012130 | s00020g02768.t1 | NM_126106.3 | s00438g18454.t2 | VIT_200s0274g00070.1 |
| 12 | s00235g13492.t1 | Fh.07G0006800 | s00438g18457.t1 | Fm.01G0012150 | s00020g02758.t1 | NM_126111.4 | s00438g18457.t1 | VIT_200s0274g00045.1 |
| 13 | s00020g02768.t1 | Fh.07G0002720 | s00020g02768.t1 | Fm.03G0032510 | s00123g09328.t1 | NM_102539.3 | s00235g13492.t1 | VIT_218s0001g08600.1 |
| 14 | s00020g02768.t1 | Fh.05G0007410 | s00235g13492.t1 | Fm.03G0033980 | s00123g09328.t1 | NM_148080.2 | s00020g02768.t1 | VIT_218s0001g10400.1 |
| 15 | s00020g02759.t1 | Fh.10G0013240 | s00020g02759.t1 | Fm.03G0032610 | s00525g20136.t1 | NM_124700.3 | s00020g02768.t1 | VIT_207s0031g00550.1 |
| 16 | s00020g02759.t1 | Fh.07G0002630 | s00020g02768.t1 | Fm.05G0008490 | s00786g23832.t1 | NM_001341729.1 | s00020g02759.t1 | VIT_218s0001g10300.1 |
| 17 | s00020g02758.t1 | Fh.07G0002620 | s00020g02758.t1 | Fm.03G0032620 | s00786g23832.t1 | NM_001344958.1 | s00020g02759.t1 | VIT_203s0063g00170.1 |
| 18 | s00020g02758.t1 | Fh.05G0007280 | s00020g02759.t1 | Fm.10G0014450 | s00198g12270.t1 | NM_179586.3 | s00020g02758.t1 | VIT_218s0001g10270.1 |
| 19 | s00123g09328.t1 | Fh.10G0011830 | s00123g09328.t1 | Fm.03G0033430 | s00789g23874.t1 | NM_113028.3 | s00020g02758.t1 | VIT_207s0031g00450.1 |
| 20 | s00123g09328.t1 | Fh.07G0001820 | s00020g02758.t1 | Fm.05G0008650 | s00010g01656.t1 | NM_125962.4 | s00123g09328.t1 | VIT_218s0001g09210.1 |
| 21 | s00525g20136.t1 | Fh.04G0016730 | s00123g09328.t1 | Fm.10G0012910 | s00010g01634.t1 | NM_001335820.1 | s00123g09328.t1 | VIT_203s0091g00730.1 |
| 22 | s00412g17915.t1 | Fh.12G0010890 | s00786g23832.t1 | Fm.04G0012420 | s00010g01634.t1 | NM_119946.4 | s00525g20136.t1 | VIT_216s0050g02500.1 |
| 23 | s00412g17915.t1 | Fh.09G0007950 | s00525g20136.t1 | Fm.04G0015080 | s00009g01513.t1 | NM_119857.3 | s00786g23832.t1 | VIT_216s0022g02240.1 |
| 24 | s00198g12270.t1 | Fh.12G0011190 | s00412g17915.t1 | Fm.09G0009180 | s00009g01513.t1 | NM_126111.4 | s00412g17915.t1 | VIT_215s0048g02820.1 |
| 25 | s00789g23874.t1 | Fh.01G0015640 | s00412g17915.t1 | Fm.12G0011270 | s00009g01527.t1 | NM_114893.3 | s00412g17915.t1 | VIT_202s0025g02610.1 |
| 26 | s00055g05573.t1 | Fh.01G0014850 | s00198g12270.t1 | Fm.12G0011560 | s00009g01527.t1 | NM_126106.3 | s00198g12270.t1 | VIT_202s0025g02310.1 |
| 27 | s00010g01656.t1 | Fh.07G0012950 | s00055g05573.t1 | Fm.01G0016500 | s00089g07669.t1 | NM_127244.4 | s00789g23874.t1 | VIT_209s0002g02700.5 |
| 28 | s00010g01656.t1 | Fh.05G0005210 | s00789g23874.t1 | Fm.01G0017340 | s00016g02276.t1 | NM_105572.5 | s00055g05573.t1 | VIT_211s0016g03560.1 |
| 29 | s00010g01634.t1 | Fh.05G0005320 | s00010g01656.t1 | Fm.03G0040530 | s00780g23770.t2 | NM_001345225.1 | s00055g05573.t1 | VIT_209s0002g04120.1 |
| 30 | s00010g01594.t1 | Fh.05G0005690 | s00010g01656.t1 | Fm.05G0006300 | s01128g27016.t1 | NM_100795.4 | s00010g01656.t1 | VIT_218s0001g08040.1 |
| 31 | s00009g01513.t1 | Fh.07G0002620 | s00010g01594.t1 | Fm.03G0033980 | s01128g27016.t1 | NM_111454.3 | s00010g01634.t1 | VIT_207s0205g00160.2 |
| 32 | s00009g01513.t1 | Fh.05G0007280 | s00010g01634.t1 | Fm.05G0006160 | s00629g21808.t1 | NM_001342400.1 | s00010g01594.t1 | VIT_218s0001g08600.1 |
| 33 | s00009g01527.t1 | Fh.05G0007410 | s00009g01513.t1 | Fm.03G0032620 | s00307g15446.t1 | NM_001202598.1 | s00009g01513.t1 | VIT_218s0001g10270.1 |
| 34 | s00009g01527.t1 | Fh.07G0002720 | s00009g01513.t1 | Fm.05G0008650 | s00307g15446.t1 | NM_119546.3 | s00009g01513.t1 | VIT_207s0031g00450.1 |
| 35 | s00173g11418.t1 | Fh.01G0021630 | s00009g01527.t1 | Fm.03G0032510 | s01183g27416.t1 | NM_103870.3 | s00009g01527.t1 | VIT_218s0001g10400.1 |
| 36 | s00089g07669.t1 | Fh.10G0009820 | s00009g01527.t1 | Fm.05G0008490 | s01183g27416.t1 | NM_112837.3 | s00009g01527.t1 | VIT_207s0031g00550.1 |
| 37 | s00318g15757.t1 | Fh.05G0018850 | s00089g07669.t1 | Fm.10G0010620 | s00191g12059.t1 | NM_202643.2 | s00173g11418.t1 | VIT_205s0094g00480.3 |
| 38 | s00318g15757.t1 | Fh.01G0007750 | s00173g11418.t1 | Fm.13G0005670 | s00142g10133.t1 | NM_105318.3 | s00089g07669.t1 | VIT_203s0038g02540.1 |
| 39 | s00016g02276.t1 | Fh.11G0007560 | s00318g15757.t1 | Fm.01G0009020 | s00142g10133.t1 | NM_123139.2 | s00318g15757.t1 | VIT_210s0003g01160.1 |
| 40 | s00780g23770.t2 | Fh.09G0005550 | s00318g15757.t1 | Fm.05G0021570 | s00072g06736.t2 | NM_125656.3 | s00318g15757.t1 | VIT_212s0028g02350.1 |
| 41 | s01128g27016.t1 | Fh.03G0010240 | s00016g02276.t1 | Fm.11G0007870 | s00116g09023.t1 | NM_105555.4 | s00016g02276.t1 | VIT_201s0010g00540.3 |
| 42 | s00629g21808.t1 | Fh.10G0002440 | s01128g27016.t1 | Fm.03G0010640 | s00188g11941.t1 | NM_113470.3 | s00016g02276.t1 | VIT_217s0000g04790.1 |
| 43 | s00629g21808.t1 | Fh.05G0010830 | s00780g23770.t2 | Fm.09G0007300 | s00727g23102.t1 | NM_103864.2 | s00780g23770.t2 | VIT_211s0016g02070.1 |
| 44 | s00004g00755.t1 | Fh.05G0010070 | s00629g21808.t1 | Fm.05G0012810 | s00013g01919.t1 | NM_180832.3 | s01128g27016.t1 | VIT_214s0060g01010.1 |
| 45 | s00004g00755.t1 | Fh.07G0005070 | s00629g21808.t1 | Fm.10G0002640 | s00709g22875.t1 | NM_179498.3 | s00629g21808.t1 | VIT_204s0023g03430.1 |
| 46 | s00212g12766.t1 | Fh.07G0012950 | s00212g12766.t1 | Fm.03G0040530 | s00442g18537.t2 | NM_129578.4 | s00004g00755.t1 | VIT_218s0001g12970.2 |
| 47 | s00212g12766.t1 | Fh.05G0005210 | s00004g00755.t1 | Fm.05G0012030 | s00664g22314.t1 | NM_001342584.1 | s00004g00755.t1 | VIT_204s0023g02410.1 |
| 48 | s00583g21099.t1 | Fh.07G0013520 | s00212g12766.t1 | Fm.05G0006300 | s00007g01197.t1 | NM_125930.2 | s00212g12766.t1 | VIT_218s0001g08040.1 |
| 49 | s00240g13612.t1 | Fh.10G0017720 | s00583g21099.t1 | Fm.03G0041100 | s00053g05394.t1 | NM_001335659.1 | s00583g21099.t1 | VIT_218s0001g07410.1 |
| 50 | s00307g15446.t1 | Fh.10G0011830 | s00307g15446.t1 | Fm.03G0033430 | s00937g25469.t1 | NM_123247.5 | s00240g13612.t1 | VIT_218s0001g07200.1 |
| 51 | s00307g15446.t1 | Fh.07G0001820 | s00240g13612.t1 | Fm.03G0041340 | s00052g05353.t1 | NM_001203100.1 | s00307g15446.t1 | VIT_218s0001g09210.1 |
| 52 | s02722g31029.t1 | Fh.10G0011850 | s00307g15446.t1 | Fm.10G0012890 | s00268g14415.t1 | NM_102343.4 | s00307g15446.t1 | VIT_203s0091g00730.1 |
| 53 | s01183g27416.t1 | Fh.10G0012340 | s00307g15448.t1 | Fm.10G0012910 | s00340g16258.t1 | NM_127687.4 | s01183g27416.t1 | VIT_203s0091g00210.1 |
| 54 | s00191g12059.t1 | Fh.05G0012980 | s02722g31029.t1 | Fm.10G0012920 | s00050g05205.t1 | NM_001035697.2 | s00191g12059.t1 | VIT_214s0108g00420.1 |
| 55 | s00142g10133.t1 | Fh.05G0012330 | s01183g27416.t1 | Fm.10G0013550 | s00152g10604.t1 | NM_180037.4 | s00142g10133.t1 | VIT_214s0006g01470.1 |
| 56 | s00215g12870.t1 | Fh.09G0007050 | s00191g12059.t1 | Fm.05G0014980 | s00152g10604.t1 | NM_115642.2 | s00215g12870.t1 | VIT_211s0016g00380.3 |
| 57 | s02226g30702.t1 | Fh.06G0007440 | s00142g10133.t1 | Fm.05G0014530 | s00152g10599.t1 | NM_001336962.1 | s02226g30702.t1 | VIT_201s0127g00932.1 |
| 58 | s00072g06736.t2 | Fh.06G0008070 | s00215g12870.t1 | Fm.05G0000510 | s00725g23075.t1 | NM_001160722.2 | s02226g30702.t1 | VIT_217s0000g08150.1 |
| 59 | s00072g06736.t2 | Fh.11G0004630 | s02226g30702.t1 | Fm.05G0032410 | s01111g26902.t1 | NM_106043.3 | s00072g06736.t2 | VIT_201s0026g01140.1 |
| 60 | s00116g09023.t1 | Fh.11G0008370 | s02226g30702.t1 | Fm.11G0003420 | s00546g20503.t1 | NM_113319.5 | s00072g06736.t2 | VIT_217s0000g05370.1 |
| 61 | s00389g17429.t1 | Fh.04G0008110 | s00072g06736.t2 | Fm.11G0005090 | s00025g03213.t1 | NM_124039.3 | s00116g09023.t1 | VIT_201s0011g02940.2 |
| 62 | s00208g12624.t1 | Fh.05G0020020 | s00208g12624.t1 | Fm.01G0010260 | s00866g24700.t1 | NM_112586.3 | s00389g17429.t1 | VIT_213s0019g00540.1 |
| 63 | s00208g12624.t1 | Fh.01G0009050 | s00116g09023.t1 | Fm.11G0008680 | s00866g24700.t1 | NM_202510.1 | s00208g12624.t1 | VIT_212s0028g03550.1 |
| 64 | s00118g09121.t1 | Fh.05G0004130 | s00208g12624.t1 | Fm.05G0023510 | s00972g25791.t1 | NM_001342986.1 | s00234g13451.t1 | VIT_201s0244g00010.1 |
| 65 | s00234g13451.t1 | Fh.11G0000520 | s00118g09121.t1 | Fm.05G0005330 | s00147g10386.t1 | NM_102998.4 | s00234g13451.t1 | VIT_217s0000g00430.1 |
| 66 | s00727g23102.t1 | Fh.07G0002630 | s00234g13451.t1 | Fm.11G0000860 | s00147g10386.t1 | NM_117897.4 | s00727g23102.t1 | VIT_218s0001g10300.1 |
| 67 | s00013g01919.t1 | Fh.06G0003450 | s00727g23102.t1 | Fm.03G0032610 | s00171g11322.t1 | NM_001197954.2 | s00727g23102.t1 | VIT_203s0063g00170.1 |
| 68 | s00221g13054.t1 | Fh.11G0005530 | s00221g13054.t1 | Fm.06G0006380 | s00171g11322.t1 | NM_130216.3 | s00013g01919.t1 | VIT_217s0000g00430.1 |
| 69 | s00221g13054.t1 | Fh.06G0009080 | s00013g01919.t1 | Fm.11G0000860 | s00253g13975.t1 | NM_124849.4 | s00709g22875.t1 | VIT_217s0000g05370.1 |
| 70 | s00031g03756.t1 | Fh.06G0010000 | s00221g13054.t1 | Fm.11G0005810 | s00756g23470.t1 | NM_102804.2 | s00221g13054.t1 | VIT_201s0026g02030.1 |
| 71 | s00076g06984.t1 | Fh.03G0011070 | s00031g03756.t1 | Fm.06G0007490 | s01295g28048.t1 | NM_116060.3 | s00221g13054.t1 | VIT_214s0108g00420.1 |
| 72 | s00442g18537.t2 | Fh.04G0011130 | s00076g06984.t1 | Fm.03G0011500 | s00875g24796.t1 | NM_123731.5 | s00221g13054.t1 | VIT_217s0000g05900.1 |
| 73 | s00664g22314.t1 | Fh.12G0003570 | s00442g18537.t2 | Fm.04G0009160 | s00135g09872.t1 | NM_102735.3 | s00031g03756.t1 | VIT_217s0000g06930.2 |
| 74 | s00007g01197.t1 | Fh.07G0013990 | s00007g01197.t1 | Fm.03G0041640 |  |  | s00076g06984.t1 | VIT_214s0060g00260.3 |
| 75 | s00111g08792.t1 | Fh.05G0004510 | s00664g22314.t1 | Fm.12G0003200 |  |  | s00442g18537.t2 | VIT_213s0067g01350.1 |
| 76 | s00053g05394.t1 | Fh.09G0003490 | s00053g05394.t1 | Fm.01G0016500 |  |  | s00664g22314.t1 | VIT_208s0058g00110.1 |
| 77 | s00053g05394.t1 | Fh.01G0014850 | s00111g08792.t1 | Fm.05G0007080 |  |  | s00007g01197.t1 | VIT_218s0001g06650.1 |
| 78 | s00108g08612.t1 | Fh.05G0000240 | s00053g05394.t1 | Fm.09G0000830 |  |  | s00111g08792.t1 | VIT_207s0191g00240.1 |
| 79 | s00937g25469.t1 | Fh.05G0024530 | s00108g08612.t1 | Fm.05G0001510 |  |  | s00053g05394.t1 | VIT_211s0016g03560.1 |
| 80 | s00937g25469.t1 | Fh.11G0007560 | s00937g25469.t1 | Fm.05G0027650 |  |  | s00053g05394.t1 | VIT_209s0002g04120.1 |
| 81 | s00052g05353.t1 | Fh.06G0009160 | s00052g05353.t1 | Fm.06G0006460 |  |  | s00108g08612.t1 | VIT_204s0044g01040.1 |
| 82 | s00268g14415.t1 | Fh.06G0013610 | s00268g14415.t1 | Fm.06G0010620 |  |  | s00937g25469.t1 | VIT_201s0010g00540.3 |
| 83 | s00268g14415.t1 | Fh.11G0010560 | s00268g14415.t1 | Fm.11G0011860 |  |  | s00937g25469.t1 | VIT_214s0083g00930.1 |
| 84 | s00384g17303.t1 | Fh.05G0014010 | s00384g17303.t1 | Fm.05G0016380 |  |  | s00052g05353.t1 | VIT_214s0108g00480.1 |
| 85 | s00062g06080.t1 | Fh.10G0002240 | s00062g06080.t1 | Fm.10G0002380 |  |  | s00052g05353.t1 | VIT_217s0000g06000.1 |
| 86 | s00340g16258.t1 | Fh.10G0002430 | s00340g16258.t1 | Fm.10G0002630 |  |  | s00268g14415.t1 | VIT_201s0011g03720.1 |
| 87 | s00921g25318.t1 | Fh.01G0009050 | s00921g25318.t1 | Fm.01G0010260 |  |  | s00268g14415.t1 | VIT_214s0006g02850.1 |
| 88 | s00921g25318.t1 | Fh.05G0020020 | s00921g25318.t1 | Fm.05G0023510 |  |  | s00268g14415.t1 | VIT_217s0000g03580.1 |
| 89 | s00620g21689.t1 | Fh.12G0007560 | s01354g28377.t1 | Fm.08G0004370 |  |  | s00384g17303.t1 | VIT_214s0006g02850.1 |
| 90 | s00620g21689.t1 | Fh.07G0020650 | s00620g21689.t1 | Fm.12G0008130 |  |  | s00062g06080.t1 | VIT_203s0088g01240.1 |
| 91 | s01354g28377.t1 | Fh.08G0003680 | s00050g05205.t1 | Fm.05G0034280 |  |  | s00340g16258.t1 | VIT_203s0038g01780.1 |
| 92 | s00050g05205.t1 | Fh.05G0031240 | s00046g04922.t1 | Fm.09G0009180 |  |  | s00921g25318.t1 | VIT_212s0028g03550.1 |
| 93 | s00046g04922.t1 | Fh.12G0010890 | s00086g07547.t2 | Fm.03G0041340 |  |  | s01354g28377.t1 | VIT_205s0124g00240.1 |
| 94 | s00046g04922.t1 | Fh.09G0007950 | s00046g04922.t1 | Fm.12G0011270 |  |  | s00050g05205.t1 | VIT_214s0068g01200.1 |
| 95 | s00408g17845.t1 | Fh.03G0000950 | s00600g21369.t1 | Fm.05G0025220 |  |  | s00050g05205.t1 | VIT_217s0000g00330.1 |
| 96 | s00152g10604.t1 | Fh.13G0009550 | s01168g27316.t1 | Fm.05G0025090 |  |  | s00046g04922.t1 | VIT_215s0048g02820.1 |
| 97 | s00152g10599.t1 | Fh.13G0009590 | s00152g10604.t1 | Fm.13G0011180 |  |  | s00046g04922.t1 | VIT_202s0025g02610.1 |
| 98 | s00725g23075.t1 | Fh.12G0013580 | s00725g23075.t1 | Fm.09G0013300 |  |  | s00086g07547.t2 | VIT_218s0001g07200.1 |
| 99 | s00725g23075.t1 | Fh.08G0004580 | s00152g10599.t1 | Fm.13G0011230 |  |  | s01168g27316.t1 | VIT_212s0059g02650.1 |
| 100 | s00369g16979.t1 | Fh.05G0018850 | s00369g16979.t1 | Fm.05G0021570 |  |  | s01168g27316.t1 | VIT_219s0014g05100.1 |
| 101 | s01111g26902.t1 | Fh.05G0014010 | s01111g26902.t1 | Fm.06G0010620 |  |  | s00152g10604.t1 | VIT_208s0007g07870.1 |
| 102 | s01111g26902.t1 | Fh.11G0010560 | s00546g20503.t1 | Fm.08G0006850 |  |  | s00152g10599.t1 | VIT_213s0064g01290.1 |
| 103 | s01111g26902.t1 | Fh.06G0013610 | s01111g26902.t1 | Fm.11G0011860 |  |  | s00661g22283.t1 | VIT_208s0007g07810.1 |
| 104 | s00546g20503.t1 | Fh.08G0007170 | s00025g03213.t1 | Fm.12G0007990 |  |  | s00725g23075.t1 | VIT_215s0046g02560.1 |
| 105 | s00025g03213.t1 | Fh.12G0007400 | s01056g26517.t1 | Fm.10G0016640 |  |  | s00725g23075.t1 | VIT_202s0025g03450.2 |
| 106 | s01056g26517.t1 | Fh.10G0015150 | s00866g24700.t1 | Fm.08G0016060 |  |  | s00369g16979.t1 | VIT_210s0003g01160.1 |
| 107 | s00866g24700.t1 | Fh.08G0016690 | s00272g14504.t1 | Fm.11G0014460 |  |  | s00369g16979.t1 | VIT_212s0028g02350.1 |
| 108 | s00272g14504.t1 | Fh.11G0012700 | s00403g17722.t1 | Fm.02G0001240 |  |  | s01111g26902.t1 | VIT_201s0011g03720.1 |
| 109 | s00403g17722.t1 | Fh.02G0000710 | s00972g25791.t1 | Fm.11G0007870 |  |  | s01111g26902.t1 | VIT_214s0006g02850.1 |
| 110 | s00972g25791.t1 | Fh.06G0011480 | s00171g11322.t1 | Fm.09G0009680 |  |  | s01111g26902.t1 | VIT_217s0000g03580.1 |
| 111 | s00972g25791.t1 | Fh.05G0024530 | s00147g10386.t1 | Fm.12G0011270 |  |  | s00546g20503.t1 | VIT_205s0029g00050.1 |
| 112 | s00972g25791.t1 | Fh.11G0007560 | s00253g13975.t1 | Fm.12G0017320 |  |  | s00025g03213.t1 | VIT_202s0012g01450.1 |
| 113 | s00147g10386.t1 | Fh.12G0010890 | s00756g23470.t1 | Fm.01G0003540 |  |  | s01056g26517.t1 | VIT_203s0038g04760.1 |
| 114 | s00171g11322.t1 | Fh.09G0008380 | s01295g28048.t1 | Fm.09G0008850 |  |  | s00866g24700.t1 | VIT_205s0077g00750.1 |
| 115 | s00253g13975.t1 | Fh.14G0005460 | s00875g24796.t1 | Fm.08G0010340 |  |  | s00272g14504.t1 | VIT_201s0010g02070.1 |
| 116 | s01295g28048.t1 | Fh.09G0007630 | s00135g09872.t1 | Fm.01G0006730 |  |  | s00403g17722.t1 | VIT_213s0064g01290.1 |
| 117 | s00875g24796.t1 | Fh.08G0011160 | s00024g03087.t1 | Fm.02G0007830 |  |  | s00972g25791.t1 | VIT_214s0083g00930.1 |
| 118 | s00135g09872.t1 | Fh.01G0005300 | s00233g13419.t1 | Fm.02G0003520 |  |  | s00972g25791.t1 | VIT_217s0000g04790.1 |
| 119 | s00024g03087.t1 | Fh.02G0007580 | s00016g02259.t1 | Fm.08G0004500 |  |  | s00147g10386.t1 | VIT_202s0025g02610.1 |
| 120 | s00233g13419.t1 | Fh.02G0002920 |  |  |  |  | s00171g11322.t1 | VIT_215s0046g00320.1 |
| 121 | s00016g02259.t1 | Fh.08G0003820 |  |  |  |  | s00253g13975.t1 | VIT_219s0014g05100.1 |
| 122 |  |  |  |  |  |  | s00756g23470.t1 | VIT_210s0092g00030.1 |
| 123 |  |  |  |  |  |  | s01295g28048.t1 | VIT_215s0048g02510.2 |
| 124 |  |  |  |  |  |  | s00875g24796.t1 | VIT_205s0020g04780.1 |
| 125 |  |  |  |  |  |  | s00024g03087.t1 | VIT_211s0037g00040.1 |
| 126 |  |  |  |  |  |  | s00233g13419.t1 | VIT_211s0052g00100.1 |

**Table S7. FcbHLHs protein interaction score**

| *node1* | node2 | node1 accession | node2 accession | score |
| --- | --- | --- | --- | --- |
| *CKS2* | KRP2 | AT2G27970.1 | AT3G50630.1 | 0.995 |
| *CKS2* | s01354g28377.t1 | AT2G27970.1 | AT2G43140.2 | 0.995 |
| *CPC* | TTG1 | AT2G46410.1 | AT5G24520.1 | 0.952 |
| *CPC* | s00123g09328.t1 | AT2G46410.1 | AT4G33880.1 | 0.414 |
| *CPC* | s00142g10133.t1 | AT2G46410.1 | AT1G66470.1 | 0.567 |
| *CPC* | s00145g10283.t1 | AT2G46410.1 | AT4G09820.1 | 0.827 |
| *CPC* | s00725g23075.t1 | AT2G46410.1 | AT5G41315.1 | 0.993 |
| *HOS1* | PHYA | AT2G39810.1 | AT1G09570.1 | 0.463 |
| *HOS1* | PHYB | AT2G39810.1 | AT2G18790.1 | 0.887 |
| *HOS1* | s00050g05205.t1 | AT2G39810.1 | AT3G26744.1 | 0.993 |
| *HOS1* | s00097g08098.t1 | AT2G39810.1 | AT2G43010.1 | 0.463 |
| *JAZ1* | s00002g00351.t1 | AT1G19180.1 | AT4G16430.1 | 0.671 |
| *JAZ1* | s00050g05205.t1 | AT1G19180.1 | AT3G26744.1 | 0.753 |
| *JAZ1* | s00147g10386.t1 | AT1G19180.1 | AT4G00870.1 | 0.568 |
| *JAZ1* | s00171g11322.t1 | AT1G19180.1 | AT1G01260.1 | 0.883 |
| *JAZ1* | s00188g11941.t1 | AT1G19180.1 | AT2G41130.1 | 0.43 |
| *JAZ1* | s00233g13419.t1 | AT1G19180.1 | AT5G57150.4 | 0.622 |
| *JAZ1* | s00412g17915.t1 | AT1G19180.1 | AT1G32640.1 | 0.997 |
| *JAZ1* | s00725g23075.t1 | AT1G19180.1 | AT5G41315.1 | 0.464 |
| *JAZ1* | s00875g24796.t1 | AT1G19180.1 | AT5G43650.1 | 0.565 |
| *KRP2* | CKS2 | AT3G50630.1 | AT2G27970.1 | 0.995 |
| *KRP2* | s01354g28377.t1 | AT3G50630.1 | AT2G43140.2 | 0.995 |
| *MYB75* | TTG1 | AT1G56650.1 | AT5G24520.1 | 0.837 |
| *MYB75* | s00002g00351.t1 | AT1G56650.1 | AT4G16430.1 | 0.454 |
| *MYB75* | s00145g10283.t1 | AT1G56650.1 | AT4G09820.1 | 0.993 |
| *MYB75* | s00412g17915.t1 | AT1G56650.1 | AT1G32640.1 | 0.524 |
| *MYB75* | s00725g23075.t1 | AT1G56650.1 | AT5G41315.1 | 0.951 |
| *PHYA* | HOS1 | AT1G09570.1 | AT2G39810.1 | 0.463 |
| *PHYA* | PHYB | AT1G09570.1 | AT2G18790.1 | 0.781 |
| *PHYA* | s00013g01919.t1 | AT1G09570.1 | AT5G50915.1 | 0.746 |
| *PHYA* | s00069g06531.t1 | AT1G09570.1 | AT4G02590.1 | 0.533 |
| *PHYA* | s00076g06984.t1 | AT1G09570.1 | AT1G09530.2 | 0.998 |
| *PHYA* | s00097g08098.t1 | AT1G09570.1 | AT2G43010.1 | 0.923 |
| *PHYA* | s00184g11787.t1 | AT1G09570.1 | AT2G46970.1 | 0.72 |
| *PHYB* | HOS1 | AT2G18790.1 | AT2G39810.1 | 0.887 |
| *PHYB* | PHYA | AT2G18790.1 | AT1G09570.1 | 0.781 |
| *PHYB* | s00076g06984.t1 | AT2G18790.1 | AT1G09530.2 | 0.999 |
| *PHYB* | s00097g08098.t1 | AT2G18790.1 | AT2G43010.1 | 0.986 |
| *PHYB* | s00184g11787.t1 | AT2G18790.1 | AT2G46970.1 | 0.916 |
| *PHYB* | s00412g17915.t1 | AT2G18790.1 | AT1G32640.1 | 0.463 |
| *PHYB* | s00546g20503.t1 | AT2G18790.1 | AT3G24140.1 | 0.481 |
| *TT2* | TTG1 | AT5G35550.1 | AT5G24520.1 | 0.99 |
| *TT2* | s00002g00351.t1 | AT5G35550.1 | AT4G16430.1 | 0.525 |
| *TT2* | s00072g06736.t2 | AT5G35550.1 | AT5G62610.1 | 0.41 |
| *TT2* | s00145g10283.t1 | AT5G35550.1 | AT4G09820.1 | 0.994 |
| *TT2* | s00725g23075.t1 | AT5G35550.1 | AT5G41315.1 | 0.803 |
| *TTG1* | CPC | AT5G24520.1 | AT2G46410.1 | 0.952 |
| *TTG1* | MYB75 | AT5G24520.1 | AT1G56650.1 | 0.837 |
| *TTG1* | TT2 | AT5G24520.1 | AT5G35550.1 | 0.99 |
| *TTG1* | s00002g00351.t1 | AT5G24520.1 | AT4G16430.1 | 0.528 |
| *TTG1* | s00142g10133.t1 | AT5G24520.1 | AT1G66470.1 | 0.552 |
| *TTG1* | s00145g10283.t1 | AT5G24520.1 | AT4G09820.1 | 0.994 |
| *TTG1* | s00725g23075.t1 | AT5G24520.1 | AT5G41315.1 | 0.996 |
| *s00002g00351.t1* | JAZ1 | AT4G16430.1 | AT1G19180.1 | 0.671 |
| *s00002g00351.t1* | MYB75 | AT4G16430.1 | AT1G56650.1 | 0.454 |
| *s00002g00351.t1* | TT2 | AT4G16430.1 | AT5G35550.1 | 0.525 |
| *s00002g00351.t1* | TTG1 | AT4G16430.1 | AT5G24520.1 | 0.528 |
| *s00002g00351.t1* | s00025g03213.t1 | AT4G16430.1 | AT5G46690.1 | 0.439 |
| *s00002g00351.t1* | s00072g06736.t2 | AT4G16430.1 | AT5G62610.1 | 0.575 |
| *s00002g00351.t1* | s00307g15448.t1 | AT4G16430.1 | AT2G14760.3 | 0.408 |
| *s00003g00506.t1* | s00004g00755.t1 | AT2G28160.1 | AT1G27660.1 | 0.746 |
| *s00003g00506.t1* | s00052g05353.t1 | AT2G28160.1 | AT3G47640.2 | 0.837 |
| *s00003g00506.t1* | s00069g06531.t1 | AT2G28160.1 | AT4G02590.1 | 0.532 |
| *s00003g00506.t1* | s00123g09328.t1 | AT2G28160.1 | AT4G33880.1 | 0.506 |
| *s00003g00506.t1* | s00307g15448.t1 | AT2G28160.1 | AT2G14760.3 | 0.465 |
| *s00003g00506.t1* | s00780g23770.t2 | AT2G28160.1 | AT5G56960.1 | 0.586 |
| *s00003g00506.t1* | s00875g24796.t1 | AT2G28160.1 | AT5G43650.1 | 0.739 |
| *s00003g00506.t1* | s01168g27316.t1 | AT2G28160.1 | AT5G54680.1 | 0.629 |
| *s00004g00755.t1* | s00003g00506.t1 | AT1G27660.1 | AT2G28160.1 | 0.746 |
| *s00013g01919.t1* | PHYA | AT5G50915.1 | AT1G09570.1 | 0.746 |
| *s00013g01919.t1* | s00052g05353.t1 | AT5G50915.1 | AT3G47640.2 | 0.734 |
| *s00013g01919.t1* | s00173g11418.t1 | AT5G50915.1 | AT2G31730.1 | 0.57 |
| *s00013g01919.t1* | s00188g11941.t1 | AT5G50915.1 | AT2G41130.1 | 0.412 |
| *s00013g01919.t1* | s00272g14504.t1 | AT5G50915.1 | AT1G31050.1 | 0.56 |
| *s00013g01919.t1* | s00525g20136.t1 | AT5G50915.1 | AT5G53210.1 | 0.52 |
| *s00013g01919.t1* | s00725g23075.t1 | AT5G50915.1 | AT5G41315.1 | 0.451 |
| *s00013g01919.t1* | s00921g25318.t1 | AT5G50915.1 | AT4G20970.1 | 0.482 |
| *s00020g02758.t1* | s00020g02768.t1 | AT4G36930.1 | AT3G50330.1 | 0.777 |
| *s00020g02758.t1* | s00118g09121.t1 | AT4G36930.1 | AT5G09750.1 | 0.764 |
| *s00020g02758.t1* | s00173g11418.t1 | AT4G36930.1 | AT2G31730.1 | 0.465 |
| *s00020g02768.t1* | s00020g02758.t1 | AT3G50330.1 | AT4G36930.1 | 0.777 |
| *s00020g02768.t1* | s00025g03213.t1 | AT3G50330.1 | AT5G46690.1 | 0.571 |
| *s00020g02768.t1* | s00442g18537.t2 | AT3G50330.1 | AT2G40200.1 | 0.472 |
| *s00020g02768.t1* | s00723g23054.t1 | AT3G50330.1 | AT1G10610.1 | 0.574 |
| *s00024g03087.t1* | s00025g03213.t1 | AT2G24260.1 | AT5G46690.1 | 0.531 |
| *s00024g03087.t1* | s00086g07547.t2 | AT2G24260.1 | AT5G64340.1 | 0.527 |
| *s00024g03087.t1* | s00142g10133.t1 | AT2G24260.1 | AT1G66470.1 | 0.403 |
| *s00025g03213.t1* | s00002g00351.t1 | AT5G46690.1 | AT4G16430.1 | 0.439 |
| *s00025g03213.t1* | s00020g02768.t1 | AT5G46690.1 | AT3G50330.1 | 0.571 |
| *s00025g03213.t1* | s00024g03087.t1 | AT5G46690.1 | AT2G24260.1 | 0.531 |
| *s00025g03213.t1* | s00031g03756.t1 | AT5G46690.1 | AT4G00050.1 | 0.819 |
| *s00025g03213.t1* | s00052g05353.t1 | AT5G46690.1 | AT3G47640.2 | 0.735 |
| *s00025g03213.t1* | s00072g06736.t2 | AT5G46690.1 | AT5G62610.1 | 0.644 |
| *s00025g03213.t1* | s00086g07547.t2 | AT5G46690.1 | AT5G64340.1 | 0.555 |
| *s00025g03213.t1* | s00118g09121.t1 | AT5G46690.1 | AT5G09750.1 | 0.463 |
| *s00025g03213.t1* | s00123g09328.t1 | AT5G46690.1 | AT4G33880.1 | 0.465 |
| *s00025g03213.t1* | s00184g11787.t1 | AT5G46690.1 | AT2G46970.1 | 0.433 |
| *s00025g03213.t1* | s00235g13492.t1 | AT5G46690.1 | AT1G10120.1 | 0.587 |
| *s00025g03213.t1* | s00307g15448.t1 | AT5G46690.1 | AT2G14760.3 | 0.566 |
| *s00025g03213.t1* | s00312g15577.t1 | AT5G46690.1 | AT4G14410.1 | 0.499 |
| *s00025g03213.t1* | s00403g17722.t1 | AT5G46690.1 | AT2G42280.1 | 0.463 |
| *s00025g03213.t1* | s00408g17845.t1 | AT5G46690.1 | AT2G27230.2 | 0.45 |
| *s00025g03213.t1* | s00438g18457.t1 | AT5G46690.1 | AT4G21330.1 | 0.735 |
| *s00025g03213.t1* | s00442g18537.t2 | AT5G46690.1 | AT2G40200.1 | 0.623 |
| *s00025g03213.t1* | s00525g20136.t1 | AT5G46690.1 | AT5G53210.1 | 0.411 |
| *s00025g03213.t1* | s00546g20503.t1 | AT5G46690.1 | AT3G24140.1 | 0.83 |
| *s00025g03213.t1* | s00661g22283.t1 | AT5G46690.1 | AT1G35460.1 | 0.459 |
| *s00025g03213.t1* | s00709g22875.t1 | AT5G46690.1 | AT1G59640.2 | 0.644 |
| *s00025g03213.t1* | s00972g25791.t1 | AT5G46690.1 | AT1G69010.1 | 0.521 |
| *s00025g03213.t1* | s01111g26902.t1 | AT5G46690.1 | AT1G73830.1 | 0.448 |
| *s00031g03756.t1* | s00025g03213.t1 | AT4G00050.1 | AT5G46690.1 | 0.819 |
| *s00031g03756.t1* | s00052g05353.t1 | AT4G00050.1 | AT3G47640.2 | 0.735 |
| *s00031g03756.t1* | s00086g07547.t2 | AT4G00050.1 | AT5G64340.1 | 0.522 |
| *s00031g03756.t1* | s00147g10386.t1 | AT4G00050.1 | AT4G00870.1 | 0.569 |
| *s00031g03756.t1* | s00312g15577.t1 | AT4G00050.1 | AT4G14410.1 | 0.45 |
| *s00031g03756.t1* | s00438g18457.t1 | AT4G00050.1 | AT4G21330.1 | 0.737 |
| *s00031g03756.t1* | s00525g20136.t1 | AT4G00050.1 | AT5G53210.1 | 0.736 |
| *s00031g03756.t1* | s00546g20503.t1 | AT4G00050.1 | AT3G24140.1 | 0.808 |
| *s00031g03756.t1* | s01168g27316.t1 | AT4G00050.1 | AT5G54680.1 | 0.436 |
| *s00050g05205.t1* | HOS1 | AT3G26744.1 | AT2G39810.1 | 0.993 |
| *s00050g05205.t1* | JAZ1 | AT3G26744.1 | AT1G19180.1 | 0.753 |
| *s00050g05205.t1* | s00188g11941.t1 | AT3G26744.1 | AT2G41130.1 | 0.43 |
| *s00050g05205.t1* | s00525g20136.t1 | AT3G26744.1 | AT5G53210.1 | 0.958 |
| *s00050g05205.t1* | s00546g20503.t1 | AT3G26744.1 | AT3G24140.1 | 0.739 |
| *s00050g05205.t1* | s00727g23102.t1 | AT3G26744.1 | AT1G49770.1 | 0.889 |
| *s00050g05205.t1* | s00875g24796.t1 | AT3G26744.1 | AT5G43650.1 | 0.404 |
| *s00050g05205.t1* | s01168g27316.t1 | AT3G26744.1 | AT5G54680.1 | 0.53 |
| *s00052g05353.t1* | s00003g00506.t1 | AT3G47640.2 | AT2G28160.1 | 0.837 |
| *s00052g05353.t1* | s00013g01919.t1 | AT3G47640.2 | AT5G50915.1 | 0.734 |
| *s00052g05353.t1* | s00025g03213.t1 | AT3G47640.2 | AT5G46690.1 | 0.735 |
| *s00052g05353.t1* | s00031g03756.t1 | AT3G47640.2 | AT4G00050.1 | 0.735 |
| *s00052g05353.t1* | s00069g06531.t1 | AT3G47640.2 | AT4G02590.1 | 0.762 |
| *s00052g05353.t1* | s00072g06736.t2 | AT3G47640.2 | AT5G62610.1 | 0.485 |
| *s00052g05353.t1* | s00173g11418.t1 | AT3G47640.2 | AT2G31730.1 | 0.536 |
| *s00052g05353.t1* | s00212g12766.t1 | AT3G47640.2 | AT5G65640.1 | 0.502 |
| *s00052g05353.t1* | s00234g13451.t1 | AT3G47640.2 | AT5G48560.1 | 0.479 |
| *s00052g05353.t1* | s00235g13492.t1 | AT3G47640.2 | AT1G10120.1 | 0.499 |
| *s00052g05353.t1* | s00272g14504.t1 | AT3G47640.2 | AT1G31050.1 | 0.56 |
| *s00052g05353.t1* | s00312g15577.t1 | AT3G47640.2 | AT4G14410.1 | 0.711 |
| *s00052g05353.t1* | s00438g18457.t1 | AT3G47640.2 | AT4G21330.1 | 0.417 |
| *s00052g05353.t1* | s00525g20136.t1 | AT3G47640.2 | AT5G53210.1 | 0.543 |
| *s00052g05353.t1* | s00546g20503.t1 | AT3G47640.2 | AT3G24140.1 | 0.509 |
| *s00052g05353.t1* | s00709g22875.t1 | AT3G47640.2 | AT1G59640.2 | 0.546 |
| *s00052g05353.t1* | s01168g27316.t1 | AT3G47640.2 | AT5G54680.1 | 0.7 |
| *s00052g05353.t1* | s01183g27416.t1 | AT3G47640.2 | AT3G19500.1 | 0.641 |
| *s00052g05353.t1* | s01408g28647.t1 | AT3G47640.2 | AT3G07340.1 | 0.479 |
| *s00055g05573.t1* | s00583g21099.t1 | AT4G29100.1 | AT1G68810.1 | 0.57 |
| *s00062g06080.t1* | s00069g06531.t1 | AT4G37850.1 | AT4G02590.1 | 0.625 |
| *s00062g06080.t1* | s00307g15448.t1 | AT4G37850.1 | AT2G14760.3 | 0.528 |
| *s00062g06080.t1* | s00442g18537.t2 | AT4G37850.1 | AT2G40200.1 | 0.56 |
| *s00062g06080.t1* | s00583g21099.t1 | AT4G37850.1 | AT1G68810.1 | 0.53 |
| *s00069g06531.t1* | PHYA | AT4G02590.1 | AT1G09570.1 | 0.533 |
| *s00069g06531.t1* | s00003g00506.t1 | AT4G02590.1 | AT2G28160.1 | 0.532 |
| *s00069g06531.t1* | s00052g05353.t1 | AT4G02590.1 | AT3G47640.2 | 0.762 |
| *s00069g06531.t1* | s00062g06080.t1 | AT4G02590.1 | AT4G37850.1 | 0.625 |
| *s00069g06531.t1* | s00272g14504.t1 | AT4G02590.1 | AT1G31050.1 | 0.766 |
| *s00072g06736.t2* | TT2 | AT5G62610.1 | AT5G35550.1 | 0.41 |
| *s00072g06736.t2* | s00002g00351.t1 | AT5G62610.1 | AT4G16430.1 | 0.575 |
| *s00072g06736.t2* | s00025g03213.t1 | AT5G62610.1 | AT5G46690.1 | 0.644 |
| *s00072g06736.t2* | s00052g05353.t1 | AT5G62610.1 | AT3G47640.2 | 0.485 |
| *s00072g06736.t2* | s00111g08792.t1 | AT5G62610.1 | AT1G72210.1 | 0.564 |
| *s00072g06736.t2* | s00173g11418.t1 | AT5G62610.1 | AT2G31730.1 | 0.489 |
| *s00072g06736.t2* | s00256g14067.t1 | AT5G62610.1 | AT1G22490.1 | 0.564 |
| *s00072g06736.t2* | s00272g14504.t1 | AT5G62610.1 | AT1G31050.1 | 0.57 |
| *s00072g06736.t2* | s00525g20136.t1 | AT5G62610.1 | AT5G53210.1 | 0.44 |
| *s00072g06736.t2* | s00866g24700.t1 | AT5G62610.1 | AT3G17100.1 | 0.74 |
| *s00076g06984.t1* | PHYA | AT1G09530.2 | AT1G09570.1 | 0.998 |
| *s00076g06984.t1* | PHYB | AT1G09530.2 | AT2G18790.1 | 0.999 |
| *s00076g06984.t1* | s00097g08098.t1 | AT1G09530.2 | AT2G43010.1 | 0.965 |
| *s00076g06984.t1* | s00221g13054.t1 | AT1G09530.2 | AT1G26945.1 | 0.611 |
| *s00076g06984.t1* | s00525g20136.t1 | AT1G09530.2 | AT5G53210.1 | 0.405 |
| *s00076g06984.t1* | s01128g27016.t1 | AT1G09530.2 | AT1G09250.1 | 0.576 |
| *s00086g07547.t2* | s00024g03087.t1 | AT5G64340.1 | AT2G24260.1 | 0.527 |
| *s00086g07547.t2* | s00025g03213.t1 | AT5G64340.1 | AT5G46690.1 | 0.555 |
| *s00086g07547.t2* | s00031g03756.t1 | AT5G64340.1 | AT4G00050.1 | 0.522 |
| *s00086g07547.t2* | s00135g09872.t1 | AT5G64340.1 | AT1G29950.2 | 0.605 |
| *s00086g07547.t2* | s00307g15448.t1 | AT5G64340.1 | AT2G14760.3 | 0.465 |
| *s00086g07547.t2* | s00408g17845.t1 | AT5G64340.1 | AT2G27230.2 | 0.751 |
| *s00086g07547.t2* | s00442g18537.t2 | AT5G64340.1 | AT2G40200.1 | 0.53 |
| *s00086g07547.t2* | s00727g23102.t1 | AT5G64340.1 | AT1G49770.1 | 0.431 |
| *s00086g07547.t2* | s01111g26902.t1 | AT5G64340.1 | AT1G73830.1 | 0.43 |
| *s00089g07669.t1* | s00438g18457.t1 | AT2G16910.1 | AT4G21330.1 | 0.808 |
| *s00089g07669.t1* | s00620g21689.t1 | AT2G16910.1 | AT2G31220.1 | 0.412 |
| *s00097g08098.t1* | HOS1 | AT2G43010.1 | AT2G39810.1 | 0.463 |
| *s00097g08098.t1* | PHYA | AT2G43010.1 | AT1G09570.1 | 0.923 |
| *s00097g08098.t1* | PHYB | AT2G43010.1 | AT2G18790.1 | 0.986 |
| *s00097g08098.t1* | s00076g06984.t1 | AT2G43010.1 | AT1G09530.2 | 0.965 |
| *s00097g08098.t1* | s00221g13054.t1 | AT2G43010.1 | AT1G26945.1 | 0.664 |
| *s00111g08792.t1* | s00072g06736.t2 | AT1G72210.1 | AT5G62610.1 | 0.564 |
| *s00111g08792.t1* | s00135g09872.t1 | AT1G72210.1 | AT1G29950.2 | 0.692 |
| *s00111g08792.t1* | s00235g13492.t1 | AT1G72210.1 | AT1G10120.1 | 0.475 |
| *s00111g08792.t1* | s00272g14504.t1 | AT1G72210.1 | AT1G31050.1 | 0.4 |
| *s00111g08792.t1* | s00709g22875.t1 | AT1G72210.1 | AT1G59640.2 | 0.564 |
| *s00111g08792.t1* | s00875g24796.t1 | AT1G72210.1 | AT5G43650.1 | 0.4 |
| *s00111g08792.t1* | s00972g25791.t1 | AT1G72210.1 | AT1G69010.1 | 0.499 |
| *s00118g09121.t1* | s00020g02758.t1 | AT5G09750.1 | AT4G36930.1 | 0.764 |
| *s00118g09121.t1* | s00025g03213.t1 | AT5G09750.1 | AT5G46690.1 | 0.463 |
| *s00118g09121.t1* | s00442g18537.t2 | AT5G09750.1 | AT2G40200.1 | 0.626 |
| *s00123g09328.t1* | CPC | AT4G33880.1 | AT2G46410.1 | 0.414 |
| *s00123g09328.t1* | s00003g00506.t1 | AT4G33880.1 | AT2G28160.1 | 0.506 |
| *s00123g09328.t1* | s00025g03213.t1 | AT4G33880.1 | AT5G46690.1 | 0.465 |
| *s00123g09328.t1* | s00142g10133.t1 | AT4G33880.1 | AT1G66470.1 | 0.564 |
| *s00123g09328.t1* | s00215g12870.t1 | AT4G33880.1 | AT5G58010.1 | 0.551 |
| *s00123g09328.t1* | s00442g18537.t2 | AT4G33880.1 | AT2G40200.1 | 0.701 |
| *s00123g09328.t1* | s00725g23075.t1 | AT4G33880.1 | AT5G41315.1 | 0.499 |
| *s00123g09328.t1* | s00727g23102.t1 | AT4G33880.1 | AT1G49770.1 | 0.653 |
| *s00135g09872.t1* | s00086g07547.t2 | AT1G29950.2 | AT5G64340.1 | 0.605 |
| *s00135g09872.t1* | s00111g08792.t1 | AT1G29950.2 | AT1G72210.1 | 0.692 |
| *s00135g09872.t1* | s00240g13612.t1 | AT1G29950.2 | AT5G50010.1 | 0.443 |
| *s00135g09872.t1* | s00972g25791.t1 | AT1G29950.2 | AT1G69010.1 | 0.567 |
| *s00142g10133.t1* | CPC | AT1G66470.1 | AT2G46410.1 | 0.567 |
| *s00142g10133.t1* | TTG1 | AT1G66470.1 | AT5G24520.1 | 0.552 |
| *s00142g10133.t1* | s00024g03087.t1 | AT1G66470.1 | AT2G24260.1 | 0.403 |
| *s00142g10133.t1* | s00123g09328.t1 | AT1G66470.1 | AT4G33880.1 | 0.564 |
| *s00142g10133.t1* | s00215g12870.t1 | AT1G66470.1 | AT5G58010.1 | 0.574 |
| *s00142g10133.t1* | s00272g14504.t1 | AT1G66470.1 | AT1G31050.1 | 0.597 |
| *s00142g10133.t1* | s00408g17845.t1 | AT1G66470.1 | AT2G27230.2 | 0.41 |
| *s00142g10133.t1* | s00583g21099.t1 | AT1G66470.1 | AT1G68810.1 | 0.416 |
| *s00142g10133.t1* | s00725g23075.t1 | AT1G66470.1 | AT5G41315.1 | 0.638 |
| *s00142g10133.t1* | s00786g23832.t1 | AT1G66470.1 | AT4G25400.1 | 0.424 |
| *s00145g10283.t1* | CPC | AT4G09820.1 | AT2G46410.1 | 0.827 |
| *s00145g10283.t1* | MYB75 | AT4G09820.1 | AT1G56650.1 | 0.993 |
| *s00145g10283.t1* | TT2 | AT4G09820.1 | AT5G35550.1 | 0.994 |
| *s00145g10283.t1* | TTG1 | AT4G09820.1 | AT5G24520.1 | 0.994 |
| *s00147g10386.t1* | JAZ1 | AT4G00870.1 | AT1G19180.1 | 0.568 |
| *s00147g10386.t1* | s00031g03756.t1 | AT4G00870.1 | AT4G00050.1 | 0.569 |
| *s00152g10604.t1* | s00171g11322.t1 | AT3G57800.1 | AT1G01260.1 | 0.647 |
| *s00152g10604.t1* | s00233g13419.t1 | AT3G57800.1 | AT5G57150.4 | 0.676 |
| *s00171g11322.t1* | JAZ1 | AT1G01260.1 | AT1G19180.1 | 0.883 |
| *s00171g11322.t1* | s00152g10604.t1 | AT1G01260.1 | AT3G57800.1 | 0.647 |
| *s00171g11322.t1* | s00188g11941.t1 | AT1G01260.1 | AT2G41130.1 | 0.499 |
| *s00171g11322.t1* | s00403g17722.t1 | AT1G01260.1 | AT2G42280.1 | 0.43 |
| *s00171g11322.t1* | s01056g26517.t1 | AT1G01260.1 | AT1G68920.1 | 0.416 |
| *s00171g11322.t1* | s01128g27016.t1 | AT1G01260.1 | AT1G09250.1 | 0.526 |
| *s00171g11322.t1* | s01354g28377.t1 | AT1G01260.1 | AT2G43140.2 | 0.61 |
| *s00173g11418.t1* | s00013g01919.t1 | AT2G31730.1 | AT5G50915.1 | 0.57 |
| *s00173g11418.t1* | s00020g02758.t1 | AT2G31730.1 | AT4G36930.1 | 0.465 |
| *s00173g11418.t1* | s00052g05353.t1 | AT2G31730.1 | AT3G47640.2 | 0.536 |
| *s00173g11418.t1* | s00072g06736.t2 | AT2G31730.1 | AT5G62610.1 | 0.489 |
| *s00173g11418.t1* | s00234g13451.t1 | AT2G31730.1 | AT5G48560.1 | 0.437 |
| *s00173g11418.t1* | s00235g13492.t1 | AT2G31730.1 | AT1G10120.1 | 0.473 |
| *s00173g11418.t1* | s00525g20136.t1 | AT2G31730.1 | AT5G53210.1 | 0.44 |
| *s00173g11418.t1* | s00709g22875.t1 | AT2G31730.1 | AT1G59640.2 | 0.489 |
| *s00173g11418.t1* | s01408g28647.t1 | AT2G31730.1 | AT3G07340.1 | 0.437 |
| *s00184g11787.t1* | PHYA | AT2G46970.1 | AT1G09570.1 | 0.72 |
| *s00184g11787.t1* | PHYB | AT2G46970.1 | AT2G18790.1 | 0.916 |
| *s00184g11787.t1* | s00025g03213.t1 | AT2G46970.1 | AT5G46690.1 | 0.433 |
| *s00184g11787.t1* | s00221g13054.t1 | AT2G46970.1 | AT1G26945.1 | 0.416 |
| *s00184g11787.t1* | s00780g23770.t2 | AT2G46970.1 | AT5G56960.1 | 0.497 |
| *s00184g11787.t1* | s00789g23874.t1 | AT2G46970.1 | AT3G21330.1 | 0.432 |
| *s00184g11787.t1* | s01128g27016.t1 | AT2G46970.1 | AT1G09250.1 | 0.576 |
| *s00188g11941.t1* | JAZ1 | AT2G41130.1 | AT1G19180.1 | 0.43 |
| *s00188g11941.t1* | s00013g01919.t1 | AT2G41130.1 | AT5G50915.1 | 0.412 |
| *s00188g11941.t1* | s00050g05205.t1 | AT2G41130.1 | AT3G26744.1 | 0.43 |
| *s00188g11941.t1* | s00171g11322.t1 | AT2G41130.1 | AT1G01260.1 | 0.499 |
| *s00188g11941.t1* | s00233g13419.t1 | AT2G41130.1 | AT5G57150.4 | 0.545 |
| *s00188g11941.t1* | s00789g23874.t1 | AT2G41130.1 | AT3G21330.1 | 0.531 |
| *s00212g12766.t1* | s00052g05353.t1 | AT5G65640.1 | AT3G47640.2 | 0.502 |
| *s00212g12766.t1* | s00221g13054.t1 | AT5G65640.1 | AT1G26945.1 | 0.533 |
| *s00212g12766.t1* | s00525g20136.t1 | AT5G65640.1 | AT5G53210.1 | 0.4 |
| *s00212g12766.t1* | s00546g20503.t1 | AT5G65640.1 | AT3G24140.1 | 0.82 |
| *s00212g12766.t1* | s01408g28647.t1 | AT5G65640.1 | AT3G07340.1 | 0.439 |
| *s00215g12870.t1* | s00123g09328.t1 | AT5G58010.1 | AT4G33880.1 | 0.551 |
| *s00215g12870.t1* | s00142g10133.t1 | AT5G58010.1 | AT1G66470.1 | 0.574 |
| *s00215g12870.t1* | s00786g23832.t1 | AT5G58010.1 | AT4G25400.1 | 0.422 |
| *s00221g13054.t1* | s00076g06984.t1 | AT1G26945.1 | AT1G09530.2 | 0.611 |
| *s00221g13054.t1* | s00097g08098.t1 | AT1G26945.1 | AT2G43010.1 | 0.664 |
| *s00221g13054.t1* | s00184g11787.t1 | AT1G26945.1 | AT2G46970.1 | 0.416 |
| *s00221g13054.t1* | s00212g12766.t1 | AT1G26945.1 | AT5G65640.1 | 0.533 |
| *s00221g13054.t1* | s00235g13492.t1 | AT1G26945.1 | AT1G10120.1 | 0.526 |
| *s00221g13054.t1* | s00408g17845.t1 | AT1G26945.1 | AT2G27230.2 | 0.529 |
| *s00221g13054.t1* | s00866g24700.t1 | AT1G26945.1 | AT3G17100.1 | 0.535 |
| *s00221g13054.t1* | s01056g26517.t1 | AT1G26945.1 | AT1G68920.1 | 0.465 |
| *s00233g13419.t1* | JAZ1 | AT5G57150.4 | AT1G19180.1 | 0.622 |
| *s00233g13419.t1* | s00152g10604.t1 | AT5G57150.4 | AT3G57800.1 | 0.676 |
| *s00233g13419.t1* | s00188g11941.t1 | AT5G57150.4 | AT2G41130.1 | 0.545 |
| *s00233g13419.t1* | s00620g21689.t1 | AT5G57150.4 | AT2G31220.1 | 0.44 |
| *s00233g13419.t1* | s00786g23832.t1 | AT5G57150.4 | AT4G25400.1 | 0.577 |
| *s00233g13419.t1* | s00866g24700.t1 | AT5G57150.4 | AT3G17100.1 | 0.441 |
| *s00233g13419.t1* | s00875g24796.t1 | AT5G57150.4 | AT5G43650.1 | 0.967 |
| *s00233g13419.t1* | s00972g25791.t1 | AT5G57150.4 | AT1G69010.1 | 0.402 |
| *s00233g13419.t1* | s01056g26517.t1 | AT5G57150.4 | AT1G68920.1 | 0.439 |
| *s00233g13419.t1* | s01128g27016.t1 | AT5G57150.4 | AT1G09250.1 | 0.587 |
| *s00234g13451.t1* | s00052g05353.t1 | AT5G48560.1 | AT3G47640.2 | 0.479 |
| *s00234g13451.t1* | s00173g11418.t1 | AT5G48560.1 | AT2G31730.1 | 0.437 |
| *s00234g13451.t1* | s00272g14504.t1 | AT5G48560.1 | AT1G31050.1 | 0.558 |
| *s00235g13492.t1* | s00025g03213.t1 | AT1G10120.1 | AT5G46690.1 | 0.587 |
| *s00235g13492.t1* | s00052g05353.t1 | AT1G10120.1 | AT3G47640.2 | 0.499 |
| *s00235g13492.t1* | s00111g08792.t1 | AT1G10120.1 | AT1G72210.1 | 0.475 |
| *s00235g13492.t1* | s00173g11418.t1 | AT1G10120.1 | AT2G31730.1 | 0.473 |
| *s00235g13492.t1* | s00221g13054.t1 | AT1G10120.1 | AT1G26945.1 | 0.526 |
| *s00235g13492.t1* | s00256g14067.t1 | AT1G10120.1 | AT1G22490.1 | 0.475 |
| *s00235g13492.t1* | s00272g14504.t1 | AT1G10120.1 | AT1G31050.1 | 0.561 |
| *s00235g13492.t1* | s00525g20136.t1 | AT1G10120.1 | AT5G53210.1 | 0.468 |
| *s00240g13612.t1* | s00135g09872.t1 | AT5G50010.1 | AT1G29950.2 | 0.443 |
| *s00256g14067.t1* | s00072g06736.t2 | AT1G22490.1 | AT5G62610.1 | 0.564 |
| *s00256g14067.t1* | s00235g13492.t1 | AT1G22490.1 | AT1G10120.1 | 0.475 |
| *s00256g14067.t1* | s00272g14504.t1 | AT1G22490.1 | AT1G31050.1 | 0.4 |
| *s00256g14067.t1* | s00709g22875.t1 | AT1G22490.1 | AT1G59640.2 | 0.564 |
| *s00256g14067.t1* | s00875g24796.t1 | AT1G22490.1 | AT5G43650.1 | 0.4 |
| *s00268g14415.t1* | s01111g26902.t1 | AT1G25330.1 | AT1G73830.1 | 0.77 |
| *s00272g14504.t1* | s00013g01919.t1 | AT1G31050.1 | AT5G50915.1 | 0.56 |
| *s00272g14504.t1* | s00052g05353.t1 | AT1G31050.1 | AT3G47640.2 | 0.56 |
| *s00272g14504.t1* | s00069g06531.t1 | AT1G31050.1 | AT4G02590.1 | 0.766 |
| *s00272g14504.t1* | s00072g06736.t2 | AT1G31050.1 | AT5G62610.1 | 0.57 |
| *s00272g14504.t1* | s00111g08792.t1 | AT1G31050.1 | AT1G72210.1 | 0.4 |
| *s00272g14504.t1* | s00142g10133.t1 | AT1G31050.1 | AT1G66470.1 | 0.597 |
| *s00272g14504.t1* | s00234g13451.t1 | AT1G31050.1 | AT5G48560.1 | 0.558 |
| *s00272g14504.t1* | s00235g13492.t1 | AT1G31050.1 | AT1G10120.1 | 0.561 |
| *s00272g14504.t1* | s00256g14067.t1 | AT1G31050.1 | AT1G22490.1 | 0.4 |
| *s00272g14504.t1* | s00525g20136.t1 | AT1G31050.1 | AT5G53210.1 | 0.436 |
| *s00272g14504.t1* | s00583g21099.t1 | AT1G31050.1 | AT1G68810.1 | 0.713 |
| *s00272g14504.t1* | s00709g22875.t1 | AT1G31050.1 | AT1G59640.2 | 0.57 |
| *s00272g14504.t1* | s01408g28647.t1 | AT1G31050.1 | AT3G07340.1 | 0.521 |
| *s00307g15448.t1* | s00002g00351.t1 | AT2G14760.3 | AT4G16430.1 | 0.408 |
| *s00307g15448.t1* | s00003g00506.t1 | AT2G14760.3 | AT2G28160.1 | 0.465 |
| *s00307g15448.t1* | s00025g03213.t1 | AT2G14760.3 | AT5G46690.1 | 0.566 |
| *s00307g15448.t1* | s00062g06080.t1 | AT2G14760.3 | AT4G37850.1 | 0.528 |
| *s00307g15448.t1* | s00086g07547.t2 | AT2G14760.3 | AT5G64340.1 | 0.465 |
| *s00307g15448.t1* | s00312g15577.t1 | AT2G14760.3 | AT4G14410.1 | 0.418 |
| *s00307g15448.t1* | s00442g18537.t2 | AT2G14760.3 | AT2G40200.1 | 0.626 |
| *s00307g15448.t1* | s00525g20136.t1 | AT2G14760.3 | AT5G53210.1 | 0.435 |
| *s00307g15448.t1* | s00583g21099.t1 | AT2G14760.3 | AT1G68810.1 | 0.529 |
| *s00307g15448.t1* | s00725g23075.t1 | AT2G14760.3 | AT5G41315.1 | 0.439 |
| *s00307g15448.t1* | s00727g23102.t1 | AT2G14760.3 | AT1G49770.1 | 0.653 |
| *s00312g15577.t1* | s00025g03213.t1 | AT4G14410.1 | AT5G46690.1 | 0.499 |
| *s00312g15577.t1* | s00031g03756.t1 | AT4G14410.1 | AT4G00050.1 | 0.45 |
| *s00312g15577.t1* | s00052g05353.t1 | AT4G14410.1 | AT3G47640.2 | 0.711 |
| *s00312g15577.t1* | s00307g15448.t1 | AT4G14410.1 | AT2G14760.3 | 0.418 |
| *s00312g15577.t1* | s00442g18537.t2 | AT4G14410.1 | AT2G40200.1 | 0.465 |
| *s00312g15577.t1* | s01168g27316.t1 | AT4G14410.1 | AT5G54680.1 | 0.694 |
| *s00403g17722.t1* | s00025g03213.t1 | AT2G42280.1 | AT5G46690.1 | 0.463 |
| *s00403g17722.t1* | s00171g11322.t1 | AT2G42280.1 | AT1G01260.1 | 0.43 |
| *s00403g17722.t1* | s00442g18537.t2 | AT2G42280.1 | AT2G40200.1 | 0.456 |
| *s00403g17722.t1* | s00723g23054.t1 | AT2G42280.1 | AT1G10610.1 | 0.438 |
| *s00403g17722.t1* | s01128g27016.t1 | AT2G42280.1 | AT1G09250.1 | 0.465 |
| *s00408g17845.t1* | s00025g03213.t1 | AT2G27230.2 | AT5G46690.1 | 0.45 |
| *s00408g17845.t1* | s00086g07547.t2 | AT2G27230.2 | AT5G64340.1 | 0.751 |
| *s00408g17845.t1* | s00142g10133.t1 | AT2G27230.2 | AT1G66470.1 | 0.41 |
| *s00408g17845.t1* | s00221g13054.t1 | AT2G27230.2 | AT1G26945.1 | 0.529 |
| *s00408g17845.t1* | s00442g18537.t2 | AT2G27230.2 | AT2G40200.1 | 0.431 |
| *s00408g17845.t1* | s00583g21099.t1 | AT2G27230.2 | AT1G68810.1 | 0.864 |
| *s00408g17845.t1* | s00875g24796.t1 | AT2G27230.2 | AT5G43650.1 | 0.552 |
| *s00412g17915.t1* | JAZ1 | AT1G32640.1 | AT1G19180.1 | 0.997 |
| *s00412g17915.t1* | MYB75 | AT1G32640.1 | AT1G56650.1 | 0.524 |
| *s00412g17915.t1* | PHYB | AT1G32640.1 | AT2G18790.1 | 0.463 |
| *s00438g18457.t1* | s00025g03213.t1 | AT4G21330.1 | AT5G46690.1 | 0.735 |
| *s00438g18457.t1* | s00031g03756.t1 | AT4G21330.1 | AT4G00050.1 | 0.737 |
| *s00438g18457.t1* | s00052g05353.t1 | AT4G21330.1 | AT3G47640.2 | 0.417 |
| *s00438g18457.t1* | s00089g07669.t1 | AT4G21330.1 | AT2G16910.1 | 0.808 |
| *s00438g18457.t1* | s00525g20136.t1 | AT4G21330.1 | AT5G53210.1 | 0.605 |
| *s00438g18457.t1* | s00546g20503.t1 | AT4G21330.1 | AT3G24140.1 | 0.679 |
| *s00438g18457.t1* | s00620g21689.t1 | AT4G21330.1 | AT2G31220.1 | 0.918 |
| *s00438g18457.t1* | s01295g28048.t1 | AT4G21330.1 | AT2G46810.1 | 0.476 |
| *s00442g18537.t2* | s00020g02768.t1 | AT2G40200.1 | AT3G50330.1 | 0.472 |
| *s00442g18537.t2* | s00025g03213.t1 | AT2G40200.1 | AT5G46690.1 | 0.623 |
| *s00442g18537.t2* | s00062g06080.t1 | AT2G40200.1 | AT4G37850.1 | 0.56 |
| *s00442g18537.t2* | s00086g07547.t2 | AT2G40200.1 | AT5G64340.1 | 0.53 |
| *s00442g18537.t2* | s00118g09121.t1 | AT2G40200.1 | AT5G09750.1 | 0.626 |
| *s00442g18537.t2* | s00123g09328.t1 | AT2G40200.1 | AT4G33880.1 | 0.701 |
| *s00442g18537.t2* | s00307g15448.t1 | AT2G40200.1 | AT2G14760.3 | 0.626 |
| *s00442g18537.t2* | s00312g15577.t1 | AT2G40200.1 | AT4G14410.1 | 0.465 |
| *s00442g18537.t2* | s00403g17722.t1 | AT2G40200.1 | AT2G42280.1 | 0.456 |
| *s00442g18537.t2* | s00408g17845.t1 | AT2G40200.1 | AT2G27230.2 | 0.431 |
| *s00442g18537.t2* | s00525g20136.t1 | AT2G40200.1 | AT5G53210.1 | 0.617 |
| *s00442g18537.t2* | s00661g22283.t1 | AT2G40200.1 | AT1G35460.1 | 0.436 |
| *s00442g18537.t2* | s00972g25791.t1 | AT2G40200.1 | AT1G69010.1 | 0.52 |
| *s00442g18537.t2* | s01111g26902.t1 | AT2G40200.1 | AT1G73830.1 | 0.612 |
| *s00525g20136.t1* | s00013g01919.t1 | AT5G53210.1 | AT5G50915.1 | 0.52 |
| *s00525g20136.t1* | s00025g03213.t1 | AT5G53210.1 | AT5G46690.1 | 0.411 |
| *s00525g20136.t1* | s00031g03756.t1 | AT5G53210.1 | AT4G00050.1 | 0.736 |
| *s00525g20136.t1* | s00050g05205.t1 | AT5G53210.1 | AT3G26744.1 | 0.958 |
| *s00525g20136.t1* | s00052g05353.t1 | AT5G53210.1 | AT3G47640.2 | 0.543 |
| *s00525g20136.t1* | s00072g06736.t2 | AT5G53210.1 | AT5G62610.1 | 0.44 |
| *s00525g20136.t1* | s00076g06984.t1 | AT5G53210.1 | AT1G09530.2 | 0.405 |
| *s00525g20136.t1* | s00173g11418.t1 | AT5G53210.1 | AT2G31730.1 | 0.44 |
| *s00525g20136.t1* | s00212g12766.t1 | AT5G53210.1 | AT5G65640.1 | 0.4 |
| *s00525g20136.t1* | s00235g13492.t1 | AT5G53210.1 | AT1G10120.1 | 0.468 |
| *s00525g20136.t1* | s00272g14504.t1 | AT5G53210.1 | AT1G31050.1 | 0.436 |
| *s00525g20136.t1* | s00307g15448.t1 | AT5G53210.1 | AT2G14760.3 | 0.435 |
| *s00525g20136.t1* | s00438g18457.t1 | AT5G53210.1 | AT4G21330.1 | 0.605 |
| *s00525g20136.t1* | s00442g18537.t2 | AT5G53210.1 | AT2G40200.1 | 0.617 |
| *s00525g20136.t1* | s00709g22875.t1 | AT5G53210.1 | AT1G59640.2 | 0.44 |
| *s00546g20503.t1* | PHYB | AT3G24140.1 | AT2G18790.1 | 0.481 |
| *s00546g20503.t1* | s00025g03213.t1 | AT3G24140.1 | AT5G46690.1 | 0.83 |
| *s00546g20503.t1* | s00031g03756.t1 | AT3G24140.1 | AT4G00050.1 | 0.808 |
| *s00546g20503.t1* | s00050g05205.t1 | AT3G24140.1 | AT3G26744.1 | 0.739 |
| *s00546g20503.t1* | s00052g05353.t1 | AT3G24140.1 | AT3G47640.2 | 0.509 |
| *s00546g20503.t1* | s00212g12766.t1 | AT3G24140.1 | AT5G65640.1 | 0.82 |
| *s00546g20503.t1* | s00438g18457.t1 | AT3G24140.1 | AT4G21330.1 | 0.679 |
| *s00546g20503.t1* | s01168g27316.t1 | AT3G24140.1 | AT5G54680.1 | 0.41 |
| *s00583g21099.t1* | s00055g05573.t1 | AT1G68810.1 | AT4G29100.1 | 0.57 |
| *s00583g21099.t1* | s00062g06080.t1 | AT1G68810.1 | AT4G37850.1 | 0.53 |
| *s00583g21099.t1* | s00142g10133.t1 | AT1G68810.1 | AT1G66470.1 | 0.416 |
| *s00583g21099.t1* | s00272g14504.t1 | AT1G68810.1 | AT1G31050.1 | 0.713 |
| *s00583g21099.t1* | s00307g15448.t1 | AT1G68810.1 | AT2G14760.3 | 0.529 |
| *s00583g21099.t1* | s00408g17845.t1 | AT1G68810.1 | AT2G27230.2 | 0.864 |
| *s00583g21099.t1* | s00620g21689.t1 | AT1G68810.1 | AT2G31220.1 | 0.68 |
| *s00583g21099.t1* | s01295g28048.t1 | AT1G68810.1 | AT2G46810.1 | 0.626 |
| *s00620g21689.t1* | s00089g07669.t1 | AT2G31220.1 | AT2G16910.1 | 0.412 |
| *s00620g21689.t1* | s00233g13419.t1 | AT2G31220.1 | AT5G57150.4 | 0.44 |
| *s00620g21689.t1* | s00438g18457.t1 | AT2G31220.1 | AT4G21330.1 | 0.918 |
| *s00620g21689.t1* | s00583g21099.t1 | AT2G31220.1 | AT1G68810.1 | 0.68 |
| *s00620g21689.t1* | s00727g23102.t1 | AT2G31220.1 | AT1G49770.1 | 0.617 |
| *s00620g21689.t1* | s00780g23770.t2 | AT2G31220.1 | AT5G56960.1 | 0.831 |
| *s00620g21689.t1* | s00875g24796.t1 | AT2G31220.1 | AT5G43650.1 | 0.526 |
| *s00620g21689.t1* | s01295g28048.t1 | AT2G31220.1 | AT2G46810.1 | 0.782 |
| *s00661g22283.t1* | s00025g03213.t1 | AT1G35460.1 | AT5G46690.1 | 0.459 |
| *s00661g22283.t1* | s00442g18537.t2 | AT1G35460.1 | AT2G40200.1 | 0.436 |
| *s00664g22314.t1* | s00723g23054.t1 | AT5G01310.1 | AT1G10610.1 | 0.699 |
| *s00709g22875.t1* | s00025g03213.t1 | AT1G59640.2 | AT5G46690.1 | 0.644 |
| *s00709g22875.t1* | s00052g05353.t1 | AT1G59640.2 | AT3G47640.2 | 0.546 |
| *s00709g22875.t1* | s00111g08792.t1 | AT1G59640.2 | AT1G72210.1 | 0.564 |
| *s00709g22875.t1* | s00173g11418.t1 | AT1G59640.2 | AT2G31730.1 | 0.489 |
| *s00709g22875.t1* | s00256g14067.t1 | AT1G59640.2 | AT1G22490.1 | 0.564 |
| *s00709g22875.t1* | s00272g14504.t1 | AT1G59640.2 | AT1G31050.1 | 0.57 |
| *s00709g22875.t1* | s00525g20136.t1 | AT1G59640.2 | AT5G53210.1 | 0.44 |
| *s00709g22875.t1* | s01128g27016.t1 | AT1G59640.2 | AT1G09250.1 | 0.47 |
| *s00723g23054.t1* | s00020g02768.t1 | AT1G10610.1 | AT3G50330.1 | 0.574 |
| *s00723g23054.t1* | s00403g17722.t1 | AT1G10610.1 | AT2G42280.1 | 0.438 |
| *s00723g23054.t1* | s00664g22314.t1 | AT1G10610.1 | AT5G01310.1 | 0.699 |
| *s00725g23075.t1* | CPC | AT5G41315.1 | AT2G46410.1 | 0.993 |
| *s00725g23075.t1* | JAZ1 | AT5G41315.1 | AT1G19180.1 | 0.464 |
| *s00725g23075.t1* | MYB75 | AT5G41315.1 | AT1G56650.1 | 0.951 |
| *s00725g23075.t1* | TT2 | AT5G41315.1 | AT5G35550.1 | 0.803 |
| *s00725g23075.t1* | TTG1 | AT5G41315.1 | AT5G24520.1 | 0.996 |
| *s00725g23075.t1* | s00013g01919.t1 | AT5G41315.1 | AT5G50915.1 | 0.451 |
| *s00725g23075.t1* | s00123g09328.t1 | AT5G41315.1 | AT4G33880.1 | 0.499 |
| *s00725g23075.t1* | s00142g10133.t1 | AT5G41315.1 | AT1G66470.1 | 0.638 |
| *s00725g23075.t1* | s00307g15448.t1 | AT5G41315.1 | AT2G14760.3 | 0.439 |
| *s00725g23075.t1* | s01168g27316.t1 | AT5G41315.1 | AT5G54680.1 | 0.411 |
| *s00727g23102.t1* | s00050g05205.t1 | AT1G49770.1 | AT3G26744.1 | 0.889 |
| *s00727g23102.t1* | s00086g07547.t2 | AT1G49770.1 | AT5G64340.1 | 0.431 |
| *s00727g23102.t1* | s00123g09328.t1 | AT1G49770.1 | AT4G33880.1 | 0.653 |
| *s00727g23102.t1* | s00307g15448.t1 | AT1G49770.1 | AT2G14760.3 | 0.653 |
| *s00727g23102.t1* | s00620g21689.t1 | AT1G49770.1 | AT2G31220.1 | 0.617 |
| *s00727g23102.t1* | s00780g23770.t2 | AT1G49770.1 | AT5G56960.1 | 0.612 |
| *s00727g23102.t1* | s01168g27316.t1 | AT1G49770.1 | AT5G54680.1 | 0.499 |
| *s00780g23770.t2* | s00003g00506.t1 | AT5G56960.1 | AT2G28160.1 | 0.586 |
| *s00780g23770.t2* | s00184g11787.t1 | AT5G56960.1 | AT2G46970.1 | 0.497 |
| *s00780g23770.t2* | s00620g21689.t1 | AT5G56960.1 | AT2G31220.1 | 0.831 |
| *s00780g23770.t2* | s00727g23102.t1 | AT5G56960.1 | AT1G49770.1 | 0.612 |
| *s00780g23770.t2* | s00866g24700.t1 | AT5G56960.1 | AT3G17100.1 | 0.441 |
| *s00780g23770.t2* | s01128g27016.t1 | AT5G56960.1 | AT1G09250.1 | 0.587 |
| *s00780g23770.t2* | s01295g28048.t1 | AT5G56960.1 | AT2G46810.1 | 0.776 |
| *s00786g23832.t1* | s00142g10133.t1 | AT4G25400.1 | AT1G66470.1 | 0.424 |
| *s00786g23832.t1* | s00215g12870.t1 | AT4G25400.1 | AT5G58010.1 | 0.422 |
| *s00786g23832.t1* | s00233g13419.t1 | AT4G25400.1 | AT5G57150.4 | 0.577 |
| *s00786g23832.t1* | s00786g23833.t1 | AT4G25400.1 | AT5G51780.1 | 0.424 |
| *s00786g23832.t1* | s01295g28048.t1 | AT4G25400.1 | AT2G46810.1 | 0.734 |
| *s00786g23833.t1* | s00786g23832.t1 | AT5G51780.1 | AT4G25400.1 | 0.424 |
| *s00789g23874.t1* | s00184g11787.t1 | AT3G21330.1 | AT2G46970.1 | 0.432 |
| *s00789g23874.t1* | s00188g11941.t1 | AT3G21330.1 | AT2G41130.1 | 0.531 |
| *s00789g23874.t1* | s00921g25318.t1 | AT3G21330.1 | AT4G20970.1 | 0.482 |
| *s00866g24700.t1* | s00072g06736.t2 | AT3G17100.1 | AT5G62610.1 | 0.74 |
| *s00866g24700.t1* | s00221g13054.t1 | AT3G17100.1 | AT1G26945.1 | 0.535 |
| *s00866g24700.t1* | s00233g13419.t1 | AT3G17100.1 | AT5G57150.4 | 0.441 |
| *s00866g24700.t1* | s00780g23770.t2 | AT3G17100.1 | AT5G56960.1 | 0.441 |
| *s00866g24700.t1* | s01056g26517.t1 | AT3G17100.1 | AT1G68920.1 | 0.524 |
| *s00875g24796.t1* | JAZ1 | AT5G43650.1 | AT1G19180.1 | 0.565 |
| *s00875g24796.t1* | s00003g00506.t1 | AT5G43650.1 | AT2G28160.1 | 0.739 |
| *s00875g24796.t1* | s00050g05205.t1 | AT5G43650.1 | AT3G26744.1 | 0.404 |
| *s00875g24796.t1* | s00111g08792.t1 | AT5G43650.1 | AT1G72210.1 | 0.4 |
| *s00875g24796.t1* | s00233g13419.t1 | AT5G43650.1 | AT5G57150.4 | 0.967 |
| *s00875g24796.t1* | s00256g14067.t1 | AT5G43650.1 | AT1G22490.1 | 0.4 |
| *s00875g24796.t1* | s00408g17845.t1 | AT5G43650.1 | AT2G27230.2 | 0.552 |
| *s00875g24796.t1* | s00620g21689.t1 | AT5G43650.1 | AT2G31220.1 | 0.526 |
| *s00921g25318.t1* | s00013g01919.t1 | AT4G20970.1 | AT5G50915.1 | 0.482 |
| *s00921g25318.t1* | s00789g23874.t1 | AT4G20970.1 | AT3G21330.1 | 0.482 |
| *s00972g25791.t1* | s00025g03213.t1 | AT1G69010.1 | AT5G46690.1 | 0.521 |
| *s00972g25791.t1* | s00111g08792.t1 | AT1G69010.1 | AT1G72210.1 | 0.499 |
| *s00972g25791.t1* | s00135g09872.t1 | AT1G69010.1 | AT1G29950.2 | 0.567 |
| *s00972g25791.t1* | s00233g13419.t1 | AT1G69010.1 | AT5G57150.4 | 0.402 |
| *s00972g25791.t1* | s00442g18537.t2 | AT1G69010.1 | AT2G40200.1 | 0.52 |
| *s01056g26517.t1* | s00171g11322.t1 | AT1G68920.1 | AT1G01260.1 | 0.416 |
| *s01056g26517.t1* | s00221g13054.t1 | AT1G68920.1 | AT1G26945.1 | 0.465 |
| *s01056g26517.t1* | s00233g13419.t1 | AT1G68920.1 | AT5G57150.4 | 0.439 |
| *s01056g26517.t1* | s00866g24700.t1 | AT1G68920.1 | AT3G17100.1 | 0.524 |
| *s01056g26517.t1* | s01128g27016.t1 | AT1G68920.1 | AT1G09250.1 | 0.567 |
| *s01111g26902.t1* | s00025g03213.t1 | AT1G73830.1 | AT5G46690.1 | 0.448 |
| *s01111g26902.t1* | s00086g07547.t2 | AT1G73830.1 | AT5G64340.1 | 0.43 |
| *s01111g26902.t1* | s00268g14415.t1 | AT1G73830.1 | AT1G25330.1 | 0.77 |
| *s01111g26902.t1* | s00442g18537.t2 | AT1G73830.1 | AT2G40200.1 | 0.612 |
| *s01128g27016.t1* | s00076g06984.t1 | AT1G09250.1 | AT1G09530.2 | 0.576 |
| *s01128g27016.t1* | s00171g11322.t1 | AT1G09250.1 | AT1G01260.1 | 0.526 |
| *s01128g27016.t1* | s00184g11787.t1 | AT1G09250.1 | AT2G46970.1 | 0.576 |
| *s01128g27016.t1* | s00233g13419.t1 | AT1G09250.1 | AT5G57150.4 | 0.587 |
| *s01128g27016.t1* | s00403g17722.t1 | AT1G09250.1 | AT2G42280.1 | 0.465 |
| *s01128g27016.t1* | s00709g22875.t1 | AT1G09250.1 | AT1G59640.2 | 0.47 |
| *s01128g27016.t1* | s00780g23770.t2 | AT1G09250.1 | AT5G56960.1 | 0.587 |
| *s01128g27016.t1* | s01056g26517.t1 | AT1G09250.1 | AT1G68920.1 | 0.567 |
| *s01168g27316.t1* | s00003g00506.t1 | AT5G54680.1 | AT2G28160.1 | 0.629 |
| *s01168g27316.t1* | s00031g03756.t1 | AT5G54680.1 | AT4G00050.1 | 0.436 |
| *s01168g27316.t1* | s00050g05205.t1 | AT5G54680.1 | AT3G26744.1 | 0.53 |
| *s01168g27316.t1* | s00052g05353.t1 | AT5G54680.1 | AT3G47640.2 | 0.7 |
| *s01168g27316.t1* | s00312g15577.t1 | AT5G54680.1 | AT4G14410.1 | 0.694 |
| *s01168g27316.t1* | s00546g20503.t1 | AT5G54680.1 | AT3G24140.1 | 0.41 |
| *s01168g27316.t1* | s00725g23075.t1 | AT5G54680.1 | AT5G41315.1 | 0.411 |
| *s01168g27316.t1* | s00727g23102.t1 | AT5G54680.1 | AT1G49770.1 | 0.499 |
| *s01183g27416.t1* | s00052g05353.t1 | AT3G19500.1 | AT3G47640.2 | 0.641 |
| *s01295g28048.t1* | s00438g18457.t1 | AT2G46810.1 | AT4G21330.1 | 0.476 |
| *s01295g28048.t1* | s00583g21099.t1 | AT2G46810.1 | AT1G68810.1 | 0.626 |
| *s01295g28048.t1* | s00620g21689.t1 | AT2G46810.1 | AT2G31220.1 | 0.782 |
| *s01295g28048.t1* | s00780g23770.t2 | AT2G46810.1 | AT5G56960.1 | 0.776 |
| *s01295g28048.t1* | s00786g23832.t1 | AT2G46810.1 | AT4G25400.1 | 0.734 |
| *s01354g28377.t1* | CKS2 | AT2G43140.2 | AT2G27970.1 | 0.995 |
| *s01354g28377.t1* | KRP2 | AT2G43140.2 | AT3G50630.1 | 0.995 |
| *s01354g28377.t1* | s00171g11322.t1 | AT2G43140.2 | AT1G01260.1 | 0.61 |
| *s01408g28647.t1* | s00052g05353.t1 | AT3G07340.1 | AT3G47640.2 | 0.479 |
| *s01408g28647.t1* | s00173g11418.t1 | AT3G07340.1 | AT2G31730.1 | 0.437 |
| *s01408g28647.t1* | s00212g12766.t1 | AT3G07340.1 | AT5G65640.1 | 0.439 |
| *s01408g28647.t1* | s00272g14504.t1 | AT3G07340.1 | AT1G31050.1 | 0.521 |

**Table S8. Primer sequences used in amplification, qPCR and yeast one-hybrid**

| **No.** | **Named** | **Primers Sequence** | |
| --- | --- | --- | --- |
|  |  | **Forward (5’-3’)** | **Reverse (5’-3’)** |
| **1** | **HT -** FaMYB10 | **TCAAATCAGGCTTAAACAGA** | **TTAAAGACCACCTGTTTCCT** |
| **2** | **HT -** FcbHLH42 | **CAATGGAGCAATCAAGACAA** | **GAGACGCACATCAAGTAGA** |
| **3** | **qPCR -** NbTubA1 | **CTCATATGCTCCTGTCATTTC** | **GACGAGGGTCACACTTAAC** |
| **4** | **qPCR -** NbF3H | **CAAGGCTTGTGTGGATATGG** | **TATGTCGTTTCAGCCCAAGT** |
| **5** | **qPCR -** NbDFR | **AACCAACAGTCAGGGGAATG** | **TTGGACATCGAGAGTTCCAG** |
| **6** | **qPCR -** NbANS | **TGGCGTTGAAGCTCATACTG** | **GGAATTAGGCACACACTTTGC** |
| **7** | **qPCR -** NbUFGT | **ATGAGTGCATTGGATACTTT** | **AGCTCCATTAGATCCTTGAA** |
| **8** | **qPCR -** FaMYB10 | **TCAAATCAGGCTTAAACAGA** | **TTAAAGACCACCTGTTTCCT** |
| **9** | **qPCR -** FcbHLH42 | **CAATGGAGCAATCAAGACAA** | **GAGACGCACATCAAGTAGA** |
| **10** | **pGAD -** FcbHLH42 | **gccgctgcaggtcgacgggATGGCTGCACCGCCGAGTGG** | **ttcatctgcagctcgagctcTTATCTCCAAGTGTCACTGTGG** |
| **11** | **pGAD -** FcbHLH3 | **gccgctgcaggtcgacgggATGAGTGAAAAATTTTGGGTGAATG** | **ttcatctgcagctcgagctcTCATTTCGAAAAAGCAGCCAG** |
| **12** | **pGAD -** FcMYC2 | **gccgctgcaggtcgacgggATGAGCTCCTCCGATCTCTC** | **ttcatctgcagctcgagctcTTATCGGGCAACGCCGCCGA** |
| **13** | **pGAD -** FcbHLH14 | **gccgctgcaggtcgacgggATGGAAGACCTAATGATATCGCCAT** | **ttcatctgcagctcgagctcTTATAGCTCTAATCTTCTGAGAAGAG** |
| **14** | **pGBD -** FaMYB10 | **catatggccatggaggccgaattcATGGAGGGTTTCGGTGTGAG** | **gccgctgcaggtcgacggatccTCATACGTAGGAGATGTTGA** |

**Supplementary Table S9. Co-expression combinations of FcbHLH42 and TFs**

| Source | Target1 | RNA-seq | Weight | direction | Group | Genome ID | Predicted Gene |
| --- | --- | --- | --- | --- | --- | --- | --- |
| FcbHLH42 | s00097g08109.t1 | c12586_g1 | 0.64029977 | undirection | MYB | s00097g08109.t1 | s00097g08109.t1 |
| FcbHLH42 | FcMYB4-1 | c21359_g1 | 0.644812146 | undirection | MYB | s00039g04404.t1 | FcMYB4-1 |
| FcbHLH42 | FcMYB123 | c23208_g1 | 0.641264255 | undirection | MYB | s00298g15169.t2 | FcMYB123 |
| FcbHLH42 | s00020g02724.t1 | c25715_g1 | 0.623499604 | undirection | MYB | s00020g02724.t1 | s00020g02724.t1 |
| FcbHLH42 | s01067g26598.t1 | c25715_g2 | 0.620953131 | undirection | MYB | s01067g26598.t1 | s01067g26598.t1 |
| FcbHLH42 | FcWER | c29346_g2 | 0.646549432 | undirection | MYB | s00374g17095.t1 | FcWER |
| FcbHLH42 | FcCPC | c31006_g1 | 0.700539848 | undirection | MYB | s00910g25196.t1 | FcCPC |
| FcbHLH42 | s00013g01949.t1 | c33707_g1 | 0.653495864 | undirection | MYB | s00013g01949.t1 | s00013g01949.t1 |
| FcbHLH42 | s00775g23711.t1 | c33961_g1 | 0.686299638 | undirection | MYB | s00775g23711.t1 | s00775g23711.t1 |
| FcbHLH42 | s04917g31721.t1 | c36150_g1 | 0.622424073 | undirection | MYB | s04917g31721.t1 | s04917g31721.t1 |
| FcbHLH42 | FcMYB1R1 | c36406_g1 | 0.576277851 | undirection | MYB | s00072g06725.t1 | FcMYB1R1 |
| FcbHLH42 | s00149g10489.t1 | c36789_g1 | 0.645541093 | undirection | MYB | s00149g10489.t1 | s00149g10489.t1 |
| FcbHLH42 | FcMYB5-1 | c37406_g4 | 0.641356348 | undirection | MYB | s00309g15486.t1 | FcMYB5-1 |
| FcbHLH42 | s00457g18841.t1 | c38052_g1 | 0.644621092 | undirection | MYB | s00457g18841.t1 | s00457g18841.t1 |
| FcbHLH42 | s00207g12578.t1 | c38069_g3 | 0.648780779 | undirection | MYB | s00207g12578.t1 | s00207g12578.t1 |
| FcbHLH42 | s00060g05936.t1 | c38664_g2 | 0.605655205 | undirection | MYB | s00060g05936.t1 | s00060g05936.t1 |
| FcbHLH42 | s00014g02094.t1 | c39666_g1 | 0.578374994 | undirection | MYB | s00014g02094.t1 | s00014g02094.t1 |
| FcbHLH42 | FcMYBL2 | c39878_g1 | 0.645590055 | undirection | MYB | s00142g10126.t1 | FcMYBL2 |
| FcbHLH42 | s00308g15483.t1 | c40507_g1 | 0.626294571 | undirection | MYB | s00308g15483.t1 | s00308g15483.t1 |
| FcbHLH42 | FcMYB114 | c42269_g1 | 0.644972526 | undirection | MYB | FcMYB114 | FcMYB114 |
| FcbHLH42 | s01681g29668.t1 | c43673_g1 | 0.641758531 | undirection | MYB | s01681g29668.t1 | s01681g29668.t1 |
| FcbHLH42 | MYB5-2 | c43875_g3 | 0.645061795 | undirection | MYB | s01081g26695.t1 | MYB5-2 |
| FcbHLH42 | s01081g26695.t1 | c45040_g1 | 0.643591463 | undirection | MYB | s01081g26695.t1 | s01081g26695.t1 |
| FcbHLH42 | s00845g24485.t1 | c1674_g1 | 0.620105551 | undirection | WD40 | s00845g24485.t1 | s00845g24485.t1 |
| FcbHLH42 | s00857g24606.t1 | c17415_g1 | 0.633770249 | undirection | WD40 | s00857g24606.t1 | s00857g24606.t1 |
| FcbHLH42 | s00152g10622.t1 | c25322_g2 | 0.61838464 | undirection | WD40 | s00152g10622.t1 | s00152g10622.t1 |
| FcbHLH42 | s00036g04144.t1 | c26804_g1 | 0.594978268 | undirection | WD40 | s00036g04144.t1 | s00036g04144.t1 |
| FcbHLH42 | s00045g04876.t1 | c27872_g2 | 0.639530892 | undirection | WD40 | s00045g04876.t1 | s00045g04876.t1 |
| FcbHLH42 | s00299g15229.t1 | c30004_g2 | 0.625474333 | undirection | WD40 | s00299g15229.t1 | s00299g15229.t1 |
| FcbHLH42 | s00080g07237.t1 | c31340_g2 | 0.594998054 | undirection | WD40 | s00080g07237.t1 | s00080g07237.t1 |
| FcbHLH42 | s00008g01376.t1 | c38775_g1 | 0.649505221 | undirection | WD40 | s00008g01376.t1 | s00008g01376.t1 |
| FcbHLH42 | s00179g11599.t1 | c39249_g1 | 0.553866463 | undirection | WD40 | s00179g11599.t1 | s00179g11599.t1 |
| FcbHLH42 | s15933g34294.t1 | c41731_g1 | 0.52113849 | undirection | WD40 | s15933g34294.t1 | s15933g34294.t1 |
| FcbHLH42 | s00252g13959.t1 | c41864_g2 | 0.601743094 | undirection | WD40 | s00252g13959.t1 | s00252g13959.t1 |
| FcbHLH42 | s00516g19974.t1 | c42300_g1 | 0.620395938 | undirection | WD40 | s00516g19974.t1 | s00516g19974.t1 |
| FcbHLH42 | s00534g20295.t1 | c43382_g1 | 0.652170838 | undirection | WD40 | s00534g20295.t1 | s00534g20295.t1 |
| FcbHLH42 | s00097g08105.t1 | c44001_g2 | 0.6358967 | undirection | WD40 | s00097g08105.t1 | s00097g08105.t1 |
| FcbHLH42 | s00409g17849.t1 | c46056_g1 | 0.682108125 | undirection | WD40 | s00409g17849.t1 | s00409g17849.t1 |
| FcbHLH42 | s00040g04471.t1 | c47034_g1 | 0.639203134 | undirection | WD40 | s00040g04471.t1 | s00040g04471.t1 |
| FcbHLH42 | s00156g10751.t1 | c47043_g1 | 0.625867848 | undirection | WD40 | s00156g10751.t1 | s00156g10751.t1 |
| FcbHLH42 | s01952g30311.t1 | c47220_g2 | 0.624825919 | undirection | WD40 | s01952g30311.t1 | s01952g30311.t1 |
| FcbHLH42 | s01147g27169.t1 | c54024_g1 | 0.620142842 | undirection | WD40 | s01147g27169.t1 | s01147g27169.t1 |
| FcbHLH42 | s00090g07728.t1 | c54093_g1 | 0.57265799 | undirection | WD40 | s00090g07728.t1 | s00090g07728.t1 |
| FcbHLH42 | s00098g08138.t1 | c65890_g1 | 0.558269795 | undirection | WD40 | s00098g08138.t1 | s00098g08138.t1 |
| FcbHLH42 | s00126g09478.t1 | c66020_g1 | 0.54408024 | undirection | WD40 | s00126g09478.t1 | s00126g09478.t1 |
| FcbHLH42 | FcbHLH10 | c33434_g2 | 0.623258218 | undirection | bHLH | s00403g17722.t1 | FcbHLH10 |
| FcbHLH42 | FcbHLH69 | c40876_g3 | 0.707532809 | undirection | bHLH | s00240g13612.t1 | FcbHLH69 |
| FcbHLH42 | FcbHLH11 | c44069_g1 | 0.608439738 | undirection | bHLH | s01354g28377.t1 | FcbHLH11 |
| FcbHLH42 | FcbHLH117 | c78643_g1 | 0.593995702 | undirection | bHLH | s01128g27016.t1 | FcbHLH117 |
| FcbHLH42 | FcDFR | c40280_g1 | 0.648824806 | undirection | SG | s00004g00771.t1 | FcDFR |
| FcbHLH42 | FcCHI1 | c41642_g1 | 0.631870446 | undirection | SG | s17336g34558.t1 | FcCHI1 |
| FcbHLH42 | FcF3'H1 | c42263_g2 | 0.648878708 | undirection | SG | s11133g33345.t1 | FcF3'H1 |
| FcbHLH42 | FcF3'H2 | c42263_g3 | 0.655222804 | undirection | SG | s11133g33345.t1 | FcF3'H2 |
| FcbHLH42 | FcF3H | c43823_g1 | 0.673846568 | undirection | SG | s01343g28319.t1 | FcF3H |
| FcbHLH42 | FcUFGT1 | c45009_g5 | 0.645030762 | undirection | SG | s00107g08570.t1 | FcUFGT1 |
| FcbHLH42 | FcCHS1 | c46769_g2 | 0.642858658 | undirection | SG | s00164g11049.t1 | FcCHS1 |
| FcbHLH42 | FcCHS2 | c46769_g3 | 0.644406284 | undirection | SG | s00164g11050.t1 | FcCHS2 |
| FcbHLH42 | FcCHI2 | c47235_g1 | 0.634983044 | undirection | SG | s00077g07012.t1 | FcCHI2 |
| FcbHLH42 | FcANS | c59676_g1 | 0.647764579 | undirection | SG | s00061g06011.t1 | FcANS |
| FcbHLH42 | FcUFGT2 | c78174_g2 | 0.637701043 | undirection | SG | s00325g15919.t1 | FcUFGT2 |
